# Physical distancing, face masks, and eye protection to prevent person-to-person transmission of SARS-CoV-2 and COVID-19: a systematic review and meta-analysis

**DOI:** 10.1016/S0140-6736(20)31142-9

**Published:** 2020-06-27

**Authors:** Derek K Chu, Elie A Akl, Stephanie Duda, Karla Solo, Sally Yaacoub, Holger J Schünemann, COVID-19 Systematic Urgent Review Group Effort (SURGE) study authors, Derek K Chu, Elie A Akl, Amena El-harakeh, Antonio Bognanni, Tamara Lotfi, Mark Loeb, Anisa Hajizadeh, Anna Bak, Ariel Izcovich, Carlos A Cuello-Garcia, Chen Chen, David J Harris, Ewa Borowiack, Fatimah Chamseddine, Finn Schünemann, Gian Paolo Morgano, Giovanna E U Muti Schünemann, Guang Chen, Hong Zhao, Ignacio Neumann, Jeffrey Chan, Joanne Khabsa, Layal Hneiny, Leila Harrison, Maureen Smith, Nesrine Rizk, Paolo Giorgi Rossi, Pierre AbiHanna, Rayane El-khoury, Rosa Stalteri, Tejan Baldeh, Thomas Piggott, Yuan Zhang, Zahra Saad, Assem Khamis, Marge Reinap, Stephanie Duda, Karla Solo, Sally Yaacoub, Holger J Schünemann

**Affiliations:** a Department of Health Research Methods, Evidence and Impact, McMaster University, Hamilton, ON, Canada; b Department of Medicine, McMaster University, Hamilton, ON, Canada; c The Research Institute of St Joe's Hamilton, Hamilton, ON, Canada; d Department of Internal Medicine, American University of Beirut, Beirut, Lebanon; e Clinical Research Institute, American University of Beirut, Beirut, Lebanon; f Michael G DeGroote Cochrane Canada and GRADE Centres, Hamilton, ON, Canada

## Abstract

**Background:**

Severe acute respiratory syndrome coronavirus 2 (SARS-CoV-2) causes COVID-19 and is spread person-to-person through close contact. We aimed to investigate the effects of physical distance, face masks, and eye protection on virus transmission in health-care and non-health-care (eg, community) settings.

**Methods:**

We did a systematic review and meta-analysis to investigate the optimum distance for avoiding person-to-person virus transmission and to assess the use of face masks and eye protection to prevent transmission of viruses. We obtained data for SARS-CoV-2 and the betacoronaviruses that cause severe acute respiratory syndrome, and Middle East respiratory syndrome from 21 standard WHO-specific and COVID-19-specific sources. We searched these data sources from database inception to May 3, 2020, with no restriction by language, for comparative studies and for contextual factors of acceptability, feasibility, resource use, and equity. We screened records, extracted data, and assessed risk of bias in duplicate. We did frequentist and Bayesian meta-analyses and random-effects meta-regressions. We rated the certainty of evidence according to Cochrane methods and the GRADE approach. This study is registered with PROSPERO, CRD42020177047.

**Findings:**

Our search identified 172 observational studies across 16 countries and six continents, with no randomised controlled trials and 44 relevant comparative studies in health-care and non-health-care settings (n=25 697 patients). Transmission of viruses was lower with physical distancing of 1 m or more, compared with a distance of less than 1 m (n=10 736, pooled adjusted odds ratio [aOR] 0·18, 95% CI 0·09 to 0·38; risk difference [RD] −10·2%, 95% CI −11·5 to −7·5; moderate certainty); protection was increased as distance was lengthened (change in relative risk [RR] 2·02 per m; *p*_interaction_=0·041; moderate certainty). Face mask use could result in a large reduction in risk of infection (n=2647; aOR 0·15, 95% CI 0·07 to 0·34, RD −14·3%, −15·9 to −10·7; low certainty), with stronger associations with N95 or similar respirators compared with disposable surgical masks or similar (eg, reusable 12–16-layer cotton masks; *p*_interaction_=0·090; posterior probability >95%, low certainty). Eye protection also was associated with less infection (n=3713; aOR 0·22, 95% CI 0·12 to 0·39, RD −10·6%, 95% CI −12·5 to −7·7; low certainty). Unadjusted studies and subgroup and sensitivity analyses showed similar findings.

**Interpretation:**

The findings of this systematic review and meta-analysis support physical distancing of 1 m or more and provide quantitative estimates for models and contact tracing to inform policy. Optimum use of face masks, respirators, and eye protection in public and health-care settings should be informed by these findings and contextual factors. Robust randomised trials are needed to better inform the evidence for these interventions, but this systematic appraisal of currently best available evidence might inform interim guidance.

**Funding:**

World Health Organization.

## Introduction

As of May 28, 2020, severe acute respiratory syndrome coronavirus 2 (SARS-CoV-2) has infected more than 5·85 million individuals worldwide and caused more than 359 000 deaths.^1^ Emergency lockdowns have been initiated in countries across the globe, and the effect on health, wellbeing, business, and other aspects of daily life are felt throughout societies and by individuals. With no effective pharmacological interventions or vaccine available in the imminent future, reducing the rate of infection (ie, flattening the curve) is a priority, and prevention of infection is the best approach to achieve this aim.

SARS-CoV-2 spreads person-to-person through close contact and causes COVID-19. It has not been solved if SARS-CoV-2 might spread through aerosols from respiratory droplets; so far, air sampling has found virus RNA in some studies^2^, ^3^, ^4^ but not in others.^5^, ^6^, ^7^, ^8^ However, finding RNA virus is not necessarily indicative of replication-competent and infection-competent (viable) virus that could be transmissible. The distance from a patient that the virus is infective, and the optimum person-to-person physical distance, is uncertain. For the currently foreseeable future (ie, until a safe and effective vaccine or treatment becomes available), COVID-19 prevention will continue to rely on non-pharmaceutical interventions, including pandemic mitigation in community settings.^9^ Thus, quantitative assessment of physical distancing is relevant to inform safe interaction and care of patients with SARS-CoV-2 in both health-care and non-health-care settings. The definition of close contact or potentially exposed helps to risk stratify, contact trace, and develop guidance documents, but these definitions differ around the globe.

**Research in context**

**Evidence before this study**

We searched 21 databases and resources from inception to May 3, 2020, with no restriction by language, for studies of any design evaluating physical distancing, face masks, and eye protection to prevent transmission of the viruses that cause COVID-19 and related diseases (eg, severe acute respiratory syndrome [SARS] and Middle East respiratory syndrome [MERS]) between infected individuals and people close to them (eg, household members, caregivers, and health-care workers). Previous related meta-analyses have focused on randomised trials and reported imprecise data for common respiratory viruses such as seasonal influenza, rather than the pandemic and epidemic betacoronaviruses causative of COVID-19 (severe acute respiratory syndrome coronavirus 2 [SARS-CoV-2]), SARS (SARS-CoV), or MERS (MERS-CoV). Other meta-analyses have focused on interventions in the health-care setting and have not included non-health-care (eg, community) settings. Our search did not retrieve any systematic review of information on physical distancing, face masks, or eye protection to prevent transmission of SARS-CoV-2, SARS-CoV, and MERS-CoV.

**Added value of this study**

We did a systematic review of 172 observational studies in health-care and non-health-care settings across 16 countries and six continents; 44 comparative studies were included in a meta-analysis, including 25 697 patients with COVID-19, SARS, or MERS. Our findings are, to the best of our knowledge, the first to rapidly synthesise all direct information on COVID-19 and, therefore, provide the best available evidence to inform optimum use of three common and simple interventions to help reduce the rate of infection and inform non-pharmaceutical interventions, including pandemic mitigation in non-health-care settings. Physical distancing of 1 m or more was associated with a much lower risk of infection, as was use of face masks (including N95 respirators or similar and surgical or similar masks [eg, 12–16-layer cotton or gauze masks]) and eye protection (eg, goggles or face shields). Added benefits are likely with even larger physical distances (eg, 2 m or more based on modelling) and might be present with N95 or similar respirators versus medical masks or similar. Across 24 studies in health-care and non-health-care settings of contextual factors to consider when formulating recommendations, most stakeholders found these personal protection strategies acceptable, feasible, and reassuring but noted harms and contextual challenges, including frequent discomfort and facial skin breakdown, high resource use linked with the potential to decrease equity, increased difficulty communicating clearly, and perceived reduced empathy of care providers by those they were caring for.

**Implications of all the available evidence**

In view of inconsistent guidelines by various organisations based on limited information, our findings provide some clarification and have implications for multiple stakeholders. The risk for infection is highly dependent on distance to the individual infected and the type of face mask and eye protection worn. From a policy and public health perspective, current policies of at least 1 m physical distancing seem to be strongly associated with a large protective effect, and distances of 2 m could be more effective. These data could also facilitate harmonisation of the definition of exposed (eg, within 2 m), which has implications for contact tracing. The quantitative estimates provided here should inform disease-modelling studies, which are important for planning pandemic response efforts. Policy makers around the world should strive to promptly and adequately address equity implications for groups with currently limited access to face masks and eye protection. For health-care workers and administrators, our findings suggest that N95 respirators might be more strongly associated with protection from viral transmission than surgical masks. Both N95 and surgical masks have a stronger association with protection compared with single-layer masks. Eye protection might also add substantial protection. For the general public, evidence shows that physical distancing of more than 1 m is highly effective and that face masks are associated with protection, even in non-health-care settings, with either disposable surgical masks or reusable 12–16-layer cotton ones, although much of this evidence was on mask use within households and among contacts of cases. Eye protection is typically underconsidered and can be effective in community settings. However, no intervention, even when properly used, was associated with complete protection from infection. Other basic measures (eg, hand hygiene) are still needed in addition to physical distancing and use of face masks and eye protection.

To contain widespread infection and to reduce morbidity and mortality among health-care workers and others in contact with potentially infected people, jurisdictions have issued conflicting advice about physical or social distancing. Use of face masks with or without eye protection to achieve additional protection is debated in the mainstream media and by public health authorities, in particular the use of face masks for the general population;^10^ moreover, optimum use of face masks in health-care settings, which have been used for decades for infection prevention, is facing challenges amid personal protective equipment (PPE) shortages.^11^

Any recommendations about social or physical distancing, and the use of face masks, should be based on the best available evidence. Evidence has been reviewed for other respiratory viral infections, mainly seasonal influenza,^12^, ^13^ but no comprehensive review is available of information on SARS-CoV-2 or related betacoronaviruses that have caused epidemics, such as severe acute respiratory syndrome (SARS) or Middle East respiratory syndrome (MERS). We, therefore, systematically reviewed the effect of physical distance, face masks, and eye protection on transmission of SARS-CoV-2, SARS-CoV, and MERS-CoV.

## Methods

### Search strategy and selection criteria

To inform WHO guidance documents, on March 25, 2020, we did a rapid systematic review.^14^ We created a large international collaborative and we used Cochrane methods^15^ and the GRADE approach.^16^ We prospectively submitted the systematic review protocol for registration on PROSPERO (CRD42020177047; appendix pp 23–29). We have followed PRISMA^17^ and MOOSE^18^ reporting guidelines (appendix pp 30–33).

From database inception to May 3, 2020, we searched for studies of any design and in any setting that included patients with WHO-defined confirmed or probable COVID-19, SARS, or MERS, and people in close contact with them, comparing distances between people and COVID-19 infected patients of 1 m or larger with smaller distances, with or without a face mask on the patient, or with or without a face mask, eye protection, or both on the exposed individual. The aim of our systematic review was for quantitative assessment to ascertain the physical distance associated with reduced risk of acquiring infection when caring for an individual infected with SARS-CoV-2, SARS-CoV, or MERS-CoV. Our definition of face masks included surgical masks and N95 respirators, among others; eye protection included visors, faceshields, and goggles, among others.

We searched (up to March 26, 2020) MEDLINE (using the Ovid platform), PubMed, Embase, CINAHL (using the Ovid platform), the Cochrane Library, COVID-19 Open Research Dataset Challenge, COVID-19 Research Database (WHO), Epistemonikos (for relevant systematic reviews addressing MERS and SARS, and its COVID-19 Living Overview of the Evidence platform), EPPI Centre living systematic map of the evidence, ClinicalTrials.gov, WHO International Clinical Trials Registry Platform, relevant documents on the websites of governmental and other relevant organisations, reference lists of included papers, and relevant systematic reviews.^19^, ^20^ We handsearched (up to May 3, 2020) preprint servers (bioRxiv, medRxiv, and Social Science Research Network First Look) and coronavirus resource centres of *The Lancet, JAMA*, and *N Engl J Med* (appendix pp 3–5). We did not limit our search by language. We initially could not obtain three full texts for evaluation, but we obtained them through interlibrary loan or contacting a study author. We did not restrict our search to any quantitative cutoff for distance.

### Data collection

We screened titles and abstracts, reviewed full texts, extracted data, and assessed risk of bias by two authors and independently, using standardised prepiloted forms (Covidence; Veritas Health Innovation, Melbourne, VIC, Australia), and we cross-checked screening results using artificial intelligence (Evidence Prime, Hamilton, ON, Canada). We resolved disagreements by consensus. We extracted data for study identifier, study design, setting, population characteristics, intervention and comparator characteristics, quantitative outcomes, source of funding and reported conflicts of interests, ethics approval, study limitations, and other important comments.

### Outcomes

Outcomes of interest were risk of transmission (ie, WHO-defined confirmed or probable COVID-19, SARS, or MERS) to people in health-care or non-health-care settings by those infected; hospitalisation; intensive care unit admission; death; time to recovery; adverse effects of interventions; and contextual factors such as acceptability, feasibility, effect on equity, and resource considerations related to the interventions of interest. However, data were only available to analyse intervention effects for transmission and contextual factors. Consistent with WHO, studies generally defined confirmed cases with laboratory confirmation (with or without symptoms) and probable cases with clinical evidence of the respective infection (ie, suspected to be infected) but for whom confirmatory testing either had not yet been done for any reason or was inconclusive.

### Data analysis

Our search did not identify any randomised trials of COVID-19, SARS, or MERS. We did a meta-analysis of associations by pooling risk ratios (RRs) or adjusted odds ratios (aORs) depending on availability of these data from observational studies, using DerSimonian and Laird random-effects models. We adjusted for variables including age, sex, and severity of source case; these variables were not the same across studies. Because between-study heterogeneity can be misleadingly large when quantified by *I*^2^ during meta-analysis of observational studies,^21^, ^22^ we used GRADE guidance to assess between-study heterogeneity.^21^ Throughout, we present RRs as unadjusted estimates and aORs as adjusted estimates.

We used the Newcastle-Ottawa scale to rate risk of bias for comparative non-randomised studies corresponding to every study's design (cohort or case-control).^23^, ^24^ We planned to use the Cochrane Risk of Bias tool 2.0 for randomised trials,^25^ but our search did not identify any eligible randomised trials. We synthesised data in both narrative and tabular formats. We graded the certainty of evidence using the GRADE approach. We used the GRADEpro app to rate evidence and present it in GRADE evidence profiles and summary of findings tables^26^, ^27^ using standardised terms.^28^, ^29^

We analysed data for subgroup effects by virus type, intervention (different distances or face mask types), and setting (health care *vs* non-health care). Among the studies assessing physical distancing measures to prevent viral transmission, the intervention varied (eg, direct physical contact [0 m], 1 m, or 2 m). We, therefore, analysed the effect of distance on the size of the associations by random-effects univariate meta-regressions, using restricted maximum likelihood, and we present mean effects and 95% CIs. We calculated tests for interaction using a minimum of 10 000 Monte Carlo random permutations to avoid spurious findings.^30^ We formally assessed the credibility of potential effect-modifiers using GRADE guidance.^21^ We did two sensitivity analyses to test the robustness of our findings. First, we used Bayesian meta-analyses to reinterpret the included studies considering priors derived from the effect point estimate and variance from a meta-analysis of ten randomised trials evaluating face mask use versus no face mask use to prevent influenza-like illness in health-care workers.^31^ Second, we used Bayesian meta-analyses to reinterpret the efficacy of N95 respirators versus medical masks on preventing influenza-like illness after seasonal viral (mostly influenza) infection.^13^ For these sensitivity analyses, we used hybrid Metropolis-Hastings and Gibbs sampling, a 10 000 sample burn-in, 40 000 Markov chain Monte Carlo samples, and we tested non-informative and sceptical priors (eg, four time variance)^32^, ^33^ to inform mean estimates of effect, 95% credibility intervals (CrIs), and posterior distributions. We used non-informative hyperpriors to estimate statistical heterogeneity. Model convergence was confirmed in all cases with good mixing in visual inspection of trace plots, autocorrelation plots, histograms, and kernel density estimates in all scenarios. Parameters were blocked, leading to acceptance of approximately 50% and efficiency greater than 1% in all cases (typically about 40%). We did analyses using Stata version 14.3.

### Role of the funding source

The funder contributed to defining the scope of the review but otherwise had no role in study design and data collection. Data were interpreted and the report drafted and submitted without funder input, but according to contractual agreement, the funder provided review at the time of final publication. The corresponding author had full access to all data in the study and had final responsibility for the decision to submit for publication.

## Results

**Figure 1:**
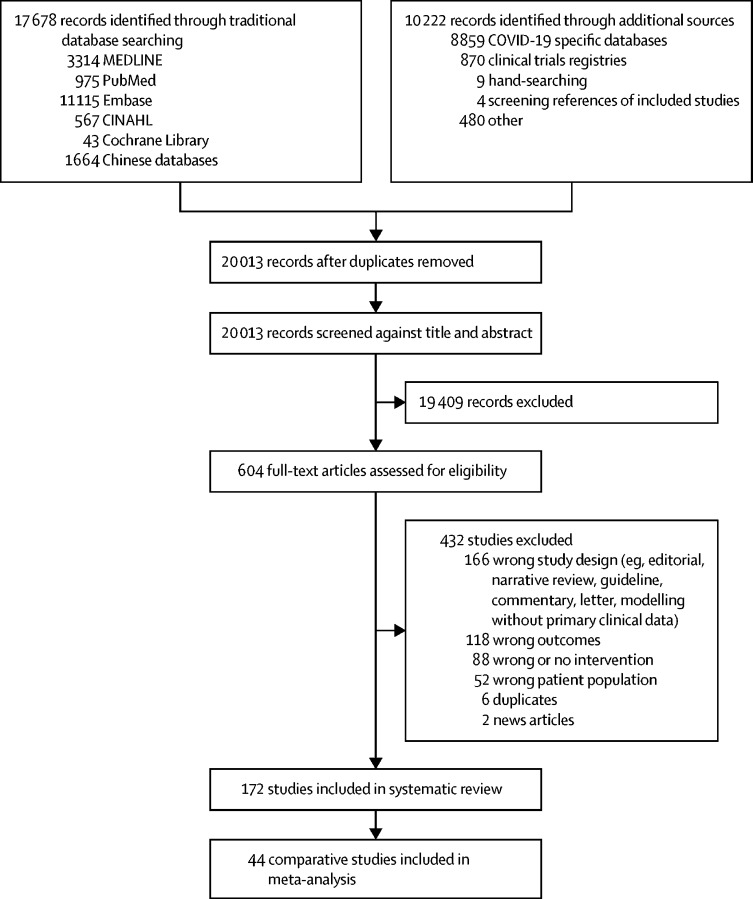
Study selection

We identified 172 studies for our systematic review from 16 countries across six continents (figure 1; appendix pp 6–14, 41–47). Studies were all observational in nature; no randomised trials were identified of any interventions that directly addressed the included study populations. Of the 172 studies, 66 focused on how far a virus can travel by comparing the association of different distances on virus transmission to people (appendix pp 42–44). Of these 66 studies, five were mechanistic, assessing viral RNA, virions, or both cultured from the environment of an infected patient (appendix p 45).

44 studies were comparative^34^, ^35^, ^36^, ^37^, ^38^, ^39^, ^40^, ^41^, ^42^, ^43^, ^44^, ^45^, ^46^, ^47^, ^48^, ^49^, ^50^, ^51^, ^52^, ^53^, ^54^, ^55^, ^56^, ^57^, ^58^, ^59^, ^60^, ^61^, ^62^, ^63^, ^64^, ^65^, ^66^, ^67^, ^68^, ^69^, ^70^, ^71^, ^72^, ^73^, ^74^, ^75^, ^76^, ^77^ and fulfilled criteria for our meta-analysis (n=25 697; figure 1; table 1). We used these studies rather than case series and qualitative studies (appendix pp 41–47) to inform estimates of effect. 30 studies^34^, ^37^, ^41^, ^42^, ^43^, ^44^, ^45^, ^47^, ^48^, ^49^, ^50^, ^51^, ^53^, ^54^, ^55^, ^56^, ^58^, ^59^, ^60^, ^61^, ^64^, ^65^, ^66^, ^67^, ^68^, ^69^, ^70^, ^72^, ^74^, ^75^ focused on the association between use of various types of face masks and respirators by health-care workers, patients, or both with virus transmission. 13 studies^34^, ^37^, ^38^, ^39^, ^47^, ^49^, ^51^, ^54^, ^58^, ^60^, ^61^, ^65^, ^75^ addressed the association of eye protection with virus transmission.

**Table 1:** Characteristics of included comparative studies

	**n**	**Country**	**Setting**	**Disease caused by virus**	**Case definition (WHO)**	**Adjusted estimates**	Risk of bias*
Alraddadi et al (2016)^34^	283	Saudi Arabia	Health care	MERS	Confirmed	Yes	********
Arwady et al (2016)^35^	79	Saudi Arabia	Non-health care (household and family contacts)	MERS	Confirmed	No	******
Bai et al (2020)^36^	118	China	Health care	COVID-19	Confirmed	No	*****
Burke et al (2020)^37^	338	USA	Health care and non-health care (including household and community)	COVID-19	Confirmed	No	****
Caputo et al (2006)^38^	33	Canada	Health care	SARS	Confirmed	No	*****
Chen et al (2009)^39^	758	China	Health care	SARS	Confirmed	Yes	*******
Cheng et al (2020)^40^	226	China	Non-health care (household and family contacts)	COVID-19	Confirmed	No	******
Ha et al (2004)^42^	117	Vietnam	Health care	SARS	Confirmed	No	**
Hall et al (2014)^43^	48	Saudi Arabia	Health care	MERS	Confirmed	No	***
Heinzerling et al (2020)^44^	37	USA	Health care	COVID-19	Confirmed	No	****
Ho et al (2004)^45^	372	Taiwan	Health care	SARS	Confirmed	No	********
Ki et al (2019)^47^	446	South Korea	Health care	MERS	Confirmed	No	******
Kim et al (2016)^48^	9	South Korea	Health care	MERS	Confirmed	No	*****
Kim et al (2016)^49^	1169	South Korea	Health care	MERS	Confirmed	No	******
Lau et al (2004)^50^	2270	China	Non-health care (households)	SARS	Probable	Yes	******
Liu et al (2009)^51^	477	China	Health care	SARS	Confirmed	Yes	*****
Liu et al (2020)^52^	20	China	Non-health care (close contacts)	COVID-19	Confirmed	No	*******
Loeb et al (2004)^53^	43	Canada	Health care	SARS	Confirmed	No	**
Ma et al (2004)^54^	426	China	Health care	SARS	Confirmed	Yes	*********
Nishiura et al (2005)^55^	115	Vietnam	Health care	SARS	Confirmed	Yes	********
Nishiyama et al (2008)^56^	146	Vietnam	Health care	SARS	Confirmed	Yes	******
Olsen et al (2003)^57^	304	China	Non-health care (airplane)	SARS	Confirmed	No	******
Park et al (2004)^58^	110	USA	Health care	SARS	Confirmed	No	**********
Park et al (2016)^59^	80	South Korea	Health care	MERS	Confirmed and probable	No	***
Peck et al (2004)^60^	26	USA	Health care	SARS	Confirmed	No	*********
Pei et al (2006)^61^	443	China	Health care	SARS	Confirmed	No	********
Rea et al (2007)^62^	8662	Canada	Non-health care (community contacts)	SARS	Probable	No	****
Reuss et al (2014)^63^	81	Germany	Health care	MERS	Confirmed	No	*****
Reynolds et al (2006)^64^	153	Vietnam	Health care	SARS	Confirmed	No	***
Ryu et al (2019)^65^	34	South Korea	Health care	MERS	Confirmed	No	******
Scales et al (2003)^66^	69	Canada	Health care	SARS	Probable	No	**
Seto et al (2003)^67^	254	China	Health care	SARS	Confirmed	Yes	********
Teleman et al (2004)^68^	86	Singapore	Health care	SARS	Confirmed	Yes	********
Tuan et al (2007)^69^	212	Vietnam	Non-health care (household and community contacts)	SARS	Confirmed	Yes	******
Van Kerkhove et al (2019)^46^	828	Saudi Arabia	Non-health care (dormitory)	MERS	Confirmed	Yes	********
Wang et al (2020)^41^	493	China	Health care	COVID-19	Confirmed	Yes	****
Wang et al (2020)^70^	5442	China	Health care	COVID-19	Confirmed	No	*****
Wiboonchutikul et al (2016)^71^	38	Thailand	Health care	MERS	Confirmed	No	*****
Wilder-Smith et al (2005)^72^	80	Singapore	Health care	SARS	Confirmed	No	********
Wong et al (2004)^73^	66	China	Health care	SARS	Confirmed	No	*****
Wu et al (2004)^74^	375	China	Non-health care (community)	SARS	Confirmed	Yes	********
Yin et al (2004)^75^	257	China	Health care	SARS	Confirmed	Yes	******
Yu et al (2005)^76^	74	China	Health care	SARS	Confirmed	No	*******
Yu et al (2007)^77^	124 wards	China	Health care	SARS	Confirmed	Yes	*******

Across studies, mean age was 30–60 years. SARS=severe acute respiratory syndrome. MERS=Middle East respiratory syndrome.

* The Newcastle-Ottawa Scale was used for the risk of bias assessment, with more stars equalling lower risk.

Some direct evidence was available for COVID-19 (64 studies, of which seven were comparative in design),^36^, ^37^, ^40^, ^41^, ^44^, ^52^, ^70^ but most studies reported on SARS (n=55) or MERS (n=25; appendix pp 6–12). Of the 44 comparative studies, 40 included WHO-defined confirmed cases, one included both confirmed and probable cases, and the remaining three studies included probable cases. There was no effect-modification by case-definition (distance p_interaction_=0·41; mask p_interaction_=0·46; all cases for eye protection were confirmed). Most studies reported on bundled interventions, including different components of PPE and distancing, which was usually addressed by statistical adjustment. The included studies all occurred during recurrent or novel outbreak settings of COVID-19, SARS, or MERS.

Risk of bias was generally low-to-moderate after considering the observational designs (table 1), but both within studies and across studies the overall findings were similar between adjusted and unadjusted estimates. We did not detect strong evidence of publication bias in the body of evidence for any intervention (appendix pp 15–18). As we did not use case series data to inform estimates of effect of each intervention, we did not systematically rate risk of bias of these data. Therefore, we report further only those studies with comparative data.

**Figure 2:**
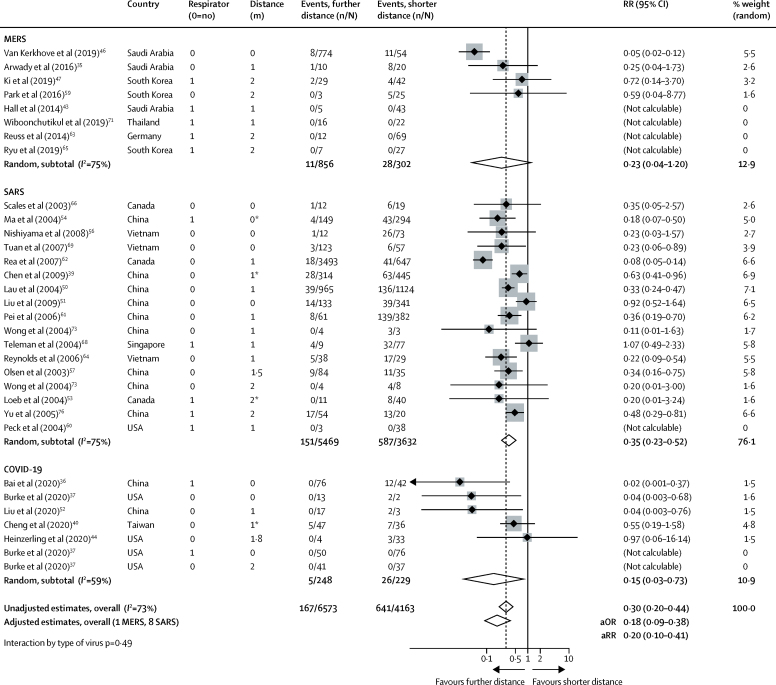
Forest plot showing the association of COVID-19, SARS, or MERS exposure proximity with infection SARS=severe acute respiratory syndrome. MERS=Middle East respiratory syndrome. RR=relative risk. aOR=adjusted odds ratio. aRR=adjusted relative risk. *Estimated values; sensitivity analyses excluding these values did not meaningfully alter findings.

**Figure 3:**
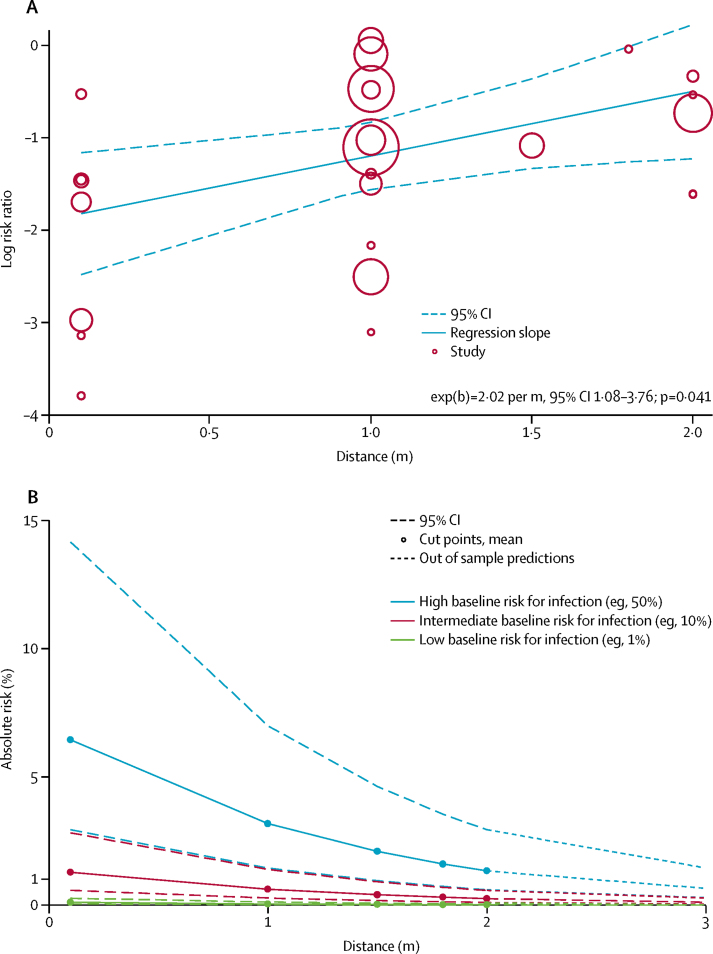
Change in relative risk with increasing distance and absolute risk with increasing distance Meta-regression of change in relative risk with increasing distance from an infected individual (A). Absolute risk of transmission from an individual infected with SARS-CoV-2, SARS-CoV, or MERS-CoV with varying baseline risk and increasing distance (B). SARS-CoV-2=severe acute respiratory syndrome coronavirus 2. SARS-CoV=severe acute respiratory syndrome coronavirus. MERS-CoV=Middle East respiratory syndrome coronavirus.

Across 29 unadjusted and nine adjusted studies,^35^, ^36^, ^37^, ^39^, ^40^, ^43^, ^44^, ^46^, ^47^, ^50^, ^51^, ^52^, ^53^, ^54^, ^56^, ^57^, ^59^, ^60^, ^61^, ^62^, ^63^, ^64^, ^65^, ^66^, ^68^, ^69^, ^71^, ^73^, ^76^ a strong association was found of proximity of the exposed individual with the risk of infection (unadjusted n=10 736, RR 0·30, 95% CI 0·20 to 0·44; adjusted n=7782, aOR 0·18, 95% CI 0·09 to 0·38; absolute risk [AR] 12·8% with shorter distance *vs* 2·6% with further distance, risk difference [RD] −10·2%, 95% CI −11·5 to −7·5; moderate certainty; figure 2; table 2; appendix p 16). Although there were six studies on COVID-19, the association was seen irrespective of causative virus (p_interaction_=0·49), health-care setting versus non-health-care setting (p_interaction_=0·14), and by type of face mask (p_interaction_=0·95; appendix pp 17, 19). However, different studies used different distances for the intervention. By meta-regression, the strength of association was larger with increasing distance (2·02 change in RR per m, 95% CI 1·08 to 3·76; p_interaction_=0·041; moderate credibility subgroup effect; figure 3A; table 2). AR values with increasing distance given different degrees of baseline risk are shown in figure 3B, with potential values at 3 m also shown.

**Table 2:** GRADE summary of findings

	**Studies and participants**	**Relative effect (95% CI)**	**Anticipated absolute effect (95% CI), eg, chance of viral infection or transmission**		**Difference (95% CI)**	Certainty*	**What happens (standardised GRADE terminology)**^29^
			Comparison group	Intervention group			
Physical distance ≥1 m *vs* <1 m	Nine adjusted studies (n=7782); 29 unadjusted studies (n=10 736)	aOR 0·18 (0·09 to 0·38); unadjusted RR 0·30 (95% CI 0·20 to 0·44)	Shorter distance, 12·8%	Further distance, 2·6% (1·3 to 5·3)	−10·2% (−11·5 to −7·5)	Moderate†	A physical distance of more than 1 m probably results in a large reduction in virus infection; for every 1 m further away in distancing, the relative effect might increase 2·02 times
Face mask *vs* no face mask	Ten adjusted studies (n=2647); 29 unadjusted studies (n=10 170)	aOR 0·15 (0·07 to 0·34); unadjusted RR 0·34 (95% CI 0·26 to 0·45)	No face mask, 17·4%	Face mask, 3·1% (1·5 to 6·7)	−14·3% (−15·9 to −10·7)	Low‡	Medical or surgical face masks might result in a large reduction in virus infection; N95 respirators might be associated with a larger reduction in risk compared with surgical or similar masks§
Eye protection (faceshield, goggles) *vs* no eye protection	13 unadjusted studies (n=3713)	Unadjusted RR 0·34 (0·22 to 0·52)¶	No eye protection, 16·0%	Eye protection, 5·5% (3·6 to 8·5)	−10·6% (−12·5 to −7·7)	Low‖	Eye protection might result in a large reduction in virus infection

Table based on GRADE approach.^26^, ^27^, ^28^, ^29^ Population comprised people possibly exposed to individuals infected with SARS-CoV-2, SARS-CoV, or MERS-CoV. Setting was any health-care or non-health-care setting. Outcomes were infection (laboratory-confirmed or probable) and contextual factors. Risk (95% CI) in intervention group is based on assumed risk in comparison group and relative effect (95% CI) of the intervention. All studies were non-randomised and evaluated using the Newcastle-Ottawa Scale; some studies had a higher risk of bias than did others but no important difference was noted in sensitivity analyses excluding studies at higher risk of bias; we did not further rate down for risk of bias. Although there was a high *I*^2^ value (which can be exaggerated in non-randomised studies)^21^ and no overlapping CIs, point estimates generally exceeded the thresholds for large effects and we did not rate down for inconsistency. We did not rate down for indirectness for the association between distance and infection because SARS-CoV-2, SARS-CoV, and MERS-CoV all belong to the same family and have each caused epidemics with sufficient similarity; there was also no convincing statistical evidence of effect-modification across viruses; some studies also used bundled interventions but the studies include only those that provide adjusted estimates. aOR=adjusted odds ratio. RR=relative risk. SARS-CoV-2=severe acute respiratory syndrome coronavirus 2. SARS-CoV=severe acute respiratory syndrome coronavirus. MERS-CoV=Middle East respiratory syndrome coronavirus.

* GRADE category of evidence; high certainty (we are very confident that the true effect lies close to that of the estimate of the effect); moderate certainty (we are moderately confident in the effect estimate; the true effect is probably close to the estimate, but it is possibly substantially different); low certainty (our confidence in the effect estimate is limited; the true effect could be substantially different from the estimate of the effect); very low certainty (we have very little confidence in the effect estimate; the true effect is likely to be substantially different from the estimate of effect).

† The effect is very large considering the thresholds set by GRADE, particularly at plausible levels of baseline risk, which also mitigated concerns about risk of bias; data also suggest a dose–response gradient, with associations increasing from smaller distances to 2 m and beyond, by meta-regression; we did not rate up for this domain alone but it further supports the decision to rate up in combination with the large effects.

‡ The effect was very large, and the certainty of evidence could be rated up, but we made a conservative decision not to because of some inconsistency and risk of bias; hence, although the effect is qualitatively highly certain, the precise quantitative effect is low certainty.

§ In a subgroup analysis comparing N95 respirators with surgical or similar masks (eg, 12–16-layer cotton), the association was more pronounced in the N95 group (aOR 0·04, 95% CI 0·004–0·30) compared with other masks (0·33, 0·17–0·61; p_interaction_=0·090); there was also support for effect-modification by formal analysis of subgroup credibility.

¶ Two studies^54^, ^75^ provided adjusted estimates with n=295 in the eye protection group and n=406 in the group not wearing eye protection; results were similar to the unadjusted estimate (aOR 0·22, 95% CI 0·12–0·39).

‖ The effect is large considering the thresholds set by GRADE assuming that ORs translate into similar magnitudes of RR estimates; this mitigates concerns about risk of bias, but we conservatively decided not to rate up for large or very large effects.

**Figure 4:**
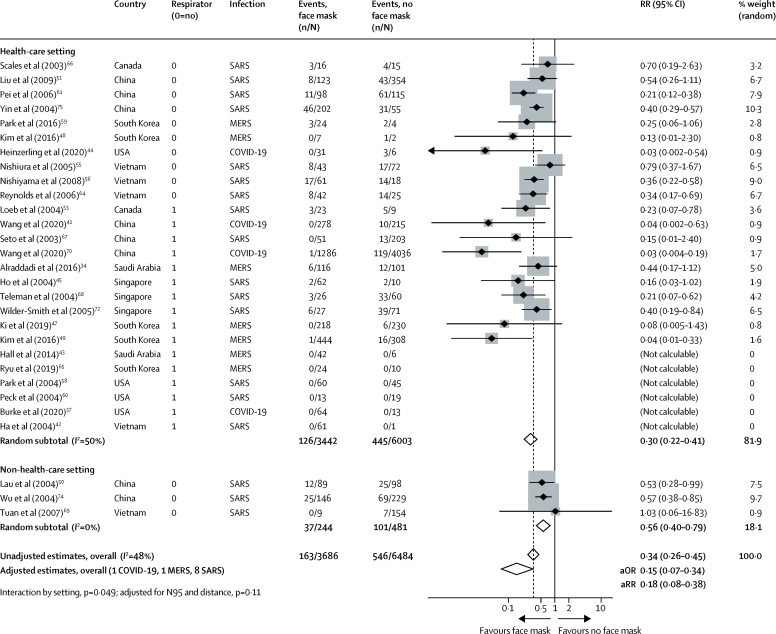
Forest plot showing unadjusted estimates for the association of face mask use with viral infection causing COVID-19, SARS, or MERS SARS=severe acute respiratory syndrome. MERS=Middle East respiratory syndrome. RR=relative risk. aOR=adjusted odds ratio. aRR=adjusted relative risk.

**Figure 5:**
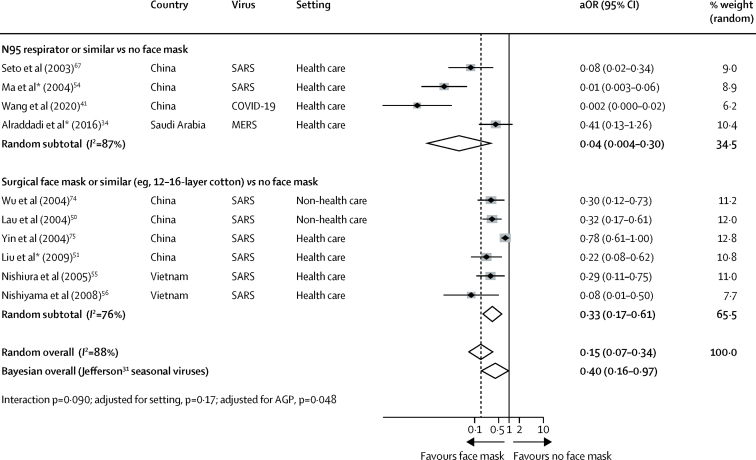
Forest plot showing adjusted estimates for the association of face mask use with viral infection causing COVID-19, SARS, or MERS SARS=severe acute respiratory syndrome. MERS=Middle East respiratory syndrome. RR=relative risk. aOR=adjusted odds ratio. AGP=aerosol-generating procedures. *Studies clearly reporting AGP.

Across 29 unadjusted studies and ten adjusted studies,^34^, ^37^, ^41^, ^42^, ^43^, ^44^, ^45^, ^47^, ^48^, ^49^, ^50^, ^51^, ^53^, ^54^, ^55^, ^56^, ^58^, ^59^, ^60^, ^61^, ^64^, ^65^, ^66^, ^67^, ^68^, ^69^, ^70^, ^72^, ^74^, ^75^ the use of both N95 or similar respirators or face masks (eg, disposable surgical masks or similar reusable 12–16-layer cotton masks) by those exposed to infected individuals was associated with a large reduction in risk of infection (unadjusted n=10 170, RR 0·34, 95% CI 0·26 to 0·45; adjusted studies n=2647, aOR 0·15, 95% CI 0·07 to 0·34; AR 3·1% with face mask *vs* 17·4% with no face mask, RD −14·3%, 95% CI −15·9 to −10·7; low certainty; figure 4; table 2; appendix pp 16, 18) with stronger associations in health-care settings (RR 0·30, 95% CI 0·22 to 0·41) compared with non-health-care settings (RR 0·56, 95% CI 0·40 to 0·79; p_interaction_=0·049; low-to-moderate credibility for subgroup effect; figure 4; appendix p 19). When differential N95 or similar respirator use, which was more frequent in health-care settings than in non-health-care settings, was adjusted for the possibility that face masks were less effective in non-health-care settings, the subgroup effect was slightly less credible (p_interaction_=0·11, adjusted for differential respirator use; figure 4). Indeed, the association with protection from infection was more pronounced with N95 or similar respirators (aOR 0·04, 95% CI 0·004 to 0·30) compared with other masks (aOR 0·33, 95% CI 0·17 to 0·61; p_interaction_=0·090; moderate credibility subgroup effect; figure 5). The interaction was also seen when additionally adjusting for three studies that clearly reported aerosol-generating procedures (p_interaction_=0·048; figure 5). Supportive evidence for this interaction was also seen in within-study comparisons (eg, N95 had a stronger protective association compared with surgical masks or 12–16-layer cotton masks); both N95 and surgical masks also had a stronger association with protection versus single-layer masks.^38^, ^39^, ^51^, ^53^, ^54^, ^61^, ^66^, ^67^, ^75^

We did a sensitivity analysis to test the robustness of our findings and to integrate all available information on face mask treatment effects for protection from COVID-19. We reconsidered our findings using random-effects Bayesian meta-analysis. Although non-informative priors showed similar results to frequentist approaches (aOR 0·16, 95% CrI 0·04–0·40), even using informative priors from the most recent meta-analysis on the effectiveness of masks versus no masks to prevent influenza-like illness (RR 0·93, 95% CI 0·83–1·05)^31^ yielded a significant association with protection from COVID-19 (aOR 0·40, 95% CrI 0·16–0·97; posterior probability for RR <1, 98%). Minimally informing (25% influence with or without four-fold smaller mean effect size) the most recent and rigorous meta-analysis of the effectiveness of N95 respirators versus medical masks in randomised trials (OR 0·76, 95% CI 0·54–1·06)^13^ with the effect-modification seen in this meta-analysis on COVID-19 (ratio of aORs 0·14, 95% CI 0·02–1·05) continued to support a stronger association of protection from COVID-19, SARS, or MERS with N95 or similar respirators versus other face masks (posterior probability for RR <1, 100% and 95%, respectively).

**Figure 6:**
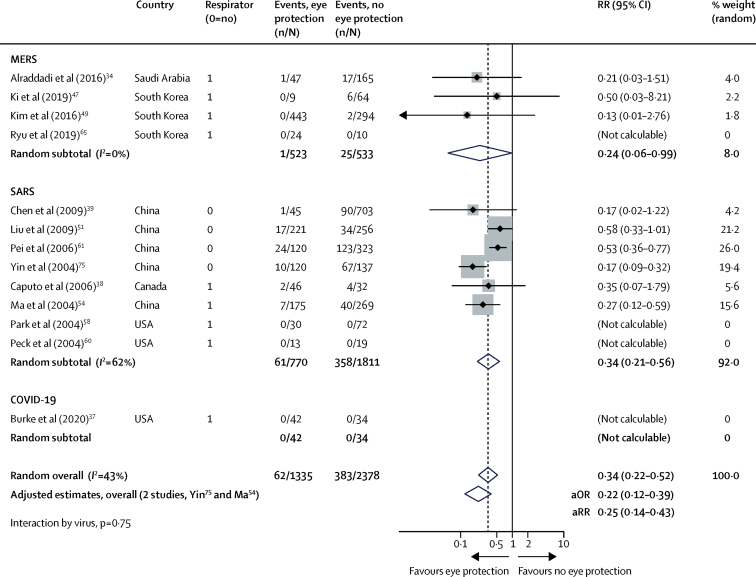
Forest plot showing the association of eye protection with risk of COVID-19, SARS, or MERS transmission Forest plot shows unadjusted estimates. SARS=severe acute respiratory syndrome. MERS=Middle East respiratory syndrome. RR=relative risk. aOR=adjusted odds ratio. aRR=adjusted relative risk.

In 13 unadjusted studies and two adjusted studies,^34^, ^37^, ^38^, ^39^, ^47^, ^49^, ^51^, ^54^, ^58^, ^60^, ^61^, ^65^, ^75^ eye protection was associated with lower risk of infection (unadjusted n=3713, RR 0·34, 95% CI 0·22 to 0·52; AR 5·5% with eye protection *vs* 16·0% with no eye protection, RD −10·6%, 95% CI −12·5 to −7·7; adjusted n=701, aOR 0·22, 95% CI 0·12 to 0·39; low certainty; figure 6; table 2; appendix pp 16–17).

Across 24 studies in health-care and non-health-care settings during the current pandemic of COVID-19, previous epidemics of SARS and MERS, or in general use, looking at contextual factors to consider in recommendations, most stakeholders found physical distancing and use of face masks and eye protection acceptable, feasible, and reassuring (appendix pp 20–22). However, challenges included frequent discomfort, high resource use linked with potentially decreased equity, less clear communication, and perceived reduced empathy of care providers by those they were caring for.

## Discussion

The findings of this systematic review of 172 studies (44 comparative studies; n=25 697 patients) on COVID-19, SARS, and MERS provide the best available evidence that current policies of at least 1 m physical distancing are associated with a large reduction in infection, and distances of 2 m might be more effective. These data also suggest that wearing face masks protects people (both health-care workers and the general public) against infection by these coronaviruses, and that eye protection could confer additional benefit. However, none of these interventions afforded complete protection from infection, and their optimum role might need risk assessment and several contextual considerations. No randomised trials were identified for these interventions in COVID-19, SARS, or MERS.

Previous reviews are limited in that they either have not provided any evidence from COVID-19 or did not use direct evidence from other related emerging epidemic betacoronaviruses (eg, SARS and MERS) to inform the effects of interventions to curtail the current COVID-19 pandemic.^13^, ^19^, ^31^, ^78^ Previous data from randomised trials are mainly for common respiratory viruses such as seasonal influenza, with a systematic review concluding low certainty of evidence for extrapolating these findings to COVID-19.^13^ Further, previous syntheses of available randomised controlled trials have not accounted for cluster effects in analyses, leading to substantial imprecision in treatment effect estimates. In between-study and within-study comparisons, we noted a larger effect of N95 or similar respirators compared with other masks. This finding is inconsistent with conclusions of a review of four randomised trials,^13^ in which low certainty of evidence for no larger effect was suggested. However, in that review, the CIs were wide so a meaningful protective effect could not be excluded. We harmonised these findings with Bayesian approaches, using indirect data from randomised trials to inform posterior estimates. Despite this step, our findings continued to support the ideas not only that masks in general are associated with a large reduction in risk of infection from SARS-CoV-2, SARS-CoV, and MERS-CoV but also that N95 or similar respirators might be associated with a larger degree of protection from viral infection than disposable medical masks or reusable multilayer (12–16-layer) cotton masks. Nevertheless, in view of the limitations of these data, we did not rate the certainty of effect as high.^21^ Our findings accord with those of a cluster randomised trial showing a potential benefit of continuous N95 respirator use over medical masks against seasonal viral infections.^79^ Further high-quality research, including randomised trials of the optimum physical distance and the effectiveness of different types of masks in the general population and for health-care workers' protection, is urgently needed. Two trials are registered to better inform the optimum use of face masks for COVID-19 (NCT04296643 [n=576] and NCT04337541 [n=6000]). Until such data are available, our findings represent the current best estimates to inform face mask use to reduce infection from COVID-19. We recognise that there are strong, perhaps opposing, sentiments about policy making during outbreaks. In one viewpoint, the 2007 SARS Commission report stated:

“...recognize, as an aspect of health worker safety, the precautionary principle that reasonable action to reduce risk, such as the use of a fitted N95 respirator, need not await scientific certainty”.^80^

“...if we do not learn from SARS and we do not make the government fix the problems that remain, we will pay a terrible price in the next pandemic”.^81^

A counter viewpoint is that the scientific uncertainty and contextual considerations require a more nuanced approach. Although challenging, policy makers must carefully consider these two viewpoints along with our findings.

We found evidence of moderate certainty that current policies of at least 1 m physical distancing are probably associated with a large reduction in infection, and that distances of 2 m might be more effective, as implemented in some countries. We also provide estimates for 3 m. The main benefit of physical distancing measures is to prevent onward transmission and, thereby, reduce the adverse outcomes of SARS-CoV-2 infection. Hence, the results of our current review support the implementation of a policy of physical distancing of at least 1 m and, if feasible, 2 m or more. Our findings also provide robust estimates to inform models and contact tracing used to plan and strategise for pandemic response efforts at multiple levels.

The use of face masks was protective for both health-care workers and people in the community exposed to infection, with both the frequentist and Bayesian analyses lending support to face mask use irrespective of setting. Our unadjusted analyses might, at first impression, suggest use of face masks in the community setting to be less effective than in the health-care setting, but after accounting for differential N95 respirator use between health-care and non-health-care settings, we did not detect any striking differences in effectiveness of face mask use between settings. The credibility of effect-modification across settings was, therefore, low. Wearing face masks was also acceptable and feasible. Policy makers at all levels should, therefore, strive to address equity implications for groups with currently limited access to face masks and eye protection. One concern is that face mask use en masse could divert supplies from people at highest risk for infection.^10^ Health-care workers are increasingly being asked to ration and reuse PPE,^82^, ^83^ leading to calls for government-directed repurposing of manufacturing capacity to overcome mask shortages^84^ and finding solutions for mask use by the general public.^84^ In this respect, some of the masks studied in our review were reusable 12–16-layer cotton or gauze masks.^51^, ^54^, ^61^, ^75^ At the moment, although there is consensus that SARS-CoV-2 mainly spreads through large droplets and contact, debate continues about the role of aerosol,^2^, ^3^, ^4^, ^5^, ^6^, ^7^, ^8^, ^85^, ^86^ but our meta-analysis provides evidence (albeit of low certainty) that respirators might have a stronger protective effect than surgical masks. Biological plausibility would be supported by data for aerosolised SARS-CoV-2^5^, ^6^, ^7^, ^8^ and preclinical data showing seasonal coronavirus RNA detection in fine aerosols during tidal breathing,^87^ albeit, RNA detection does not necessarily imply replication and infection-competent virus. Nevertheless, our findings suggest it plausible that even in the absence of aerosolisation, respirators might be simply more effective than masks at preventing infection. At present, there is no data to support viable virus in the air outside of aerosol generating procedures from available hospital studies. Other factors such as super-spreading events, the subtype of health-care setting (eg, emergency room, intensive care unit, medical wards, dialysis centre), if aerosolising procedures are done, and environmental factors such as ventilation, might all affect the degree of protection afforded by personal protection strategies, but we did not identify robust data to inform these aspects.

Strengths of our review include adherence to full systematic review methods, which included artificial intelligence-supported dual screening of titles and abstracts, full-text evaluation, assessment of risk of bias, and no limitation by language. We included patients infected with SARS-CoV-2, SARS-CoV, or MERS-CoV and searched relevant data up to May 3, 2020. We followed the GRADE approach^16^ to rate the certainty of evidence. Finally, we identified and appraise a large body of published work from China, from which much evidence emerged before the pandemic spread to other global regions.

The primary limitation of our study is that all studies were non-randomised, not always fully adjusted, and might suffer from recall and measurement bias (eg, direct contact in some studies might not be measuring near distance). However, unadjusted, adjusted, frequentist, and Bayesian meta-analyses all supported the main findings, and large or very large effects were recorded. Nevertheless, we are cautious not to be overly certain in the precise quantitative estimates of effects, although the qualitative effect and direction is probably of high certainty. Many studies did not provide information on precise distances, and direct contact was equated to 0 m distance; none of the eligible studies quantitatively evaluated whether distances of more than 2 m were more effective, although our meta-regression provides potential predictions for estimates of risk. Few studies assessed the effect of interventions in non-health-care settings, and they primarily evaluated mask use in households or contacts of cases, although beneficial associations were seen across settings. Furthermore, most evidence was from studies that reported on SARS and MERS (n=6674 patients with COVID-19, of 25 697 total), but data from these previous epidemics provide the most direct information for COVID-19 currently. We did not specifically assess the effect of duration of exposure on risk for transmission, although whether or not this variable was judged a risk factor considerably varied across studies, from any duration to a minimum of 1 h. Because of inconsistent reporting, information is limited about whether aerosol-generating procedures were in place in studies using respirators, and whether masks worn by infected patients might alter the effectiveness of each intervention, although the stronger association with N95 or similar respirators over other masks persisted when adjusting for studies reporting aerosol-generating medical procedures. These factors might account for some of the residual statistical heterogeneity seen for some outcomes, albeit *I*^2^ is commonly inflated in meta-analyses of observational data,^21^, ^22^ and nevertheless the effects seen were large and probably clinically important in all adjusted studies.

Our comprehensive systematic review provides the best available information on three simple and common interventions to combat the immediate threat of COVID-19, while new evidence on pharmacological treatments, vaccines, and other personal protective strategies is being generated. Physical distancing of at least 1 m is strongly associated with protection, but distances of up to 2 m might be more effective. Although direct evidence is limited, the optimum use of face masks, in particular N95 or similar respirators in health-care settings and 12–16-layer cotton or surgical masks in the community, could depend on contextual factors; action is needed at all levels to address the paucity of better evidence. Eye protection might provide additional benefits. Globally collaborative and well conducted studies, including randomised trials, of different personal protective strategies are needed regardless of the challenges, but this systematic appraisal of currently best available evidence could be considered to inform interim guidance.
