# Supplementary material for: Physical distancing, face masks, and eye protection to prevent person-to-person transmission of SARS-CoV-2 and COVID-19: a systematic review and meta-analysis
Source: Lancet. 2020 Jun 27;395(10242):1973–87. doi: 10.1016/S0140-6736(20)31142-9 (PMC7263814; doi:10.1016/S0140-6736(20)31142-9)
Supplement: Supplementary appendix [file mmc1.pdf]

THE LANCET

Supplementary appendix

This appendix formed part of the original submission and has been peer reviewed. We post it as supplied by the authors.

Supplement to: Chu DK, Akl EA, Duda S, et al. Physical distancing, face masks, and eye protection to prevent person-to-person transmission of SARS-CoV-2 and COVID-19: a systematic review and meta-analysis. *Lancet* 2020; published online June 1. https://doi.org/10.1016/S0140-6736(20)31142-9.

Supplementary material

Physical distancing, face masks, and eye protection to prevent person-person SARS-CoV2 and COVID-19 transmission: A systematic review and meta-analysis

The COVID-19 Systematic Urgent Review Group Effort (SURGE) Study Group

**Full name spelling**	**Degree**	**Department**	**Affiliations**
Derek K. Chu	MD, PhD	Medicine; Health Research Methods, Evidence & Impact	McMaster University;<br>The Research Institute of St. Joe's Hamilton
Elie Akl	MD, MPH, PhD	Internal Medicine	American University of Beirut
Amena El-Harakeh	MPH	Clinical Research Institute	American University of Beirut
Antonio Bognanni	MD	Health Research Methods, Evidence & Impact	McMaster University
Tamara Lotfi	MD,MPH	Health Research Methods, Evidence & Impact	McMaster University
Mark Loeb	MD, MSc	Pathology and Molecular Medicine and Health Research Methods, Evidence, and Impact	McMaster University, Canada
Anisa Hajizadeh	BHSc	Health Research Methods, Evidence, and Impact	McMaster University, Canada
Anna Bak	PharmD	None	Evidence Prime, Krakow
Ariel Izcovich	MD, PhD	Department of Internal Medicine	German Hospital of Buenos Aires
Carlos A. Cuello-Garcia	MD, PhD	Health Research Methods Evidence and Impact	McMaster University
Chen Chen	MM	The Fourth Clinical Medical College	Guangzhou University of Chinese Medicine
David James Harris	MD, MHSc, DTMH	Medicine	University of British Columbia
Ewa Borowiack	MSc	None	Evidence Prime, Krakow
Fatimah Chamseddine	MD	Clinical Research Institute	American University of Beirut
Finn Schünemann	MD	None	Germany
Gian Paolo Morgano	MSc	Health Research Methods, Evidence, and Impact	McMaster University, Canada
Giovanna Elsa Ute Muti Schünemann	Cand. Med.	Medical School	Vita Salute San Raffaele University
Guang Chen	MD, PhD	Dongzhimen Hospital	Beijing University of Chinese Medicine
Hong Zhao	PhD	Institute of acupuncture and moxibustion	China academy of Chinese Medical Sciences
Ignacio Neumann	MD, MSc, PhD	Department of Internal Medicine	Pontificia Universidad Católica de Chile
Jeffrey Chan	MD, CCFP-EM	Department of Emergency Medicine	Southlake Regional Health Centre

Joanne Khabsa	BSPharm, MPH	Clinical Research Institute	American University of Beirut
Layal Hneiny	MPH, MLIS	University Libraries, Saab Medical Library	American University of Beirut
Leila Harrison	MPH	Health Research Methods, Evidence, and Impact	McMaster University, Canada
Maureen Smith	B.A. (Honours), Dip. Ed., M.Ed.		Chair, Cochrane Consumer Executive
Nesrine Rizk	MD	Department of Internal Medicine	American University of Beirut
Paolo Giorgi Rossi	PhD	Epidemiology Unit	Azienda USL - IRCCS di Reggio Emilia
Pierre AbiHanna	MD, MPH	Internal Medicine	Rafik Hariri University Hospital
Rayane El-Khoury	MPH	Clinical Research Institute	American University of Beirut
Rosa Stalteri	HBSc	Health Research Methods, Evidence & Impact	McMaster University
Tejan Baldeh	MPH	Health Research Methods, Evidence & Impact	McMaster University
Thomas Piggott	MD, MSc	Health Research Methods, Evidence & Impact	McMaster University
Yuan Zhang	PhD, MSc	Health Research Methods, Evidence & Impact	McMaster University
Zahra Saad	MSc	Global Evidence Synthesis Initiative (GESI) Secretariat	American University of Beirut
Assem Khamis	MD, MPH	Hull York Medical School	University of Hull
Marge Reinap	MA Economics	The London School of Hygiene & Tropical Medicine	The London School of Hygiene & Tropical Medicine
Stephanie Duda	MSc	Health Research Methods, Evidence & Impact	McMaster University
Karla Solo	BMSc, MSc	Department of Health Research Methods, Evidence, and Impact	McMaster University
Sally Yaacoub	BSPharm, MPH	Clinical Research Institute	American University of Beirut
Holger Schünemann	MD, PhD	Health Research Methods, Evidence & Impact; Medicine; WHO Collaborating Center for Infectious Diseases, Research Methods and Recommendations; Michael G DeGroote Cochrane Canada Centre; GRADE Canada Centre	McMaster University

Correspondence

Holger J. Schünemann, MD, PhD
WHO Collaborating Center for Infectious Diseases, Research Methods and Recommendations; Michael G DeGroote Cochrane Canada and McMaster GRADE Centres; McMaster University, HSC-2C, 1280 Main St West; Hamilton, ON L8N 3Z5, Canada; E-mail: schuneh@mcmaster.ca

Contents:

1) Search strategies
2) Characteristics of included studies
3) Risk of bias assessments
4) Funnel plots
5) Evidence profiles
6) Forest plots for additional analyses
7) Sensitivity analyses
8) Credibility assessment of potential effect modifiers
9) Summary of contextual factor data
10) PROSPERO protocol registration
11) PRISMA checklist, MOOSE checklists, References

Appendix 1. Search strategies for the different databases ran on March 26, 2020. Preprint and coronavirus searches were run daily until May 3, 2020.

We developed the search strategy with the assistance of an information specialist experienced with systematic reviews (LH). Two information specialists (Ms. Neera Bhatnagar and Ms. Aida Farha) peer reviewed the search strategy. Other members of the team, particularly the content experts provided feedback to the search strategy. The strategies combined medical subject headings (MeSH) and keywords for the two following concepts: COVID-19 and personal protection by any of physical distancing, masks, or eye protection. PubMed search terms were informed by the Biomedical Information of the Dutch Library Association specialists curated search blocks at https://blocks.bmi-online.nl/catalog/397.

Medline (OVID)

Ovid MEDLINE(R) and Epub Ahead of Print, In-Process & Other Non-Indexed Citations and Daily 1946 to March 26, 2020

1 (pneumonia/ or pneumonia, viral/ or exp Viruses/) and (exp Disease Outbreaks/ or exp Epidemiology/ or Epidemiology.fs.) (104129)

2 coronaviridae/ or exp coronavirus/ or exp Coronavirus Infections/ or exp Betacoronavirus/ (15998)

3 (Betacoronavirus or Beta-coronavirus or Coronavirus* or COVID).mp. (14380)

4 1 or 2 or 3 (121096)

5 limit 4 to ez="20191101-20200325" (1524)

6 (("2019" adj (novel or new) adj corona*) or ("2019" adj (CoV or nCoV)) or (coronavirus adj (disease adj "2019")) or COVID19 or COVID-19 or ((Novel or New) adj Corona*) or SARS2 or SARS-CoV-2 or (SARS adj2 (coronaviridae or coronavirus)) or ((sars or Coronavirus) adj "2") or nCov or 2019ncov).mp. (4983)

7 5 or 6 (5522)

8 (Mask? or facemask? or face-mask? or ppe or (body adj substance* adj isolati*) or bsi or (infect* adj prevent* adj control*) or ipc or N95 or ffp or ffp1 or ffp3 or ffp2 or (filter* adj face adj piece) or ((face or respiratory or eye) adj2 (shield or equipment? or protect* or cover*)) or ((airborne or air-borne or droplet*) adj precau*) or N99 or N97 or respirator? or goggle? or ((patient? or person* or individual?) adj1 isolat*) or distanc* or space or spacing or separation or (aerosol adj generat* adj procedur*) or ((safety or protective) adj (supply or supplies or device* or equipment? or material* or measure* or gear?)) or (safely adj1 equipped) or meter? or metre? or foot or feet or (non-pharm* adj intervention*) or ((physical or person*) adj (intervention* or barrier? or protect*)) or transmission* or contamination? or shedding? or fomite? or gap? or ((head or face) adj cover?) or (protective adj clothing?)).mp. or masks/ or protective devices/ or personal protective equipment/ or respiratory protective devices/ or Eye Protective Devices/ (2489045)

9 7 and 8 (3314)

PubMed

Search Query

#7 Search ((((#4 OR #5))) AND (((mask[tw] OR masks[tw] OR facemask[tw] OR facemasks[tw] OR face-mask[tw] OR face-masks[tw] OR PPE[tw] OR body substance isolation*[tw] OR bsi[tw] OR infection prevention control*[tw] OR ipc[tw] OR N95[tw] OR ffp[tw] OR ffp1[tw] OR ffp3[tw] OR ffp2[tw] OR N97[tw] OR N99[tw] OR physical barrier*[tw] OR physical intervention*[tw] OR physical protection*[tw] OR personal protection*[tw] OR person protection*[tw] OR transmission[tw] OR transmissions[tw] OR contamination[tw] OR contaminations[tw] OR shedding[tw] OR fomite[tw] OR gap[tw] OR gaps[tw] OR non-pharm intervention*[tw] OR non-pharmaceutical intervention*[tw] OR distancing[tw] OR space [tw] OR distances[tw] OR spacing[tw] OR separation[tw] OR respirator[tw] OR respirators[tw] OR aerosol-generating procedure*[tw] OR patient isolation*[tw] OR patient isolator*[tw] OR person isolation[tw] OR person isolator*[tw] OR individual isolation[tw] OR individual isolator*[tw] OR filtering face piece[tw] OR filtering face piece*[tw] OR [tw] OR face protection*[tw] OR face shield*[tw] OR face protective device*[tw] OR face protective gear*[tw] OR eye protection*[tw] OR eye shield*[tw] OR eye protective device*[tw] OR eye protective gear*[tw] OR airborne precaution*[tw] OR droplet precautions*[tw] OR safety supply*[tw] OR safety supplies*[tw] OR safety device*[tw] OR safety equipment*[tw] OR safety measure*[tw] OR safety gear*[tw] OR protective supply*[tw] OR protective supplies*[tw] OR protective device*[tw] OR protective equipment*[tw] OR protective measure*[tw] OR protective gear*[tw] OR person isolation[tw] OR personal isolation[tw] OR individual isolation[tw] OR respirator[tw] OR respirators[tw] OR respiratory protection*[tw] OR respiratory protective device*[tw] OR respiratory protective supply*[tw] OR respiratory protective supplies*[tw] OR respiratory protective equipment*[tw] OR respiratory protective gear*[tw] OR safely equipped*[tw] OR meter[tw] OR metre[tw] OR foot[tw] OR feet[tw] OR meters[tw] OR metres[tw] OR head cover*[tw] OR face cover*[tw] OR eye cover*[tw] OR goggle*[tw] OR protective clothing*[tw])) OR (((("Masks"[Mesh:NoExp]) OR "Protective Devices"[Mesh]) OR "Personal Protective Equipment"[Mesh:NoExp]) OR "Respiratory Protective Devices"[Mesh:NoExp] OR "Eye Protective Devices"[Mesh:NoExp])))

#6 Search ((#4 OR #5))

#5 Search (((2019-novel-corona* OR 2019-new-corona* OR novel-corona* OR new-corona* OR 2019-Cov OR 2019-nCov OR nCov OR coronavirus disease-2019 OR SARS2 OR SARS-2 OR SARS-CoV-2 OR sars cORona* OR COROnavirus-2 OR 2019ncov)))

#4 Search ((((#1 OR #2 OR #3) AND 2019/11:2020/03 [crdt])))

#3 Search (((BetacORonavirus[tw] OR Beta-cORonavirus[tw] OR corona[tw] OR corona'[tw] OR corona's[tw] OR OR coronaviral[tw] OR coronavirdae[tw] OR coronavirida[tw] OR coronaviridae[tw] OR coronaviridea[tw] OR coronaviridiae[tw] OR coronavirinae[tw] OR coronavirion[tw] OR coronavirions[tw] OR coronavirologists[tw] OR coronavirology[tw] OR coronaviroses[tw] OR coronavirous[tw] OR coronavirues[tw] OR coronavirus[tw] OR coronavirus'[tw] OR coronavirus's[tw] OR

coronaviruscpe[tw] OR coronaviruse[tw] OR coronaviruses[tw] OR coronaviruses'[tw] OR coronaviruslike[tw] OR coronaviser[tw] OR coronaviurs[tw] OR coronaviuses[tw] OR coronavrius[tw] OR coronavvirus[tw] OR COVID[tw])))
#2 Search ((((pneumonia[Mesh:noexp] OR pneumonia, viral[Mesh:noexp] OR Viruses[Mesh]) and ("Disease Outbreaks"[Mesh] OR Epidemiology[Mesh] OR Epidemiology [Mesh subject heading]))))
#1 Search (((cORonaviridae[Mesh:noexp] OR cORonavirus[Mesh] OR "Coronavirus Infections"[Mesh] OR BetacORonavirus[Mesh])))

EMBASE

No.	Query
#18	#7 AND #17
#17	#8 OR #9 OR #10 OR #11 OR #12 OR #13 OR #14 OR #15 OR #16
#16	'mask'/de OR 'protective equipment'/de OR 'respiratory protection'/de OR 'eye mask'/de
#15	meter$:ti,ab,kw OR metre$:ti,ab,kw OR foot:ti,ab,kw OR feet:ti,ab,kw OR (('non pharm*' NEXT/0 intervention*):ti,ab,kw) OR (((physical OR person*) NEXT/0 (intervention* OR barrier$ OR protect*)):ti,ab,kw) OR transmission*:ti,ab,kw OR contamination$:ti,ab,kw OR shedding$:ti,ab,kw OR fomite$:ti,ab,kw OR gap$:ti,ab,kw
#14	((filter* NEXT/0 face NEXT/0 piece):ti,ab,kw) OR (((face OR respiratory OR eye) NEAR/2 (shield OR equipment$ OR protect* OR cover$)):ti,ab,kw)
#13	((safety OR protective) NEXT/0 (supply OR supplies OR device* OR equipment? OR material* OR measure* OR gear$)) AND ti,ab,kw OR ((safely NEAR/1 equipped):ti,ab,kw)
#12	distanc*:ti,ab,kw OR space:ti,ab,kw OR spacing:ti,ab,kw OR separation:ti,ab,kw OR ((aerosol NEXT/0 generat* NEXT/0 procedur*):ti,ab,kw)
#11	(((airborne OR 'air borne' OR droplet$) NEXT/0 precau*):ti,ab,kw) OR n99:ti,ab,kw OR n97:ti,ab,kw OR goggle$:ti,ab,kw OR respirator$:ti,ab,kw OR (((patient$ OR person* OR individual$) NEXT/0 isolat*):ti,ab,kw)
#10	((filter* NEXT/0 face NEXT/0 piece):ti,ab,kw) OR (((face OR respiratory) NEAR/2 (shield OR equipment$ OR protect*)):ti,ab,kw)
#9	'ppe':ti,ab,kw OR ((body NEXT/0 substance$ NEXT/0 isolati*):ti,ab,kw) OR bsi:ti,ab,kw OR ((infect* NEXT/0 prevent* NEXT/0 control*):ti,ab,kw) OR ipc:ti,ab,kw OR n95:ti,ab,kw OR ffp:ti,ab,kw OR ffp1:ti,ab,kw OR ffp3:ti,ab,kw OR ffp2:ti,ab,kw
#8	mask$:ti,ab,kw OR facemask$:ti,ab,kw OR 'face mask':ti,ab,kw
#7	#5 OR #6
#6	((2019 NEXT/0 novel):ti,ab,kw) OR ((2019 NEXT/0 cov):ti,ab,kw) OR ((coronavirus NEXT/0 disease NEXT/0 2019):ti,ab,kw) OR covid19:ti,ab,kw OR 'covid 19':ti,ab,kw OR (((novel OR new) NEXT/0 corona*):ti,ab,kw) OR sars2:ti,ab,kw OR 'sars cov 2':ti,ab,kw OR ((sars NEAR/2 coronaviridae):ti,ab,kw) OR coronavirus:ti,ab,kw OR sars:ti,ab,kw OR ((coronavirus NEXT/0 '2'):ti,ab,kw) OR ncov:ti,ab,kw OR 2019ncov:ti,ab,kw
#5	#4 AND [1-11-2019]/sd
#4	#1 OR #2 OR #3
#3	betacoronavirus:ti,ab,kw OR 'beta coronavirus':ti,ab,kw OR coronavirus*:ti,ab,kw OR covid:ti,ab,kw
#2	'coronaviridae'/exp OR 'coronavirus infection'/exp OR 'betacoronavirus'/exp
#1	('pneumonia'/de OR 'virus pneumonia'/de OR 'virus'/exp) AND ('epidemic'/exp OR 'epidemiology'/exp OR epidemiology:lnk)

CINAHL (OVID)

Cochrane Library

ID	Search	Hits
#1	MeSH descriptor: [Pneumonia, Viral] this term only	51
#2	MeSH descriptor: [Pneumonia] this term only	1976
#3	MeSH descriptor: [Viruses] explode all trees	8746
#4	#1 OR #2 OR #3	10734
#5	MeSH descriptor: [Disease Outbreaks] explode all trees	262
#6	MeSH descriptor: [Epidemiology] explode all trees	37
#7	(Epidemiology):ti,ab,kw	48587
#8	#5 OR #6 OR #7	48682
#9	#4 AND #8	1315
#10	MeSH descriptor: [Coronaviridae] this term only	0
#11	MeSH descriptor: [Coronavirus] explode all trees	11
#12	MeSH descriptor: [Coronavirus Infections] explode all trees	12
#13	MeSH descriptor: [Betacoronavirus] explode all trees	10
#14	(Betacoronavirus or Beta-coronavirus or Coronavirus* or COVID):ti,ab,kw	98
#15	#9 OR #10 OR #11 OR #12 OR #13 OR #14 with Cochrane Library publication date Between Nov 2019 and Mar 2020	44
#16	((2019 NEXT (novel or new) NEXT corona*)):ti,ab,kw	8

#17 (("2019" NEXT (CoV or nCoV)) or (coronavirus NEXT (disease NEXT "2019")) or COVID19 or COVID-19 or ((Novel or New) NEXT Corona*) or SARS2 or SARS-CoV-2 or (SARS NEAR/2 (coronaviridae or coronavirus)) or ((sars or Coronavirus) NEXT "2") or nCov or 2019ncov):ti,ab,kw 118
#18 #15 OR #16 OR #17 145
#19 MeSH descriptor: [Masks] this term only 475
#20 MeSH descriptor: [Protective Devices] this term only 207
#21 MeSH descriptor: [Personal Protective Equipment] this term only 19
#22 MeSH descriptor: [Respiratory Protective Devices] this term only 66
#23 MeSH descriptor: [Eye Protective Devices] this term only 65
#24 (Mask? OR facemask? OR face-mask? OR ppe OR (body NEAR substance* NEAR isolati*) OR bsi OR (infect* NEAR prevent* NEAR control*) OR ipc OR N95 OR ffp OR ffp1 OR ffp3 OR ffp2 OR (filter* NEAR face NEAR piece) OR ((face OR respiratORy OR eye) NEXT/2 (shield OR equipment? OR protect* OR cover*)) OR ((airbORne OR air-bORne OR droplet*) NEAR precau*) OR N99 OR N97 OR respiratOR? OR goggle? OR ((patient? OR person* OR individual?) NEXT/1 isolat*) OR distanc* OR space OR spacing OR separation OR (aerosol NEAR generat* NEAR procedur*) OR ((safety OR protective) NEAR (supply OR supplies OR device* OR equipment? OR material* OR measure* OR gear?)) OR (safely NEAR/1 equipped) OR meter? OR metre? OR foot OR feet OR (non-pharm* NEAR intervention*) OR ((physical OR person*) NEAR (intervention* OR barrier? OR protect*)) OR transmission* OR contamination? OR shedding? OR fomite? OR gap? OR ((head or face) NEXT cover?) OR (protective NEXT clothing?)):ti,ab,kw 161945
#25 #19 OR #20 OR #21 OR #22 OR #23 OR #24 161945
#26 #18 AND #25 43

China National Knowledge Infrastructure (CNK)I 中国知网--topic words searching in Chinese
新型冠状病毒性肺炎，新冠肺炎，新型冠状病毒，冠状病毒感染，冠状病毒肺炎，冠状病毒，COVID-19

Science Chinese Biomedical Literature Database (SinoMed)—field searching in Chinese
("2019冠状病毒"[常用字段:智能] OR "新型冠状病毒"[常用字段:智能] OR "新冠肺炎"[常用字段:智能] OR "2019-nCoV"[常用字段:智能] OR "SARS-CoV-2"[常用字段:智能] OR "Novel coronavirus"[常用字段:智能] OR "nCoV"[常用字段:智能] OR "Emerging Coronaviruses"[常用字段:智能] OR "new coronavirus"[常用字段:智能] OR "COVID-19"[常用字段:智能] OR "coronavirus"[常用字段:智能] AND ("Wuhan"[常用字段] OR "Hubei"[常用字段] OR "China"[常用字段])) AND 2019-2020[日期]

Appendix 2. Characteristics of included studies

Study ID[Reference]	Study Design	Country	Setting	Virus
Alameer 2015(1)	Non-comparative	Saudi Arabia	Healthcare setting	MERS
Alanazi 2018(2)	Non-comparative	Saudi Arabia	Healthcare setting	MERS
Alfaraj 2018(3)	Comparative NRS	Saudi Arabia	Non-healthcare setting	MERS
Alraddadi 2016(4)	Comparative NRS	Saudi Arabia	Healthcare setting	MERS
Al-Tawfiq 2019(5)	Qualitative	Saudi Arabia	Healthcare setting	MERS
Assiri 2013(6)	Non-comparative	Saudi Arabia	Healthcare setting	MERS
Bai 2020(7)	Non-comparative	China	Non-healthcare setting	COVID-19
Bai 2020(8)	Comparative	China	Healthcare setting	COVID-19
Barratt 2019(9)	Qualitative	Australia	Healthcare setting	Other
Baseer 2016(10)	Qualitative	Saudi Arabia	Healthcare setting	MERS
Booth 2005(11)	Mechanistic	Canada	Healthcare setting	SARS
Cai 2020(12)	Contextual factors - qualitative or quantiative	China	Non-healthcare setting	COVID-19
Cao 2020(13)	Non-comparative	China	Non-healthcare setting	COVID-19
Caputo 2006(14)	Comparative NRS	Canada	Healthcare setting	SARS
Chau 2010(15)	Qualitative	China	Healthcare setting	Other
Chen 2004(16)	Non-comparative	Taiwan	Healthcare setting	SARS
Chen 2009(17)	Comparative NRS - Cohort	China	Healthcare setting	SARS
Chen 2020(18)	Contextual factors - qualitative or quantiative	China	Non-healthcare setting	COVID-19
Chen 2020(19)	Non-comparative	China	Non-healthcare setting	COVID-19
Chen 2020(20)	Comparative NRS	China	Non-healthcare setting	COVID-19
Chen 2020(21)	Non-comparative	China	Healthcare setting	COVID-19
Cheng 2020(22)	Non-comparative - mechanistic	China	Healthcare setting	COVID-19
Chia 2005(23)	Qualitative	Singapore	Healthcare setting	SARS
Christian 2004(24)	Non-comparative - Case series	Canada	Healthcare setting	SARS
Chughtai 2015(25)	Qualitative	Vietnam	Healthcare setting	Other

Study ID[Reference]	Study Design	Country	Setting	Virus
Chughtai 2020(26)	Qualitative	Australia	Healthcare setting	Other
Cui 2020(27)	Comparative NRS	China	Non-healthcare setting	COVID-19
Du 2020(28)	Comparative NRS	China	Non-healthcare setting	COVID-19
El Bushra 2016(29)	Non-comparative - Case series	Saudi Arabia	Healthcare setting	MERS
Fan 2020(30)	Comparative NRS - Cohort	China	Healthcare setting	COVID-19
Feng 2020(31)	Non-comparative	China	Non-healthcare setting	COVID-19
Fix 2019(32)	Qualitative	United States of America	Healthcare setting	SARS
Gan 2020(33)	Comparative NRS	China	Non-healthcare setting	COVID-19
Goh 2019(34)	Qualitative	Singapore	Healthcare setting	NA
Gomersall 2006(35)	Non-comparative - Cohort (but all received the intervention)	China	Healthcare setting	SARS
Ha 2004(36)	Comparative NRS - Cohort	Vietnam	Healthcare setting	SARS
Hall 2014(37)	Comparative NRS - Cohort	Saudi Arabia	Healthcare setting	MERS
Hines 2019(38)	Qualitative	United States of America	Healthcare setting	Other
Ho 2003(39)	Non-comparative - Case series	China	Healthcare setting	SARS
Ho 2004(40)	Comparative NRS - Cohort	Singapore	Healthcare setting	SARS
Ho 2012(41)	Qualitative	China	Healthcare setting	Other
Honarbakhsh 2018(42)	Qualitative	Iran	Healthcare setting	Other
Huang 2011(43)	Qualitative	Taiwan	Healthcare setting	Respiratory infectious diseases
Hunter 2016(44)	Non-comparative - Case series	United Arabic Emirates	Healthcare setting	MERS
Huynh 2020(45)	Contextual factors - qualitative or quantiative	Vietnam	Non-healthcare setting	COVID-19
Jia 2020(46)	Non-comparative	China	Healthcare setting	COVID-19
Jiang 2020(47)	Qualitative	China	Healthcare setting	COVID-19
Kang 2018(48)	Qualitative	South Korea	Healthcare setting	MERS
Kao 2004(49)	Qualitative	China	Healthcare setting	SARS
Khalid 2016(50)	Qualitative	Saudi Arabia	Healthcare setting	MERS
Khoo 2005(51)	Qualitative	China	Healthcare setting	SARS

Study ID[Reference]	Study Design	Country	Setting	Virus
Ki 2019(52)	Comparative NRS - Cohort	South Korea	Healthcare setting	MERS
Kim 2016(53)	Comparative NRS - Cohort	South Korea	Healthcare setting	MERS
Kinlay 2015(54)	Qualitative	United States of America	Healthcare setting	NA
Knapp 2008(55)	Qualitative	United States of America	Healthcare setting	Other
Lau 2003(56)	Qualitative	China	Non-healthcare setting	SARS
Lau 2004(57)	Comparative NRS - Cohort	China	Non-healthcare setting	SARS
Lau 2007(58)	Qualitative	China	Non-healthcare setting	Other
Li 2020(59)	Comparative NRS	China	Non-healthcare setting	COVID-19
Li 2020(60)	Non-comparative	China	Non-healthcare setting	COVID-19
Li 2020(61)	Non-comparative	China	Healthcare setting	COVID-19
Li 2020(62)	Comparative NRS	China	Non-healthcare setting	COVID-19
Li 2020(63)	Non-comparative	China	Non-healthcare setting	COVID-19
Li 2020(64)	Contextual factors - qualitative or quantiative	China	Non-healthcare setting	COVID-19
Lim 2004(65)	Qualitative	Singapore	Non-healthcare setting	SARS
Lin 2020(66)	Non-comparative	China	Non-healthcare setting	COVID-19
Liu 2009(67)	Comparative NRS - Cohort	China	Healthcare setting	SARS
Liu 2020(68)	Non-comparative	China	Non-healthcare setting	COVID-19
Liu 2020(69)	Non-comparative	China	Non-healthcare setting	COVID-19
Liu 2020(70)	Non-comparative	China	Non-healthcare setting	COVID-19
Liu 2020(71)	Comparative NRS	China	Non-healthcare setting	COVID-19
Liu 2020(72)	Comparative NRS	China	Non-healthcare setting	COVID-19
Liu 2020(73)	Comparative NRS	China	Non-healthcare setting	COVID-19
Loeb 2004(74)	Comparative NRS - Cohort	Canada	Healthcare setting	SARS
Loh 2004(75)	Qualitative	Malaysia	Healthcare setting	SARS
Lu 2003(76)	Non-comparative	China	Healthcare setting	SARS
Luo 2020(77)	Non-comparative	China	Non-healthcare setting	COVID-19
Ma 2004(78)	Comparative NRS	China	Healthcare setting	SARS

Study ID[Reference]	Study Design	Country	Setting	Virus
Ma 2020(79)	Comparative NRS	China	Healthcare setting	COVID-19
MacIntyre 2015(80)	RCT	Vietnam	Healthcare setting	Other
Maclntyre 2016(81)	RCT	China	Healthcare setting	Respiratory infectious diseases
Marchand-Senecal 2020(82)	Non-comparative - Case series	Canada	Healthcare setting	COVID-19
Maroldi 2017(83)	Qualitative	Brazil		Other
Matthews Pillemer 2015(84)	Qualitative	United States of America, China, Taiwan and Singapore	Non-healthcare setting	SARS
Moore 2005(85)	Qualitative	Canada	Healthcare setting	SARS
Mukerji 2017(86)	Qualitative	China	Healthcare setting	Respiratory infection (Clinical respiratory illness [CRI])
Nichol 2008(87)	Qualitative	Canada	Healthcare setting	SARS
Nichol 2013(88)	Qualitative	Canada	Healthcare setting	Occupational transmission
Nishiura 2005(89)	Comparative NRS - Cohort	Vietnam	Healthcare setting	SARS
Nishiyama 2008(90)	Comparative NRS	Vietnam	Healthcare setting	SARS
Ofner-Agostini 2006(91)	Non-comparative - Case series	Canada	Healthcare setting	SARS
Olsen 2003(92)	Comparative NRS - Cohort	China	Non-healthcare setting	SARS
Ong 2020(93)	Mechanistic	Singapore	Healthcare setting	SARS
Ou 2020(94)	Comparative NRS	China	Non-healthcare setting	COVID-19
Park 2004(95)	Comparative NRS - Cohort	United States of America	Healthcare setting	SARS
Park 2015(96)	Non-comparative - Case series	South Korea	Healthcare setting	MERS
Park 2016(97)	Comparative NRS - Cohort	South Korea	Healthcare setting	MERS
Park 2020(98)	Non-comparative	South Korea	Healthcare setting	MERS
Parker 2006(99)	Qualitative	Canada	Healthcare setting	SARS
Peck 2004(100)	Comparative NRS - Cohort	United States of America	Healthcare setting	SARS
Pei 2006(101)	Comparative NRS - Cohort	China	Healthcare setting	SARS
Qi 2020(102)	Contextual factors - qualitative or quantiative	China	Healthcare setting	COVID-19
Qian 2020(103)	Comparative NRS	China	Non-healthcare setting	COVID-19

Study ID[Reference]	Study Design	Country	Setting	Virus
Qian 2020(104)	Non-comparative	China	Healthcare setting	COVID-19
Qiu 2020(105)	Non-comparative	China	Non-healthcare setting	COVID-19
Rabaan 2017(106)	Qualitative	Saudi Arabia	Healthcare setting	MERS
Radonovich 2019(107)	Qualitative	United States of America	NR	Viral respiratory infections
Rea 2007(108)	Comparative NRS - Cohort	Canada	Non-healthcare setting	SARS
Reuss 2014(109)	Comparative NRS	Germany	Healthcare setting	MERS
Reynolds 2006(110)	Comparative NRS - Cohort	Vietnam	Healthcare setting	SARS
Rozenbojm 2015(111)	Qualitative	Canada	Healthcare setting	Other
Ryu 2019(112)	Comparative NRS - Cohort (but none infected)	South Korea	Healthcare setting	MERS
Scales 2003(113)	Comparative NRS	Canada	Healthcare setting	SARS
Seto 2003(114)	Comparative NRS - Cohort	China	Healthcare setting	SARS
Shen 2020(115)	Comparative NRS	China	Healthcare setting	COVID-19
Shigayeva 2007(116)	Qualitative	Canada	Healthcare setting	SARS
Siu 2016(117)	Qualitative	China	Healthcare setting	SARS
Sun 2020(118)	Non-comparative	China	Non-healthcare setting	COVID-19
Tan 2006(119)	Qualitative	Singapore	Healthcare setting	SARS
Tang 2004(120)	Qualitative	Hong Kong		SARS
Tang 2005(121)	Qualitative	Singapore	Healthcare setting	SARS
Teleman 2004(122)	Comparative NRS - Cohort	Singapore	Healthcare setting	SARS
Tian 2020(123)	Non-comparative	China	Healthcare setting	COVID-19
Timen 2010(124)	Qualitative	Netherlands	Healthcare setting	NA
Tuan 2007(125)	Comparative NRS - Cohort	Vietnam	Non-healthcare setting	SARS
Turnberg W 2008(126)	Qualitative	Washington	Healthcare setting	None
Twu 2003(127)	Non-comparative - Case series	Taiwan	Healthcare setting	SARS
Varia 2003(128)	Non-comparative - Case series	Canada	Healthcare setting	SARS
Visentin 2009(129)	Qualitative	Canada	Healthcare setting	SARS
Wang 2015(130)	RCT - Cluster RCT	Saudi Arabia	Non-healthcare setting	MERS and other respiratory viruses

Study ID[Reference]	Study Design	Country	Setting	Virus
Wang 2020(131)	Comparative NRS	China	Non-healthcare setting	COVID-19
Wang 2020(132)	Non-comparative	China	Non-healthcare setting	COVID-19
Wang 2020(133)	Comparative NRS	China	Non-healthcare setting	COVID-19
Wang 2020(134)	Contextual factors - qualitative or quantiative	China	Healthcare setting	COVID-19
Wiboonchutikul 2016(135)	Comparative NRS	Thailand	Healthcare setting	MERS
Wilder-Smith 2005(136)	Comparative NRS - Cohort	Singapore	Healthcare setting	SARS
Wizner 2016(137)	Qualitative	United States of America	Healthcare setting	SARS
Wong 2004(138)	Qualitative	China	NR	SARS
Wong 2005(139)	Qualitative	China	NR	SARS
Wong 2013(140)	Qualitative – RCT + EtD	China	NR	Other
Wu 2004(141)	Comparative NRS	China	Healthcare setting	SARS
Wu 2020(142)	Non-comparative	China	Non-healthcare setting	COVID-19
Wu 2020(143)	Qualitative	China	Healthcare setting	COVID-19
Wu 2020(144)	Non-comparative - Case series	China	Healthcare setting	COVID-19
Xiang 2020(145)	Non-comparative	China	Non-healthcare setting	COVID-19
Xiao 2020(146)	Non-comparative	China	Non-healthcare setting	COVID-19
Xie 2020(147)	Non-comparative - Case series	China	NR	COVID-19
Yang 2011(148)	Non-comparative + EtD	China	NR	Respiratory infection (Clinical respiratory illness [CRI])
Yang 2020(149)	Comparative NRS	China	Non-healthcare setting	COVID-19
Yang 2020(150)	Non-comparative	China	Healthcare setting	COVID-19
Yin 2004(151)	Comparative NRS - Cohort	China	Healthcare setting	SARS
Yu 2005(152)	Comparative NRS - Cohort	China	Healthcare setting	SARS
Yu 2007(153)	Comparative NRS - Cohort (cluster, not by patient)	China	Healthcare setting	SARS
Yu 2020(154)	Non-comparative	China	Non-healthcare setting	COVID-19
Yue 2020(155)	Non-comparative	China	Healthcare setting	COVID-19

Study ID[Reference]	Study Design	Country	Setting	Virus
Zeng 2020(156)	Comparative NRS	China	Non-healthcare setting	COVID-19
Zhang 2020(157)	Comparative NRS	China	Non-healthcare setting	COVID-19
Zhang 2020(158)	Non-comparative	China	Non-healthcare setting	COVID-19
Zhang 2020(159)	Non-comparative	China	Non-healthcare setting	COVID-19
Zhao 2020(160)	Comparative NRS	China	Healthcare setting	COVID-19
Zhou 2020(161)	Non-comparative	China	Healthcare setting	COVID-19
Zhou 2020(162)	Non-comparative	China	Non-healthcare setting	COVID-19
Zhu 2020(163)	Contextual factors - qualitative or quantiative	China	Non-healthcare setting	COVID-19
Zhuang 2020(164)	Non-comparative	China	Non-healthcare setting	COVID-19

Appendix 3. Newcastle-Ottawa for non-randomized studies, for the outcome of disease transmission

Study	Selection*	Comparability	Outcome/Exposure	Overall Rating (more stars = lower risk of bias)	Disease
Alraddadi 2016	★★★	★★	★★★	★★★★★★★★	MERS
Arwady 2016	★★★	-	★★★	★★★★★★	MERS
Bai 2020	★★	-	★★★	★★★★★	COVID-19
Burke 2020	★★★	-	★	★★★★	COVID-19
Caputo 2006	★★	-	★★★	★★★★★	SARS
Chen 2009	★★★★	★★	★	★★★★★★★	SARS
Cheng 2020	★★★	-	★★	★★★★★	COVID-19
Fan 2020	★★	-	★★	★★★★	COVID-19
Ha 2004	★★	-	-	★★	SARS
Hall 2014	★★★	-	-	★★★	MERS
Heinzerling 2020	★★	-	★★	★★★★	COVID-19
Ho 2004	★★★	★★	★★★	★★★★★★★★	SARS
Ki 2019	★★	★★	★★★	★★★★★★	MERS
Kim 2016	★★★★	-	★★	★★★★★★	MERS
Kim 2016	★★★★	-	★★	★★★★★★	MERS
Lau 2004	★★★	★★	★★	★★★★★★★	SARS
Liu 2009	★★★	★	★	★★★★★	SARS
Liu ZQ 2020	★★★★	-	★★★	★★★★★★★	COVID-19
Loeb 2004	★★	-	-	★★	SARS
Ma 2004	★★★★	★★	★★★	★★★★★★★★★	SARS
Nishiura 2005	★★★	★★	★★★	★★★★★★★★	SARS
Nishiyama 2008	★★	★★	★★	★★★★★★	SARS
Olsen 2003	★★★	-	★★★	★★★★★★	SARS
Park 2004	★★★★	★★	★★★★	★★★★★★★★★★	SARS

Park 2016	★★	-	★	★★★	MERS
Peck 2004	★★★★	★★	★★★	★★★★★★★★★	SARS
Pei 2006	★★★	★★	★★★	★★★★★★★★	SARS
Rea 2007	★★	-	★★	★★★★	SARS
Reuss 2014	★★★	-	★★	★★★★★	MERS
Reynolds 2006	★★	-	★	★★★	SARS
Ryu 2019	★★★	★	★★★	★★★★★★★	MERS
Scales 2003	★★	-	-	★★	SARS
Seto 2003	★★★★	★★	★★	★★★★★★★★	SARS
Teleman 2004	★★★★	★★	★★	★★★★★★★★	SARS
Tuan 2007	★★	★★	★★	★★★★★★	SARS
Wang QP 2020	★★★	-	★★	★★★★★	COVID-19
Wiboonchutikul 2016	★★	-	★★★	★★★★★	MERS
Wilder-Smith 2005	★★★	★★	★★★	★★★★★★★★	SARS
Wong TW 2004	★★★	-	★★	★★★★★	SARS
Wu 2004	★★★★	★★	★★	★★★★★★★★	SARS
Wu 2020	★★	-	★★	★★★★	COVID-19
Yin 2004	★★★★	★★	-	★★★★★★	SARS
Yu 2005	★★★	★	★★★	★★★★★★★	SARS
Yu 2007	★★★	★★	★★	★★★★★★★	SARS

*For each category, A single dash (-) indicates no stars, and therefore high risk of bias.

Appendix 4. Funnel plots

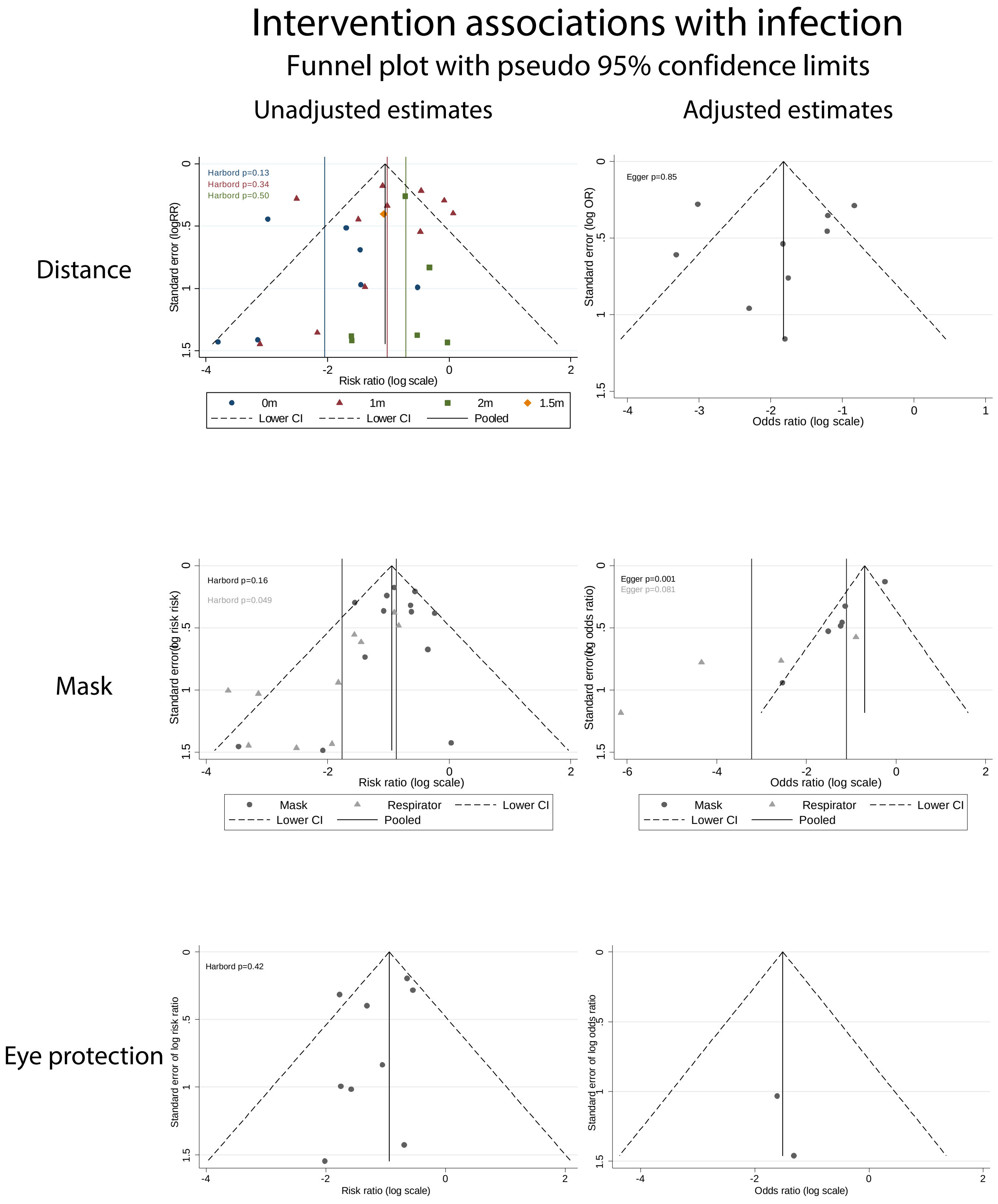

Appendix 5. Evidence Profiles

Author(s): Derek K. Chu, Elie Akl, Amena El-Harakeh, Antonio Bognanni, Tamara Lotfi, Mark Loeb, Aida Farha, Anisa Hajizadeh, Anna Bak, Ariel Izcovich, Carlos A. Cuello-Garcia, Chen Chen, David James Harris, Ewa Borowiack, Fatimah Chamseddine, Finn Schünemann, Gian Paolo Morgano, Giovanna Elsa Ute Muti Schünemann, Guang Chen, Hong Zhao, Ignacio Neumann, Jeffrey Chan, Joanne Khabsa, Layal Hneiny, Leila Harrison, Maureen Smith, Nesrine Rizk, Paolo Giorgi Rossi, Pierre AbiHanna, Rayane El-Khoury, Rosa Stalteri, Tejan Baldeh, Thomas Piggott, Yuan Zhang, Zahra Saad, Assem Khamis, Marge Reinap, Stephanie Duda, Karla Solo, Sally Yaacoub, Holger Schünemann
Question: Should physical distancing of more than one meter compared to one meter or less, masks versus no masks, and/or eye protection versus no eye protection be used to prevent disease transmission to people exposed to patients infected or suspected to be with COVID-19?
Setting: Any (Healthcare and non-healthcare)
Bibliography: Chu et al. prepared for publication

Certainty assessment							№ of patients		Effect		Certainty	Importance
№ of studies	Study design	Risk of bias	Inconsistency	Indirectness	Imprecision	Other considerations	Intervention	Control	Relative (95% CI)	Absolute (95% CI)		
Infection with COVID-19 (follow up: range 10 days to more days; assessed with: COVID-19, SARS, MERS infection)												
9	observational studies A physical distance of more than one meter vs less than one meter	not serious [a]	not serious [b]	not serious [c,d]	not serious	strong association [e,f]	97/5065 (1.9%) [g]	347/2717 (12.8%)	aOR 0.18 (0.09 to 0.38)	102 fewer per 1,000 (from 115 fewer to 75 fewer)	⨁⨁⨁◯ MODERATE	CRITICAL
10	observational studies Masks vs no masks	not serious i	not serious [h]	not serious [j]	not serious	none [k]	145/1066 (13.6%)	197/1134 (17.4%)	aOR 0.15 (0.07 to 0.34)	143 fewer per 1,000 (from 159 fewer to 107 fewer)	⨁⨁◯◯ LOW	CRITICAL
13	observational studies Eye protection (face shield, goggles)	not serious n	not serious [m]	not serious [o]	not serious	none [p]	62/1335 (4.6%)	388/2378 (16.3%)	RR 0.34 (0.22 to 0.52) [l]	108 fewer per 1,000 (from 127 fewer to 78 fewer)	⨁⨁◯◯ LOW	CRITICAL

CI: Confidence interval; OR: Odds ratio

a. All studies were non-randomized and evaluated using the Newcastle-Ottawa Scale. Some studies had higher risk of bias than others but there was no important difference in the sensitivity analyses excluding studies at higher risk of bias. We did not further rate down for risk of bias.
b. Although there was a high I2 value and lack of overlapping confidence intervals, all point estimates of the studies exceeded the thresholds for large effects and we did not rate down for inconsistency.
c. We did not rate down for indirectness for the association between distance and infection because the SARS and COVID-19 viruses all belong to the same family and have each caused epidemics with sufficient similarity; there was also no convincing statistical evidence of effect modification across viruses
d. Some studies included the use of masks, but subgroup analysis did not reveal important differences. Some studies also used bundled interventions and the effect of distances could not be evaluated in isolation but the studies shown here include only those that provide adjusted estimates. We did not rate down for intervention indirectness.
e. The effect is large considering the thresholds set by GRADE assuming that the odds ratios translate into similar magnitudes of relative risk estimates. This also mitigated concerns about risk of bias.
f. The data suggest a dose-response gradient with associations increasing from smaller distances to 2 meters and beyond. This was also suggested by a meta-regression. We did not rate up for this domain alone but in combination with the large effects.
g. One of the studies, did report the raw data but only the adjusted estimates.
h. Although there was a high I2 value, all point estimates of the studies were relatively large and the confidence intervals were overlapping and we did not rate down for inconsistency.
i. All studies were non-randomized and evaluated using the Newcastle-Ottawa Scale. Some studies had higher risk of bias than others but there was no important difference in the sensitivity analyses excluding studies at higher risk of bias. We did not further rate down for risk of bias.
j. We did not rate down for indirectness for the association between eye protection and infection because the SARS and COVID-19 belong to the same family and are considered sufficiently similar. Some studies also used bundled interventions and the effect of distances could not be evaluated in isolation but the studies shown here include only those that provide adjusted estimates. We did not rate down for intervention indirectness.
k. The effect is large considering the thresholds set by GRADE assuming that the odds ratio translate into similar magnitudes of relative risk estimates. This mitigate concerns about risk of bias but all studies were unadjusted and risk of bias still too high to rate up for large effects.
l. Two of these studies (Ma 2004 and Yin 2004) provided adjusted estimates with a total of 295 in the goggles group and 107 in the group not wearing goggles. The results were similar to the unadjusted estimate (OR 0.22, 95% CI 0.12 - 0.39).
m. Although there was a high I2 value, all point estimates of the studies were relatively large and the confidence intervals were overlapping and we did not rate down for inconsistency.
n. All studies were non-randomized and evaluated using the Newcastle-Ottawa Scale. Some studies had higher risk of bias than others but there was no important difference in the sensitivity analyses excluding studies at higher risk of bias. We did not further rate down for risk of bias.
o. We did not rate down for indirectness for the association between eye protection and infection because the SARS and COVID-19 belong to the same family and are considered sufficiently similar. Some studies also used bundled interventions and the effect of distances could not be evaluated in isolation but the studies shown here include only those that provide adjusted estimates. We did not rate down for intervention indirectness.
p. The effect is large considering the thresholds set by GRADE assuming that the odds ratio translate into similar magnitudes of relative risk estimates. This mitigate concerns about risk of bias but all studies were unadjusted and risk of bias still too high to rate up for large effects.

Appendix 6. Forest plots of additional analyses

Appendix 6. Forest plots of additional analyses

Association of exposure proximity with infection
Sub-divided by setting and intervention

Setting

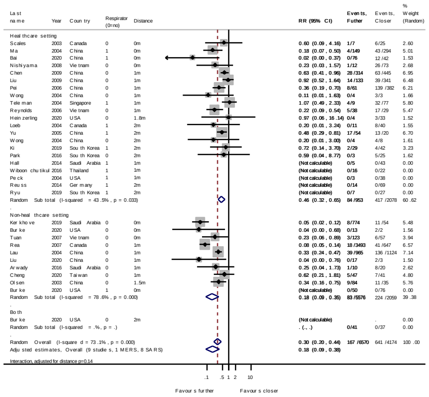

Association of mask use with infection
Sub-divided by population and setting

Virus

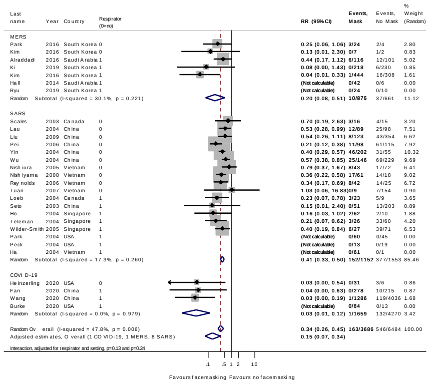

Association of eye protection with infection
Sub-divided by intervention

Unadjusted estimates by distance to infected individual

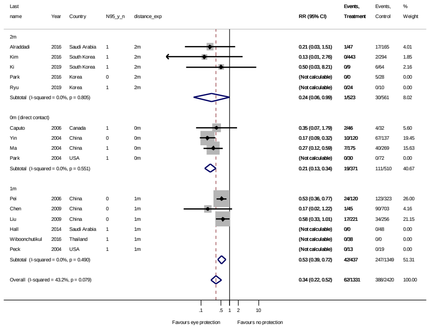

Facemask

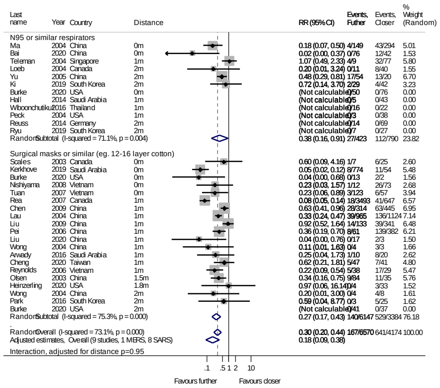

Facemask

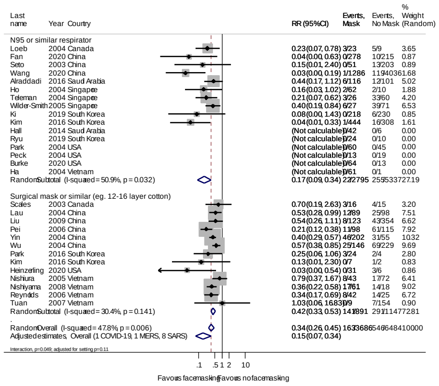

Unadjusted estimates by N95 or similar (1) or not (0)

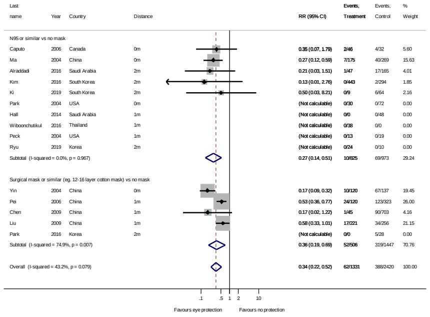

Appendix 7. Sensitivity analyses, and Bayesian Meta-analyses

	Distancing		Masks		Eye protection	
	Unadjusted	Adjusted	Unadjusted	Adjusted	Unadjusted	Adjusted
**Sensitivity analyses**						
**Bayesian** Influenza RCTs (mean=0.93, SD of logRR=0.57)			0.54 (95%CrI 0.43-0.82)	0.40 (95%CrI 0.16-0.97)		
**Exclude Preprints**	0.32 (0.21-0.48)	0.15 (0.07-0.31)	0.38 (0.31-0.48)	0.21 (0.10-0.43)	0.34 (0.22-0.52)	0.22 (0.12-0.39)
**Fixed effect model**	0.34 (0.29-0.40)	0.16 (0.12-0.22)	0.32 (0.27-0.38)	0.16 (0.12-0.22)	0.36 (0.28-0.46)	0.22 (0.12-0.39)
**Hartung-Knapp-Sidik-Jonkman random effects model**	0.30 (0.20-0.44)	0.15 (0.08-0.30)	0.34 (0.25-0.47)	0.15 (0.08-0.30)	0.34 (0.22-0.51)	0.22 (0.04-1.27)

Bayesian meta-analysis if MacIntyre 2013(165) cluster RCT used as likelihood function (OR 0.50 [95%CI 0.34-0.74]), posterior probability for OR<1 of N95 masks being more protective versus medical masks = 98.4%.

Pooled unadjusted odds ratios were similar to risk ratios:
Distancing: OR 0.22 (0.14- 0.35)
Masks: OR 0.22 (0.15- 0.32)
Eye protection: OR 0.26 (0.16-0.45)

Exclusion of Seto from adjusted estimates, because about 54% of its population used N95 masks, did not change the findings: aOR 0.03 (0.001-0.56)

The pooled aORs for studies with the various types of facemasks were:
N95 or similar respirators: 0.04 (0.004-0.30)

versus

Surgical masks: 0.20 (0.06-0.63)
12-16 multilayer cotton masks: 0.33 (0.10-1.03)
Surgical masks or multilayer cotton masks: 0.31 (0.16-0.53)
Test for interaction of surgical versus multilayer cotton masks, $p_{interaction} = 0.91$

Appendix 8. Credibility assessment of potential effect modifiers (modified from GRADE inconsistency guidelines to include 'other considerations')

**Outcome**	COVID-19, SARS, MERS viral transmission		
**Potential effect modifier**<br><br>**Criteria**	Distance dose-response	N95 or similar versus surgical mask or similar (eg. 12-16 layer cotton)	Healthcare versus non-healthcare settings for mask use
Is the subgroup variable a characteristic specified at baseline (in contrast with after randomization)?	Yes	Yes	Yes
Is the subgroup difference suggested by comparisons within rather than between studies?	No	Yes, the included studies report a potential hierarchy of least protective being no mask, paper mask, disposable or 12-16 layer reusable cotton mask, then N95 or similar respirator	No
Does statistical analysis suggest that chance is an unlikely explanation for the subgroup difference?	Possibly, mean > 1 with wide CIs expected from few studies at each cut point, p=0.041	Yes, p=0.033<br><br>Bayesian analyses also support this with posterior probability of RR<1 being >95%.	Possibly, p=0.049 in univariate meta-regression, and when controlling for differential N95 use between settings, still low at p=0.11
Did the hypothesis precede rather than follow the analysis, and include a hypothesized direction that was subsequently confirmed?	Yes	Yes	Yes
Was the subgroup hypothesis one of a small number tested?	Yes	Yes	Yes
Is the subgroup difference consistent across studies and across important outcomes?	Consistent with findings with other interventions presented here	Yes across studies	No
Does external evidence (biological or sociological rationale) support the hypothesized subgroup difference?	Yes, it would be expected that the further away one is from a person with infection that transmits by droplets, that the further distances lead to decreased risk of infection.	The increased filtration capacity of respirators would be expected to have enhanced protection against viral droplets, or smaller versions of such droplets or aerosols.	Possibly, some hypothesize that mask use in non-healthcare settings can lead to self-inoculation virus through mechanisms such as improper use or touching the mask with contaminated hands, but there is no definitive evidence with hard outcomes that community-based mask interventions are ineffective or harmful.
Absence of other considerations that would decrease confidence of true effect-modification?	Imprecision. Association primarily based on unadjusted data, albeit estimates of unadjusted and adjusted data were similar.	Although influenza is very different from pandemic COVID-19, SARS, MERS, it provides very indirect and limited RCT data suggesting no difference, albeit the Bayesian analyses here attempt to account for that.	Imprecision, particularly few community-focused studies
**Criteria fulfilled, out of 8 (not an absolute cutoff)**	**5**	**6-7**	**3-4**
**Overall credibility of subgroup analysis**	**MODERATE**	**MODERATE-HIGH**	**LOW-MODERATE**

Low credibility, likely spurious; Moderate credibility, plausible, possibly even likely, but some important doubt remains; High credibility, Likely convincing.

Appendix 9. Summary of contextual factor data

Resource use

Two qualitative and two cross-sectional studies reported on data related to the cost and resource use in the management of SARS (51, 65), MERS (5) and coronavirus (83). The four studies were conducted in Hong Kong, Brazil, Singapore and Saudi Arabia. Khoo et al. (2005) reported the cost of 3M powered air-purifying respirators (PAPR) to be US$860 and Stryker PARP US$580 as compared to N95 (US$0.70) (51). In another study, health workers perceived the management of SARS as a burden which costs hundreds of millions; with direct operating expenditure (e.g., medical supplies, personal protective equipment, and screening) costing US$110 million(65). Malordi et al. (2017) highlighted the consequences of the lack of resources which include inadequate training on measures to prevent disease transmission(83). Al-Tawfiq et al. (2019) highlighted a monthly added cost of $16,400 for infection control items, such as hand sanitizers, soap, surgical masks, and N95 respirators during MERS outbreak in one hospital in Saudi Arabia (5). A survey of health workers in a hospital (doctors, nurses and respiratory therapists, n=51) showed that the majority of health workers (84%) preferred using PAPR over N-95 respirators when treating suspected SARS patients despite its high cost (51).

Acceptability

Six qualitative studies conducted in China and one cross-sectional study conducted in Vietnam reported on the acceptability of physical distancing and/or wearing masks as preventive measures for COVID-19.

Acceptability by visitors of suspected or confirmed COVID-19 cases

Wang et al. (2020) carried out an online survey to investigate the protective behaviors of visitors accompanying hospitalized patients during COVID-19 pandemic (134). 208 questionnaires were collected, and the survey showed that 85% of visitors accompanying suspected COVID-19 cases wear masks while present in the hospital.

Acceptability by the public

Four qualitative studies presented information on the willingness of residents in China to wear masks in public places and to avoid crowds (18, 64, 102, 166). The four studies used online questionnaires to survey members of the public and the samples were respectively, n=1,138 (64), n=917 (166), n=3,083 (102), and n=4,016 (18). Across the four studies, most of the participants reflected high willingness to wear masks in public places (95%, 99%, 97%, 94% respectively). In terms of social gatherings, the majority of the participants across three of the studies favored avoiding crowded areas (91%, 96%, 97% respectively) (18, 64, 102).

Another survey conducted in Vietnam (n=345) found that the risk perception of COVID-19 threat significantly increased the likelihood of wearing medical masks ($p<0.01$). The increased likelihood of wearing masks was also shown to increase with age (45).

Acceptability by college students

A survey to assess the knowledge and protective behaviors among college students (n=22,302 online questionnaires) in China during COVID-19 pandemic(12), found that 99% of students were willing to avoid close contact with others (less than 1 meter), 95% considered avoiding crowded places as an important way to control the epidemic, and 99% reported wearing a mask in public places for week prior to being surveyed.

Acceptability by healthcare workers

A cross-sectional survey (56) performed in the context of the SARS epidemic in Hong Kong, assessed various precautionary measures from the viewpoint of 1,397 residents. Most of the respondents believed that SARS could be transmitted via direct body contact with patients (84%) and via respiratory droplets (97%). The perceived risk of transmission increased during the escalating phase of the epidemic (52%) and declined during a later stage (36%).

During the first phase of the epidemic, respondents reported a significant increase in the application of preventive measures such as avoiding going outside and avoiding crows, which dropped at a later stage. Those who perceived avoiding crowded places as an effective preventive measure (OR: 31.564, 95% CI: 15.610 -63.824) were likely to avoid crowded places. In terms of the acceptability of wearing masks, most of the respondents (95%) regarded this action as a ‘civic responsibility’ and reflected commitment to wearing masks in public places. Those who perceived wearing a mask as an efficacious means of prevention (OR: 7.151, 95% CI: 4.245-12.045) were more likely than others to wear a mask (56).

Five studies conducted on health professionals (including medical staff and nurses) in primary health care and hospital settings showed that an increase in the perceptions and awareness of risk of transmission of SARS was associated with better adherence to preventive measures including wearing masks and eye protection (32, 75, 83, 88, 116).

A cross-sectional quantitative survey of dental health professionals (n=406) working in dental facilities in Saudi Arabia showed good practices related to making patients with MERS infection wear masks during transport (84%). However, knowledge was relatively limited (56.4%) about the need to wear a mask within a 90 cm distance from a patient under droplet precaution care (10). Another cross-sectional survey of health workers (N=10,236) was conducted about the appropriateness of using PAPR and N95 respirators in

public hospitals and polyclinics during the SARS outbreak in Singapore (23). Among doctors (n=873), nurses (n=4,404), and clerical staff (n=921), 99.5%, 99% and 97% respectively viewed N95 respirator to be an adequate protection against SARS.

A cross-sectional study (two surveys) was conducted to assess the use of personal protective equipment among medical students during and after the SARS outbreak in a teaching hospital in Hong Kong and study its impact on their personal hygiene practice when they contacted patients (139). Prior to the SARS outbreak, none of the students wore masks during history taking and physical examination. In the 2004 survey, 86.1% and 93.8% of students wore masks during history taking and physical examination, respectively.

Another study (secondary data analysis) conducted in Saudi Arabia evaluating the use of masks before and during MERS showed an increase in the use of both, surgical masks (from 2,947.4 to 10,283.9 per 1,000 patient-days) and N-95 respirators (from 22 to 232 per 1,000 patient-days) ($p < .0000001$) (5).

Feasibility

In this section, we summarized barriers and facilitators to the implementation and sustainability of using masks based on findings from the included studies. Among barriers, we identified:

Barriers to the use of protective masks

A study showed that N-95 respirators was perceived by health workers as uncomfortable during the SARS outbreak (48). N95 respirators often developed cracks in the chin area for small-jawed female health professionals and the overlapping parts of different PPE items were ill-fitted (e.g., gaps between goggles and N95 respirator) (48).

Family physicians (n=7) in Singapore stressed on the physical discomfort during prolonged use of the N-95 mask (e.g., breathing difficulty, headache, development of allergic facial rash around the mask) in a qualitative study employing interviews about factors that influence the use of PPE during the SARS outbreak (119) . In this study, family physicians in Singapore also showed that the use of the N-95 mask led to difficulty in communication with patients who had adverse reaction (i.e., worries and concern as PPE was a sign that the physician could have been exposed to SARS) (119). In addition, Khoo et al. (2005) showed that PAPR made most of the health workers (64%) feel that they looked frightening to their patients when using it (51).

Another qualitative study used 15 focus group discussions to examine the perceptions of health workers (n=105) in Canada regarding factors associated with self-protective behavior during the SARS outbreak (85). This study identified mask fitting and uncomfortable PPE to be among the barriers to effective use of PPE.

Absence of a monitoring system

Moore et al. (2005) showed that barriers to the use of protective wear included deficiencies in the tracking system to monitor the development, delivery and evaluation of training in infection control (85).

Lack of adherence to available guidance

In a qualitative study among health professionals (n=26) in the Netherlands about barriers to implementing infection prevention and control guidelines during crises, respondents highlighted the below as potential reasons for the lack of adherence to guidelines during outbreaks such as SARS (124):

- lack of imperative or precise wording
- lack of easily identifiable instructions specific to each profession
- lack of concrete performance targets
- lack of timely and adequate guidance on personal protective equipment and other safety measures

Other barriers that were described in the included studies were the shortage of PPE and cost due to bulk purchase (119), lack of consistent policies for quarantining individuals, reuse of masks, and deficiencies in decision regarding the assignment of patients to negative pressure rooms (85).

Facilitators to the use of protective masks

Most of the health workers perceived both types of PAPR (3M and Stryker) to be easy or relatively easy to use (74% and 91%) with an acceptable level of visual impairment attributable to the PAPR (98% and 95% for the 3M and Stryker PAPR, respectively) (51).

Perceived susceptibility and perceived benefits

A survey about factors influencing the wearing of facemasks for the prevention of SARS among adult Chinese (n=1329) in Hong Kong showed that 61% of respondents reported consistent use of facemasks to prevent SARS and the following predicting factors (120):

- Awareness of the risks and serious consequences associated with SARS: respondents who felt more susceptible to contracting SARS (OR = 2.575; CI = 1.586, 4.181) and those who perceived SARS as having more serious consequences (OR =1.176; CI = 0.909, 1.521) were more likely to wear facemasks.
- Awareness of the benefits of wearing facemasks: respondents who believed greater benefits in wearing facemasks (OR = 1.354; CI = 1.019, 1.800) were more likely to wear facemasks.

Appendix 10. PROSPERO Registration number

Registration number CRD42020177047

A rapid systematic review of physical distancing with or without masks and with or without eye protection to prevent COVID-19 transmission between patients with confirmed COVID-19 infection and other people, including health care workers

Holger Schunemann, Derek Chu, Elie Akl, Mark Loeb, Sally Yaacoub, Layal Hneiny, Neera Bhatnagar, Aida Farha, Ray Yuan Zhang, Ariel Izcovich, Ignacio Neumann, Carlos Cuello Garcia, Finn Schünemann, Giovanna Muti-Schünemann, Gian Paolo Morgano, Tamara Lotfi, Thomas Piggott, Ewa Borowiack, Anna Bak, Tejan Baldeh, Rosa Stalteri, Anisa Hajizadeh, Leila Harrison, Hong Zhao, Guang Chen, Antonio Bognanni, Marge Reinap, Paolo Giorgi Rossi

Citation

Holger Schunemann, Derek Chu, Elie Akl, Mark Loeb, Sally Yaacoub, Layal Hneiny, Neera Bhatnagar, Aida Farha, Ray Yuan Zhang, Ariel Izcovich, Ignacio Neumann, Carlos Cuello Garcia, Finn Schünemann, Giovanna Muti-Schünemann, Gian Paolo Morgano, Tamara Lotfi, Thomas Piggott, Ewa Borowiack, Anna Bak, Tejan Baldeh, Rosa Stalteri, Anisa Hajizadeh, Leila Harrison, Hong Zhao, Guang Chen, Antonio Bognanni, Marge Reinap, Paolo Giorgi Rossi. A rapid systematic review of physical distancing with or without masks and with or without eye protection to prevent COVID-19 transmission between patients with confirmed COVID-19 infection and other people, including health care workers. PROSPERO 2020 CRD42020177047 Available from:
https://www.crd.york.ac.uk/prospero/display_record.php?ID=CRD42020177047

Review question

From patients infected with COVID-19, what distance can the COVID-19 virus travel (mechanistic question)? What is the impact on people maintaining at least one meter distance compared to a smaller distance from a patient or suspected patient with COVID-19 on droplet transmission (intervention question)?

Sub-questions:

(1) With or without a mask on the patient;

(2) With or without a mask and with or without eye protection on the non-infected person

Searches

We will search the following electronic databases:

• PubMed, MEDLINE, EMBASE, CINAHL, and the Cochrane Library from 2019 to current date.

We will search the following Chinese electronic databases:

• WHO Chinese database

• CNKI (http://new.oversea.cnki.net/index/)

• China Biomedical Literature Service (http://www.sinomed.ac.cn/login.do)

In addition, we will search the following COVID-19 specific databases from 2019 to current date

• Epistemonikos COVID-19 L·OVE platform
(https://app.iloveevidence.com/loves/5e6fdb9669c00e4ac072701d);

• EPPI Centre living systematic map of the evidence (http://eppi.ioe.ac.uk/cms/Projects/DepartmentofHealthandSocialCare/Publishedreviews/COVID-19Livingsystematicmapoftheevidence/tabid/3765/Default.aspx);

• CORD-19 (https://www.kaggle.com/allen-institute-for-ai/CORD-19-research-challenge);

• COVID-19 Research Database maintained by the World Health Organization (https://www.who.int/emergencies/diseases/novel-coronavirus-2019/global-research-on-novel-coronavirus-2019-ncov)

We will conduct a search for ongoing trials using the U.S. National Library of Medicine Register of Clinical Trials (ClinicalTrials.gov) and the WHO International Clinical Trials Registry Platform (ICTRP). We will hand-search the reference lists of the included papers. We will also review the studies included in any identified relevant systematic reviews.

Search strategy combines relevant medical subject headings (MeSH) and keywords, which include “COVID-19”, and “corona virus”. PubMed search terms are informed by https://blocks.bmi-online.nl/catalog/397. The search strategy has been drafted by Ms. Layal Hneiny and is being peer reviewed by two information specialists (Ms. Neera Bhatnagar and Ms. Aida Farha). Finalized search strategies will be available on March 26, 2020 but the final draft can be found in the appendix.

Content experts will search websites of governmental and organizational websites for relevant grey literature documents.

Additional search strategies to identify indirect evidence on SARS and MERS will also be constructed and peer-reviewed by information specialists. This latter search will focus on systematic reviews.

Types of study to be included

No restrictions will be placed on study design. However, evidence will be prioritized by study design as follows: i) randomized controlled trials; ii) non-randomized comparative studies; iii) non-comparative studies (i.e., case reports, case series); iv) qualitative studies. We will exclude single case reports if non-randomized studies comparative studies provide the same certainty of evidence. We will also review modelling studies. For the question addressing how far the virus can travel we will consider mechanistic human studies.

Condition or domain being studied

Infections and infestations, respiratory disorders

Participants/population

Studies focused on patients with confirmed COVID-19 infection [or SARS or MERS] and people in close contact with them, including health care workers, will be eligible for inclusion. Other related populations to consider are:

• individuals with suspected COVID-19 infection who are waiting to be tested (e.g., presenting to a lab, emergency department, or dedicated clinic to get tested), or cannot be tested (because of lack of resources)

• individuals with suspected or confirmed COVID-19 infection (whether symptomatic or not) who are in isolation in non-healthcare settings (e.g., at home, and other dedicated spaces such as stadiums and tents)?

Intervention(s), exposure(s)

At least one meter distance between people and COVID-19 infected patients:

(1) With or without a mask on the patient;

(2) With or without a mask and with or without eye protection on the HCW.

Subgroups:

• Masks include surgical mask and N95 mask among others; Similar names for N95 are:

o FFP2 (Europe EN 149-2001)

o KN95 (China GB2626-2006)

o P2 (Australia/New Zealand AS/NZA 1716:2012)

o Korea 1st class (Korea KMOEL - 2017-64)

o DS (Japan JMHLW-Notification 214, 2018)

• Eye protection include visors, shields, and goggles among others

Comparator(s)/control

less than one meter of physical distancing

Main outcome(s)

· Transmission
· Risk of transmission to members of the community (herd immunity)
· Acceptability by different stakeholders (patient, HCW, individuals handling the dead bodies, health authorities) (e.g., possibly as a surrogate for harms if people are not wearing masks or eye protection)
· Unintended harms of distancing (e.g., when providing care) and of using masks or eye protection, stigmatization
· COVID19 infection (confirmed)
· COVID19 probable case
· ICU admission
· Hospitalization
· Death
· (Time to) Recovery

* Measures of effect

relative risks, odds ratios, risk difference, narrative summary

Additional outcome(s)

Droplet transmission (as measured by infection of others and confirmed by serological or microbiological or virolgical testing)

* Measures of effect

narrative

Data extraction (selection and coding)

A single reviewer will extract data using a piloted form and a second reviewer will verify all extracted data. Minimal data will be extracted addressing the following domains: study identifier; study design; setting; population characteristics; intervention and comparator characteristics; outcomes (quantitative if possible); source of funding and reported conflicts of interests; ethical approval; study limitations or other important comments.

Risk of bias (quality) assessment

One reviewer will perform risk of bias assessments and a second reviewer will verify all assessments. We will use the Cochrane risk of bias tool (version 2) for randomized controlled trials, and Newcastle Ottawa scale for non-randomized studies.

Strategy for data synthesis

We will synthesize data in both tabular and narrative formats. We anticipate our outcomes to be dichotomous, such as transmission, and therefore they will be analyzed as pooled risk ratios (RRs), if they are unadjusted estimates. If there are adjusted odds ratios from multivariable regression reported in the studies, then these will be pooled as adjusted odds ratios (aORs). These will be summarized using random effects meta-analysis using the DerSimonian and Laird random effects model, with heterogeneity calculated from the Mantel-Haenszel model. If there are time to event outcomes, shared frailty cox prorportional hazards models will be completed, with validation of the assumption of proportionality. This may necessitate digitization of Kaplan-Meier curves from published studies. All summary measures will be reported with an accompanying 95% confidence interval.

We anticipate that traditional statistical measures of heterogeneity will be less informative than established criteria per GRADE. Because of the poor performance of I^2 to quantify true heterogeneity, then we will accept

any magnitude of I² for meta-analysis. Nevertheless, we will collect the I² statistic, but comment on its limitations in the presentation of final product. We will also accept any number of study for comparative or non comparative meta-analysis. Summary measures will include absolute and relative risks for the outcomes outlined above, displayed using funnel plots and calculated using random effects models. Publication bias will also be assessed visually using funnel plots and Harbord's modification to Egger test, or if adjusted odds ratios are used, then Egger's original test. If necessary, mean and SD will be calculated from medians and IQR or range by the method of Wan (BMC Medical Research Methodology201414:135).

If there are only non-comparative studies, then we will meta-analyze these by proportions (ie. incidence of outcome per report [eg. numerator=events of transmission, denominator=total exposed]. In the presence of sparse data, we will give preference to the logit transformation when completing this, otherwise we will use the Freeman-Tukey double arcsine transformation.

The synthesis of contextual factors (acceptability, etc.) will be narrative.

Subgroup effects will be analysed by meta-regression with tests of interaction by 10, 000 Monte-Carlo permutations to calculate p values to avoid spurious findings.

Sensitivity analyses will include analysis by fixed effect and Knapp-Hartung-Sidik-Jonkman random effects model. We will also employ Bayesian meta-analyses of existing literature on the efficacy of mask use to prevent viral transmission, using as charitable assumptions as plausible that the RCT data represent the true effect estimates. This will include shrinking the effect estimate of the observational data, decreasing its weight (ie. increasing its variance as a prior) or both. We will also employ noninformative priors.

Data analyses will be performed using STATA 14.3. GRADEpro GDT will be used to construct the summary of findings table.

The analyses and reporting of the review will be done according to the PRISMA and MOOSE guidelines. A single reviewer will grade the certainty of the evidence using the GRADE approach and a second reviewer will verify all assessments. If applicable, we will follow published guidance for rating the certainty in evidence in the absence of a single estimate of effect. Evidence will be presented using GRADE Evidence Profiles developed in the GRADEpro (www.gradepro.org) software.

Analysis of subgroups or subsets

Health care workers versus non health care workers, by mask type, with or without goggles or eye protection

Contact details for further information

Holger Schunemann
schuneh@mcmaster.ca

Organisational affiliation of the review

McMaster University

Review team members and their organisational affiliations

Professor Holger Schunemann. McMaster University
Derek Chu. McMaster University
Elie Akl. American University of Beirut
Mark Loeb. McMaster University
Sally Yaacoub. American University of Beirut
Layal Hneiny. American University of Beirut
Neera Bhatnagar. McMaster University
Aida Farha. American University of Beirut
Ray Yuan Zhang. McMaster University
Ariel Izcovich. German Hospital, Buenos Aires
Ignacio Neumann. Pontificia Universidad Católica de Chile
Carlos Cuello Garcia. McMaster University
Finn Schünemann. None

Giovanna Muti-Schünemann. Vita-Salute San Raffaele University
Gian Paolo Morgano. McMaster University
Tamara Lotfi. McMaster University
Thomas Piggott. McMaster University
Ewa Borowiack. EvidencePrime, Inc
Anna Bak. EvidencePrime, Inc
Tejan Baldeh. McMaster University
Rosa Stalteri. McMaster University
Anisa Hajizadeh. McMaster University
Leila Harrison. McMaster University
Hong Zhao. the Institute of Acupuncture and Moxibustion, China Academy of Chinese Medical Sciences
Guang Chen. Dongzhimen Hospital, Beijing University of Chinese Medicine
Antonio Bognanni. None
Marge Reinap. WHO Regional Office for Europe
Paolo Giorgi Rossi. Azienda USL – IRCCS di Reggio Emilia

Collaborators

Stephanie Duda. McMaster University
Karla Solo. McMaster University

Type and method of review

Epidemiologic, Meta-analysis, Narrative synthesis, Systematic review

Anticipated or actual start date

25 March 2020

Anticipated completion date

28 April 2020

Funding sources/sponsors

World Health Organization, McMaster University, and American University of Beirut

Conflicts of interest

Language

English

Country

Argentina, Canada, Chile, China, Denmark, Germany, Italy, Lebanon

Stage of review

Review Ongoing

Subject index terms status

Subject indexing assigned by CRD

Subject index terms

COVID-19; Health Personnel; Humans; Infections; Masks; severe acute respiratory syndrome coronavirus 2

Date of registration in PROSPERO

16 April 2020

Date of first submission

28 March 2020

Stage of review at time of this submission

Stage	Started	Completed
Preliminary searches	Yes	No
Piloting of the study selection process	Yes	No
Formal screening of search results against eligibility criteria	Yes	No
Data extraction	No	No
Risk of bias (quality) assessment	No	No
Data analysis	No	No

The record owner confirms that the information they have supplied for this submission is accurate and complete and they understand that deliberate provision of inaccurate information or omission of data may be construed as scientific misconduct.

The record owner confirms that they will update the status of the review when it is completed and will add publication details in due course.

Versions

16 April 2020

PROSPERO
This information has been provided by the named contact for this review. CRD has accepted this information in good faith and registered the review in PROSPERO. The registrant confirms that the information supplied for this submission is accurate and complete. CRD bears no responsibility or liability for the content of this registration record, any associated files or external websites.

Appendix 11. PRISMA checklist

Section/topic	#	Checklist item	Reported on page #
**TITLE**			
Title	1	Identify the report as a systematic review, meta-analysis, or both.	1
**ABSTRACT**			
Structured summary	2	Provide a structured summary including, as applicable: background; objectives; data sources; study eligibility criteria, participants, and interventions; study appraisal and synthesis methods; results; limitations; conclusions and implications of key findings; systematic review registration number.	2
**INTRODUCTION**			
Rationale	3	Describe the rationale for the review in the context of what is already known.	4
Objectives	4	Provide an explicit statement of questions being addressed with reference to participants, interventions, comparisons, outcomes, and study design (PICOS).	4-6
**METHODS**			
Protocol and registration	5	Indicate if a review protocol exists, if and where it can be accessed (e.g., Web address), and, if available, provide registration information including registration number.	4
Eligibility criteria	6	Specify study characteristics (e.g., PICOS, length of follow-up) and report characteristics (e.g., years considered, language, publication status) used as criteria for eligibility, giving rationale.	4-6
Information sources	7	Describe all information sources (e.g., databases with dates of coverage, contact with study authors to identify additional studies) in the search and date last searched.	5-6
Search	8	Present full electronic search strategy for at least one database, including any limits used, such that it could be repeated.	Appendix
Study selection	9	State the process for selecting studies (i.e., screening, eligibility, included in systematic review, and, if applicable, included in the meta-analysis).	5-6
Data collection process	10	Describe method of data extraction from reports (e.g., piloted forms, independently, in duplicate) and any processes for obtaining and confirming data from investigators.	5-7
Data items	11	List and define all variables for which data were sought (e.g., PICOS, funding sources) and any assumptions and simplifications made.	5-7
Risk of bias in individual studies	12	Describe methods used for assessing risk of bias of individual studies (including specification of whether this was done at the study or outcome level), and how this information is to be used in any data synthesis.	7
Summary measures	13	State the principal summary measures (e.g., risk ratio, difference in means).	7-8
Synthesis of results	14	Describe the methods of handling data and combining results of studies, if done, including measures of consistency (e.g., I^2) for each meta-analysis.	7-8

Section/topic	#	Checklist item	Reported on page #
Risk of bias across studies	15	Specify any assessment of risk of bias that may affect the cumulative evidence (e.g., publication bias, selective reporting within studies).	7-8
Additional analyses	16	Describe methods of additional analyses (e.g., sensitivity or subgroup analyses, meta-regression), if done, indicating which were pre-specified.	7-8
**RESULTS**			
Study selection	17	Give numbers of studies screened, assessed for eligibility, and included in the review, with reasons for exclusions at each stage, ideally with a flow diagram.	9, Fig 1
Study characteristics	18	For each study, present characteristics for which data were extracted (e.g., study size, PICOS, follow-up period) and provide the citations.	9, Table 1, Appendix
Risk of bias within studies	19	Present data on risk of bias of each study and, if available, any outcome level assessment (see item 12).	10, Table 1, Appendix
Results of individual studies	20	For all outcomes considered (benefits or harms), present, for each study: (a) simple summary data for each intervention group (b) effect estimates and confidence intervals, ideally with a forest plot.	10-12, Fig 2-4
Synthesis of results	21	Present results of each meta-analysis done, including confidence intervals and measures of consistency.	10-12, Figs 2-4 Table 2
Risk of bias across studies	22	Present results of any assessment of risk of bias across studies (see Item 15).	Table 2, Appendix
Additional analysis	23	Give results of additional analyses, if done (e.g., sensitivity or subgroup analyses, meta-regression [see Item 16]).	10-12, Appendix
**DISCUSSION**			
Summary of evidence	24	Summarize the main findings including the strength of evidence for each main outcome; consider their relevance to key groups (e.g., healthcare providers, users, and policy makers).	13
Limitations	25	Discuss limitations at study and outcome level (e.g., risk of bias), and at review-level (e.g., incomplete retrieval of identified research, reporting bias).	16
Conclusions	26	Provide a general interpretation of the results in the context of other evidence, and implications for future research.	17
**FUNDING**			

Funding	27	Describe sources of funding for the systematic review and other support (e.g., supply of data); role of funders for the systematic review.	8

From: Moher D, Liberati A, Tetzlaff J, Altman DG, The PRISMA Group (2009). Preferred Reporting Items for Systematic Reviews and Meta-Analyses: The PRISMA Statement. PLoS Med 6(7): e1000097. doi:10.1371/journal.pmed1000097

For more information, visit: **www.prisma-statement.org**.

Page 2 of 2

Appendix 11 continued – MOOSE checklist

**Reporting of background should include**	Page/Location
Problem definition	3-4
Hypothesis statement	3-4
Description of study outcome(s)	6
Type of exposure or intervention used	5-6
Type of study designs used	5-6
Study population	5-6
**Reporting of search strategy should include**	
Qualifications of searchers (eg, librarians and investigators)	5-6, Appendix
Search strategy, including time period included in the synthesis and keywords	5-6, Appendix
Effort to include all available studies, including contact with authors	5-6, Appendix
Databases and registries searched	5-6, Appendix
Search software used, name and version, including special features used (eg, explosion)	5-6, Appendix
Use of hand searching (eg, reference lists of obtained articles)	5-6, Appendix
List of citations located and those excluded, including justification	Figure 1, Appendix
Method of addressing articles published in languages other than English	5-6
Method of handling abstracts and unpublished studies	5-6
Description of any contact with authors	5-6
**Reporting of methods should include**	
Description of relevance or appropriateness of studies assembled for assessing the hypothesis to be tested	5-6
Rationale for the selection and coding of data (eg, sound clinical principles or convenience)	5-7
Documentation of how data were classified and coded (eg, multiple raters, blinding, and interrater reliability)	5-7
Assessment of confounding (eg, comparability of cases and controls in studies where appropriate)	5-7
Assessment of study quality, including blinding of quality assessors; stratification or regression on possible predictors of study results	7
Assessment of heterogeneity	7
Description of statistical methods (eg, complete description of fixed or random effects models, justification of whether the chosen models account for predictors of study results, dose-response models, or cumulative meta-analysis) in sufficient detail to be replicated	7-8
Provision of appropriate tables and graphics	Figures 1-4, Table 1-2, Appendix
**Reporting of results should include**	
Graphic summarizing individual study estimates and overall estimate	Figures 2-4, Appendix
Table giving descriptive information for each study included	Table 1, Appendix
Results of sensitivity testing (eg, subgroup analysis)	9-12, Appendix
Indication of statistical uncertainty of findings	9-12, Figures 2-4, Table 2
**Reporting of discussion should include**	
Quantitative assessment of bias (eg, publication bias)	16
Justification for exclusion (eg, exclusion of non-English-language citations)	16
Assessment of quality of included studies	Table 2, 13
**Reporting of conclusions should include**	
Consideration of alternative explanations for observed results	16
Generalization of the conclusions (ie, appropriate for the data presented and within the domain of the literature review)	13-14
Guidelines for future research	14-15
Disclosure of funding source	8

References for the Supplementary material

1. Alameer K, Abukhzam B, Khan W, El-Saed A, Balkhy H. Middle East respiratory syndrome coronavirus (MERS-Cov) screening of exposed healthcare workers in a tertiary care hospital in Saudi Arabia. Antimicrobial Resistance and Infection Control. 2015;4.
2. Alanazi KH, Killerby ME, Biggs HM, Abedi GR, Jokhdar H, Alsharef AA, et al. Scope and extent of healthcare-associated Middle East respiratory syndrome coronavirus transmission during two contemporaneous outbreaks in Riyadh, Saudi Arabia, 2017. Infection Control and Hospital Epidemiology. 2018;40(1):79-88.
3. Alfaraj SH, Al-Tawfiq JA, Altuwaijri TA, Alanazi M, Alzahrani N, Memish ZA. Middle East respiratory syndrome coronavirus transmission among health care workers: Implication for infection control. American Journal of Infection Control. 2018;46(2):165-8.
4. Alraddadi BM, Al-Salmi HS, Jacobs-Slifka K, Slayton RB, Estivariz CF, Geller AI, et al. Risk factors for middle east respiratory syndrome Coronavirus infection among healthcare personnel. Emerging Infectious Diseases. 2016;22(11):1915-20.
5. Al-Tawfiq JA, Abdrabalnabi R, Taher A, Mathew S, Rahman KA. Infection control influence of Middle East respiratory syndrome coronavirus: A hospital-based analysis. American Journal of Infection Control. 2019;47(4):431-4.
6. Assiri A, McGeer A, Perl TM, Price C, Abdullah R, Cumming D, et al. Hospital Outbreak of Middle East Respiratory Syndrome Coronavirus. New England Journal of Medicine. 2013;369(9):886.
7. 白少丽, 王建云, 周莹荃, 于德生, 高晓敏, 李玲玲, et al. 甘肃省首起新型冠状病毒肺炎家庭聚集性疫情分析 [Analysis of the first cluster of cases in a family of novel coronavirus pneumonia in Gansu Province]. 中华预防医学杂志 [Chin J Prev Med]. 2020;54(04):E005-E.
8. Bai Y, Wang X, Huang Q, Wang H, Gurarie D, Ndeffo-Mbah M, et al. SARS-CoV-2 infection in health care workers: a retrospective analysis and a model study. 2020.
9. Barratt R, Gilbert GL, Shaban RZ, Wyer M, Hor S-y. Enablers of, and barriers to, optimal glove and mask use for routine care in the emergency department: an ethnographic study of Australian clinicians. Australasian Emergency Care. 2019.
10. Baseer M-A, Ansari S-H, AlShamrani S-S, Alakras A-R, Mahrous R, Alenazi A-M. Awareness of droplet and airborne isolation precautions among dental health professionals during the outbreak of corona virus infection in Riyadh city, Saudi Arabia. J Clin Exp Dent. 2016;8:e379-e87.
11. Booth TF, Kournikakis B, Bastien N, Ho J, Kobasa D, Stadnyk L, et al. Detection of airborne severe acute respiratory syndrome (SARS) coronavirus and environmental contamination in SARS outbreak units. J Infect Dis. 2005;191(9):1472-7.
12. 蔡欢乐, 朱言欣, 雷璐碧, 潘程浩, 朱乐玮, 李菁华, et al. 新型冠状病毒肺炎相关知识、行为和心理应对:基于网络的横断面调查 [Novel coronavirus pneumonia epidemic-related knowledge, behaviors and psychology status among college students and their family members and friends: an internet-based cross-sectional survey]. 中国公共卫生 [Chinese Journal of Public Health]. 2020:1-4.
13. 曹培明, 李晓旭, 严晓峰, 刘春玲, 朱建锋, 李雨婷, et al. 重庆市主城区223例新型冠状病毒肺炎病例的回顾性流行病学分析 [23 cases of novel coronavirus in the main city of Chongqing A retrospective epidemiological analysis of pneumonia cases]. 西南大学学报(自然科学版) [Journal of Southwest University(Natural Science Edition)]. 2020;42(3):1-6.
14. Caputo KM, Byrick R, Chapman MG, Orser BA, Orser BJ. Intubation of SARS patients: Infection and perspectives of healthcare workers. Canadian Journal of Anesthesia. 2006;53(2):122-9.
15. Chau JPC, Thompson DR, Lee DTF, Twinn S. Infection control practices among hospital health and support workers in Hong Kong. Journal of Hospital Infection. 2010;75:299-303.
16. Chen YC, Chen PJ, Chang SC, Kao CL, Wang SH, Wang LH, et al. Infection control and SARS transmission among healthcare workers, Taiwan. Br J Ophthalmol. 2004;88(7):861-3. doi: 10.1136/bjo.2003.035931.
17. Chen WQ, Ling WH, Lu CY, Hao YT, Lin ZN, Ling L, et al. Which preventive measures might protect health care workers from SARS? BMC Public Health. 2009;9.
18. 陈燕, 金岳龙, 朱丽君, 方正美, 吴楠, 笃梦雪, et al. 基于网络的安徽省居民新型冠状病毒肺炎知识、态度、行为调查分析 [The network investigation on knowledge, attitude and practice about Novel coronavirus pneumonia of the residents in Anhui Province]. 中华预防医学杂志 [Chin J Prev Med]. 2020;54(4):E004-E.
19. 陈奕, 王爱红, 易波, 丁克琴, 王海波, 王建美, et al. 宁波市新型冠状病毒肺炎密切接触者感染流行病学特征分析 [The epidemiological characteristics of infection in close contacts of COVID-19 in Ningbo city]. 中华流行病学杂志 [Chinese Journal of Epidemiology]. 2020(41):E026-E.
20. 陈夕, 童瑾, 向建华, 胡晶晶. 139例新型冠状病毒肺炎患者流行病学特点对重症化影响的回顾性研究 [Retrospective study on the epidemiological characteristics of 139 patients with novel coronavirus pneumonia on the effects of Severity]. 重庆医学 [Chongqing Medicine]. 2020:1-9.
21. Chen R, Zhang Y, Huang L, Cheng BH, Xia ZY, Meng QT. Safety and efficacy of different anesthetic regimens for parturients with COVID-19 undergoing Cesarean delivery: a case series of 17 patients. Can J Anaesth. 2020;16:16.
22. Cheng VCC, Wong SC, Chen JHK, Yip CCY, Chuang VWM, Tsang OTY, et al. Escalating infection control response to the rapidly evolving epidemiology of the Coronavirus disease 2019 (COVID-19) due to SARS-CoV-2 in Hong Kong. Infect Control Hosp Epidemiol. 2020:1-24.

23. Chia SE, Koh D, Fones C, Qian F, Ng V, Tan BH, et al. Appropriate use of personal protective equipment among healthcare workers in public sector hospitals and primary healthcare polyclinics during the SARS outbreak in Singapore. Occupational and Environmental Medicine. 2005;62(7):473-7.
24. Christian MD, Loutfy M, McDonald LC, Martinez KF, Ofner M, Wong T, et al. Possible SARS Coronavirus Transmission during Cardiopulmonary Resuscitation. Emerging Infectious Diseases. 2004;10(2):287-93.
25. Chughtai AA, Seale H, Chi Dung T, Maher L, Nga PT, MacIntyre CR. Current practices and barriers to the use of facemasks and respirators among hospital-based health care workers in Vietnam. American Journal of Infection Control. 2015;43(1):72-5.
26. Chughtai AA, Seale H, Rawlinson WD, Kunasekaran M, Macintyre CR. Selection and Use of Respiratory Protection by Healthcare Workers to Protect from Infectious Diseases in Hospital Settings. Annals of Work Exposures and Health. 2020.
27. 崔亮亮, 耿兴义, 赵小冬, 杨国樑, 常彩云, 赵梦娇, et al. 济南市现阶段新型冠状病毒肺炎的流行特征与思考 [Reflection and epidemiological characteristics of coronavirus disease 2019 in Jinan City]. 山东大学学报(医学版) [Journal of Shandong University(Health Sciences)]. 2020:1-6.
28. 杜建新. 新疆巴州一起新型冠状病毒肺炎聚集性疫情分析 [A new type of coronavirus pneumonia in Baju, Xinjiang Cluster outbreak analysis]. 疾病预防控制通报 [Bull Dis Control Prev].1-3.
29. El Bushra HE, Abdalla MN, Al Arbash H, Alshayeb Z, Al-Ali S, Al-Abdel Latif Z, et al. An outbreak of middle east respiratory syndrome (MERS) due to coronavirus in Al-Ahssa region, Saudi Arabia, 2015. Eastern Mediterranean Health Journal. 2016;22(7):468-75.
30. Fan C, Liu L, Guo W, Yang A, Ye C, Jilili M, et al. Association between 2019-nCoV transmission and N95 respirator use. Int J Environ Res Public Health. 2020;17(5).(pii):ijerph17051679. doi: 10.3390/ijerph.
31. 冯紫薇, 李润田, 王悦, 尹光雅, 刘竞成, 高文彬, et al. 天津市新型冠状病毒肺炎流行趋势分析 [Analysis of epidemic trend of COVID-19 in Tianjin]. 中华医院感染学杂志 [Chin J Nosocomiol]. 2020;30(11):1-5.
32. Fix GM, Reisinger HS, Etchin A, McDannold S, Eagan A, Findley K, et al. Health care workers' perceptions and reported use of respiratory protective equipment: A qualitative analysis. American Journal of Infection Control. 2019;47(1162-1166).
33. 甘虹, 张一, 袁敏, 伍晓艳, 刘志荣, 刘蒙, et al. 1 052例新型冠状病毒肺炎聚集性病例流行病学特征分析. 中华流行病学杂志. 2020(05):E027-E.
34. Goh DYT, Mun MW, Lee WLJ, Teoh OH, Rajgor DD. A randomised clinical trial to evaluate the safety, fit, comfort of a novel N95 mask in children. Sci Rep. 2019;9:18952.
35. Gomersall CD, Joynt GM, Ho OM, Ip M, Yap F, Derrick JL, et al. Transmission of SARS to healthcare workers. The experience of a Hong Kong ICU. Intensive Care Medicine. 2006;32(4):564-9.
36. Ha LD, Bloom SA, Hien NQ, Maloney SA, Mai LQ, Leitmeyer KC, et al. Lack of SARS Transmission among Public Hospital Workers, Vietnam. Emerging Infectious Diseases. 2004;10(2):265-8.
37. Hall AJ, Tokars JI, Badreddine SA, Saad ZB, Furukawa E, Masri MA, et al. Health care worker contact with MERS patient, Saudi Arabia. Emerging Infectious Diseases. 2014;20(12):2148-51.
38. Hines SE, Brown C, Oliver M, Gucer P, Frisch M, Hogan R, et al. User acceptance of reusable respirators in health care. American Journal of Infection Control. 2019;47(6):648-55.
39. Ho AS, Sung JJ, Chan-Yeung M. An outbreak of severe acute respiratory syndrome among hospital workers in a community hospital in Hong Kong. Ann Intern Med. 2003;139(7):564-7.
40. Ho KY, Singh KS, Habib AG, Ong BK, Lim TK, Ooi EE, et al. Mild illness associated with severe acute respiratory syndrome coronavirus infection: lessons from a prospective seroepidemiologic study of health-care workers in a teaching hospital in Singapore. Hepatology. 2004;39(2):302-10. doi: 10.1002/hep.20111.
41. Ho HSW. Use of face masks in a primary care outpatient setting in Hong Kong: Knowledge, attitudes and practices. Public Health. 2012:1001-6.
42. Honarbakhsh M, Jahangiri M, Ghaem H. Knowledge, perceptions and practices of healthcare workers regarding the use of respiratory protection equipment at Iran hospitals. Journal of infection prevention. 2018;19:29-36.
43. Huang ZL. Investigating the effectiveness of personal respiratory protective equipment used in healthcare workers in southern Taiwan. American Journal of Infection Control. 2011;39(5):E74.
44. Hunter JC, Nguyen D, Aden B, Al Bandar Z, Al Dhaheri W, Abu Elkheir K, et al. Transmission of middle east respiratory syndrome coronavirus infections in healthcare settings, abu dhabi. Emerging Infectious Diseases. 2016;22(4):647-56.
45. Huynh T. "The more I fear about COVID-19, the more I wear medical masks": A survey on risk perception and medical masks' uses. 2020.
46. 贾平, 谢彩霞, 邓瑜萍, 罗聪佩, 陈晓梅. 紧急情况下医用防护面屏的制作与应用 [Self-Designed Medical Protective Shield in Emergency Situation]. 解放军护理杂志 [Nurs J Chin PLA]. 2020;37(2):8-9.
47. 蒋琪霞, 刘玉秀, 魏巍, 陈爱华, 白育瑄, 蔡英华, et al. 新型冠状病毒感染疫情防控期间防护装备所致医护人员皮肤损伤的发生率及流行特征研究 [Incidence and prevalence of skin injury among health care workers due to protective equipment during prevention and control of a novel coronavirus infection epidemic]. 中国全科医学 [Chinese Family Medicine]. 2020;23(09):1083-90.
48. Kang J, Kim EJ, Choi JH, Hong HK, Han S-H, Choi IS, et al. Difficulties in using personal protective equipment: Training experiences with the 2015 outbreak of Middle East respiratory syndrome in Korea. American Journal of Infection Control. 2018;46:235-7.

49. Kao TW, Huang KC, Huang YL, Tsai TJ, Hsieh BS, Wu MS. The physiological impact of wearing an N95 mask during hemodialysis as a precaution against SARS in patients with end-stage renal disease. Journal of the Formosan Medical Association. 2004;103(8):624-8.
50. Khalid I, Khalid TJ, Qabajah MR, Barnard AG, Qushmaq IA. Healthcare workers emotions, perceived stressors and coping strategies during a MERS-CoV outbreak. Clinical Medicine and Research. 2016;14(1):7-14.
51. Khoo KL, Leng PH, Ibrahim IB, Lim TK. The changing face of healthcare worker perceptions on powered air-purifying respirators during the SARS outbreak. Respirology. 2005;10(1):107-10.
52. Ki HK, Han SK, Son JS, Park SO. Risk of transmission via medical employees and importance of routine infection-prevention policy in a nosocomial outbreak of Middle East respiratory syndrome (MERS): a descriptive analysis from a tertiary care hospital in South Korea. J Clin Microbiol. 2019;58(1).(pii):JCM.00963-19. doi: 10.1128/JCM.
53. Kim CJ, Choi WS, Jung Y, Kiem S, Seol HY, Woo HJ, et al. Surveillance of the Middle East respiratory syndrome (MERS) coronavirus (CoV) infection in healthcare workers after contact with confirmed MERS patients: incidence and risk factors of MERS-CoV seropositivity. Clinical Microbiology and Infection. 2016;22(10):880-6.
54. Kinlay J, Flaherty K, Scanlon P, Mehrotra P, Potter-Bynoe G, Sandora TJ. Barriers to the use of face protection for standard precautions by health care providers. Am J Infect Control. 2015;43:169-70.
55. Knapp MB, McIntyre R, Sinkowitz-Cochran RL, Pearson ML. Assessment of health care personnel needs for training in infection control: one size does not fit all. American Journal of Infection Control. 2008;36:757-60.
56. Lau JTF, Yang X, Tsui H, Kim JH. Monitoring community responses to the SARS epidemic in Hong Kong: From day 10 to day 62. Journal of Epidemiology and Community Health. 2003;57(11):864-70.
57. Lau JTF, Lau M, Kim JH, Wong E, Tsui HY, Tsang T, et al. Probable Secondary Infections in Households of SARS Patients in Hong Kong. Emerging Infectious Diseases. 2004;10(2):235-43.
58. Lau JTF, Kim JH, Tsui H, Griffiths S. Anticipated and current preventive behaviors in response to an anticipated human-to-human H5N1 epidemic in the Hong Kong Chinese general population. BMC Infectious Diseases. 2007;7.
59. 李丹, 龙云铸, 黄彭, 郭文龙, 吴双华, 周青, et al. 株洲地区80例新型冠状病毒肺炎患者临床特征分析 [Clnical characteristics of 80 patients with COVID-19 in Zhuzhou City]. 中国感染控制杂志 [Chinese Journal of Infection Control]. 2020;19(3):1-7.
60. 李淑花, 商临萍, 袁丽荣. 山西省新型冠状病毒感染肺炎流行病学分析 [Epidemiological analysis of COVID-19 in Shanxi Province]. 中华医院感染学杂志 [Chin J Nosocomiol]. 2020;30(8):1-5.
61. 李新营, 王琦, 何跃明, 李春辉, 贺吉群, 黄长盛. 新型冠状病毒肺炎患者围手术期处理及防护的认识与思考 %J 中国普通外科杂志 [Understanding and reflection on perioperative management and strategy for prevention and protection in patients with novel coronavirus pneumonia]. [Chinese Journal of General Surgery]. 2020;29(2):142-6.
62. 李陈晨, 马倩倩, 殷环, 赵杰, 翟运开. 河南省新型冠状病毒肺炎高发区流行病学特征及防控分析 [Epidemiological features and prevention measures of CO D 19 in high incidence areas of enan Province]. 武汉大学学报(医学版) [Medical Journal of Wuhan University].1-8.
63. 李锦成, 徐勤, 王艳, 许婕, 黄瑶, 刘文俊, et al. 江苏省扬州市新型冠状病毒肺炎无症状感染者的特征分析 [Analysis in characteristics of asymptomatic infection patients with coronavirus disease 2019 in Yangzhou City of Jiangsu Province]. 实用临床医药杂志 [Journal of Clinical Medicine in Practice]. 2020;24(5):1-4.
64. Li W, Liu B, Liu M, Zhao X, Chen Q. Investigation and analysis of public cognition and prevention awareness of Corona Virus Disease 2019 inside and outside Hubei Province. Journal of Jinan University(Natural Science & Medicine Edition). 2020.
65. Lim PA, Ng YS, Tay BK. Impact of a viral respiratory epidemic on the practice of medicine and rehabilitation: severe acute respiratory syndrome. Arch Phys Med Rehabil. 2004;85(8):1365-70.
66. 林君芬, 吴梦娜, 吴昊澄, 章涛, 吴晨, 李傅冬. 浙江省新型冠状病毒肺炎病例流行特征分析 [Epidemiological characteristics of coronavirus disease 2019 in Zhejiang Province]. 预防医学 [PrevMed，Mar]. 2020;32(3):1-9.
67. Liu W, Tang F, Fang LQ, De Vlas SJ, Ma HJ, Zhou JP, et al. Risk factors for SARS infection among hospital healthcare workers in Beijing: A case control study. Tropical Medicine and International Health. 2009;14(SUPPL. 1):52-9.
68. 刘洁, 罗万军, 邓志宏, 汪小杰, 聂丽, 王文娟, et al. 91例儿童新型冠状病毒肺炎确诊病例临床及流行病学特征. 中华医院感染学杂志.1-5.
69. 刘建中, 陈传文, 夏赛. 15例新型冠状病毒肺炎临床分析. 河南预防医学杂志. 2020;31(03):161-4.
70. 刘广天, 王晓林, 陆敬儒, 赵晓, 雷振华, 李静, et al. 宁夏地区新型冠状病毒肺炎疫情特点分析. 宁夏医科大学学报.1-5.
71. 刘仲, 赵梦娇, 杨国樑, 赵怀龙, 王琳琳, 常彩云, et al. 1例不明原因新型冠状病毒肺炎及其密切接触者调查分析. 山东大学学报(医学版).1-5.

72. 刘郑倩 [Liu ZQ], 叶玉瑶 [Ye YY], 张虹鸥 [Zhang HO], 郭洪旭 [Guo HX], 杨骥 [Yang J], 王长建 [Wang CJ]. 珠海市新型冠状病毒肺炎聚集发生的时空特征及传播路径 [Analysis of the Spatio-Temporal Characteristics and Transmission Path of COVID-19 Cluster Cases in Zhuhai]. 热带地理 [Tropical geography]. 2020:1-13.
73. 刘怡芳, 李佳萌, 周朋辉, 刘静, 董晓春, 吕杰, et al. 天津市新型冠状病毒肺炎聚集性疫情病例分析. 中华流行病学杂志. 2020(05):654-7.
74. Loeb M, McGeer A, Henry B, Ofner M, Rose D, Hlywka T, et al. SARS among Critical Care Nurses, Toronto. Emerging Infectious Diseases. 2004;10(2):251-5.
75. Loh LC, Chelliah A, Ang TH, Ali AM. Change in infection control practices and awareness of hospital medical staff in the aftermath of SARS. The Medical journal of Malaysia. 2004;59(5):659-64.
76. Lu H, Huo N, Xu X, Wang G, Li J, Li H, et al. The epidemiologic characteristics of patients with severe acute respiratory syndrome (SARS). Beijing da xue xue bao Yi xue ban = Journal of Peking University Health sciences. 2003;35 Suppl:8-11.
77. Luo L, Liu D, Liao X, Wu X, Jing Q, Zhen J, et al. Modes of contact and risk of transmission in COVID-19 among close contacts. 2020.
78. Ma HJ, Wang HW, Fang LQ, Jiang JF, Wei MT, Liu W, et al. A case-control study on the risk factors of severe acute respiratory syndromes among health care workers. Zhonghua liu xing bing xue za zhi = Zhonghua liuxingbingxue zazhi. 2004;25(9):741-4.
79. 马耀玲, 夏胜英, 王敏, 张思敏, 杜文辉, 陈琼. 115例新型冠状病毒感染儿童的临床特点分析 [Clinical features of children with SARS-CoV-2 infection: an analysis of 115 cases]. 中国当代儿科杂志 [Chin J Contemp Pediatr]. 2020;22(4):1-4.
80. MacIntyre CR, Seale H, Dung TC, Hien NT, Nga PT, Chughtai AA, et al. A cluster randomised trial of cloth masks compared with medical masks in healthcare workers. BMJ Open. 2015;5:e006577.
81. MacIntyre CR, Zhang Y, Chughtai AA, Seale H, Zhang D, Chu Y, et al. Cluster randomised controlled trial to examine medical mask use as source control for people with respiratory illness. BMJ Open. 2016;6(12):1012330.
82. Marchand-Senecal X, Kozak R, Mubareka S, Salt N, Gubbay JB, Eshaghi A, et al. Diagnosis and Management of First Case of COVID-19 in Canada: Lessons applied from SARS. Clin Infect Dis. 2020;09:09.
83. Maroldi MAC, Felix AMdS, Dias AAL, Kawagoe JY, Padoveze MC, Ferreira SA, et al. Adherence to precautions for preventing the transmission of microorganisms in primary health care: a qualitative study. BMC Nurs. 2017;16(49).
84. Matthews Pillemer F, Blendon RJ, Zaslavsky AM, Lee BY. Predicting support for non-pharmaceutical interventions during infectious outbreaks: a four region analysis. Disasters. 2015;39(1):125-45.
85. Moore DM, Gilbert M, Saunders S, Bryce E, Yassi A. Occupational health and infection control practices related to severe acute respiratory syndrome: health care worker perceptions. AAOHN journal : official journal of the American Association of Occupational Health Nurses. 2005;53(6):257-66.
86. Mukerji S, MacIntyre CR, Seale H, Wang Q, Yang P, Wang X, et al. Cost-effectiveness analysis of N95 respirators and medical masks to protect healthcare workers in China from respiratory infections. BMC Infect Dis. 2017;17(464).
87. Nichol K, Bigelow P, O'Brien-Pallas L, McGeer A, Manno M, Holness DL. The individual, environmental, and organizational factors that influence nurses' use of facial protection to prevent occupational transmission of communicable respiratory illness in acute care hospitals. American Journal of Infection Control. 2008;36:481-7.
88. Nichol K, McGeer A, Bigelow P, O'Brien-Pallas L, Scott J, Holness DL. Behind the mask: Determinants of nurse's adherence to facial protective equipment. American Journal of Infection Control. 2013;41:8-13.
89. Nishiura H, Kuratsuji T, Quy T, Phi NC, Van Ban V, Ha LD, et al. Rapid awareness and transmission of severe acute respiratory syndrome in Hanoi French Hospital, Vietnam. American Journal of Tropical Medicine and Hygiene. 2005;73(1):17-25.
90. Nishiyama A, Wakasugi N, Kirikae T, Quy T, Ha le D, Ban VV, et al. Risk factors for SARS infection within hospitals in Hanoi, Vietnam. Jpn J Infect Dis. 2008;61(5):388-90.
91. Ofner-Agostini M, Gravel D, McDonald LC, Lem M, Sarwal S, McGeer A, et al. Cluster of cases of severe acute respiratory syndrome among Toronto healthcare workers after implementation of infection control precautions: A case series. Infection Control and Hospital Epidemiology. 2006;27(5):473-8.
92. Olsen SJ, Chang HL, Cheung TY, Tang AF, Fisk TL, Ooi SP, et al. Transmission of the severe acute respiratory syndrome on aircraft. N Engl J Med. 2003;349(25):2416-22.
93. Ong SWX, Tan YK, Chia PY, Lee TH, Ng OT, Wong MSY, et al. Air, Surface Environmental, and Personal Protective Equipment Contamination by Severe Acute Respiratory Syndrome Coronavirus 2 (SARS-CoV-2) From a Symptomatic Patient. Jama. 2020;04:04.
94. 欧剑鸣, 叶雯婧, 郑奎城, 李晓庆, 林修全, 卢晓丽, et al. 福建省新型冠状病毒肺炎流行病学特征分析 [Epidemiological characteristics of an outbreak of 2019 novel coronavirus diseases (COVID-19) in FuJian，China]. 中国人兽共患病学报 [Chinese Journal of Zoonoses].1-8.
95. Park BJ, Peck AJ, Kuehnert MJ, Newbern C, Smelser C, Comer JA, et al. Lack of SARS transmission among healthcare workers, United States. Emerg Infect Dis. 2004;10(2):244-8.
96. Park Y-S, Lee C, Kim KM, Kim SW, Lee K-J, Ahn J, et al. The first case of the 2015 Korean Middle East Respiratory Syndrome outbreak. Epidemiol Health. 2015;37.
97. Park JY, Kim BJ, Chung KH, Hwang YI. Factors associated with transmission of middle east respiratory syndrome among korean healthcare workers: Infection control via extended healthcare contact management in a secondary outbreak hospital. Respirology. 2016;21:89.

98. Park J, Yoo SY, Ko JH, Lee SM, Chung YJ, Lee JH, et al. Infection Prevention Measures for Surgical Procedures during a Middle East Respiratory Syndrome Outbreak in a Tertiary Care Hospital in South Korea. Scientific reports. 2020;10(1):325.
99. Parker MJ, Goldman RD. Paediatric emergency department staff perceptions of infection control measures against severe acute respiratory syndrome. Emergency Medicine Journal. 2006;23(5):349-53.
100. Peck AJ, Newbern EC, Feikin DR, Issakbaeva ET, Park BJ, Fehr J, et al. Lack of SARS transmission and U.S. SARS case-patient. Emerg Infect Dis. 2004;10(2):217-24.
101. Pei LY, Gao ZC, Yang Z, Wei DG, Wang SX, Ji JM, et al. Investigation of the influencing factors on severe acute respiratory syndrome among health care workers. Beijing da xue xue bao Yi xue ban = Journal of Peking University Health sciences. 2006;38(3):271-5.
102. 齐晔, 陈刘欢, 张栗, 杨瑛莹, 詹思怡, 傅传喜. 新型冠状病毒感染肺炎的公众认知、态度和行为研究 [Public practice，attitude and knowledge of coronavirus disease]. 热带医学杂志 [J Trop Med]. 2020;20(2):145-9.
103. 钱志成, 宋旭妍, 李姗姗, 金晓博, 刘丹, 刘家盛. 重型及危重型新型冠状病毒肺炎的流行病学和临床特征 [Epidemiological and clinical characteristics analysis of severe and critical corona virus disease 2019]. 武汉大学学报(医学版) [Medical Journal of Wuhan University]. 2020:1-4.
104. Qian G, Yang N, Ma AHY, Wang L, Li G, Chen X. A COVID-19 Transmission within a family cluster by presymptomatic infectors in China. Clin Infect Dis. 2020;23:23.
105. 仇元营, 王松强, 王小丽, 卢伟霞, 乔丹, 李建彬, et al. 一起新型冠状病毒肺炎家庭聚集性疫情的流行病学分析. 中华流行病学杂志. 2020(04):506-9.
106. Rabaan AA, Alhani HM, Bazzi AM, Al-Ahmed SH. Questionnaire-based analysis of infection prevention and control in healthcare facilities in Saudi Arabia in regards to Middle East Respiratory Syndrome. Journal of Infection and Public Health. 2017;10(5):548-63.
107. Radonovich LJ, Simberkoff MS, Bessesen MT, Brown AC, Cummings DAT, Gaydos CA, et al. N95 Respirators vs Medical Masks for Preventing Influenza Among Health Care Personnel: A Randomized Clinical Trial. 322. 2019;9:824-33.
108. Rea E, Laflèche J, Stalker S, Guarda BK, Shapiro H, Johnson I, et al. Duration and distance of exposure are important predictors of transmission among community contacts of Ontario SARS cases. Epidemiology and Infection. 2007;135(6):914-21.
109. Reuss A, Litterst A, Drosten C, Seilmaier M, Bohmer M, Graf P, et al. Contact investigation for imported case of Middle East respiratory syndrome, Germany. Emerg Infect Dis. 2014;20(4):620-5.
110. Reynolds MG, Anh BH, Thu VH, Montgomery JM, Bausch DG, Shah JJ, et al. Factors associated with nosocomial SARS-CoV transmission among healthcare workers in Hanoi, Vietnam, 2003. BMC Public Health. 2006;6:207.
111. Rozenbojm MD, Nichol K, Spielmann S, Holness DL. Hospital unit safety climate: Relationship with nurses' adherence to recommended use of facial protective equipment. American Journal of Infection Control. 2015;43:115-20.
112. Ryu B, Cho SI, Oh MD, Lee JK, Lee J, Hwang YO, et al. Seroprevalence of Middle East respiratory syndrome coronavirus (MERS-CoV) in public health workers responding to a MERS outbreak in Seoul, Republic of Korea, in 2015. Western pac. 2019;10(2):46-8.
113. Scales DC, Green K, Chan AK, Poutanen SM, Foster D, Nowak K, et al. Illness in intensive care staff after brief exposure to severe acute respiratory syndrome. Emerging Infectious Diseases. 2003;9(10):1205-10.
114. Seto WH, Tsang D, Yung RWH, Ching TY, Ng TK, Ho M, et al. Effectiveness of precautions against droplets and contact in prevention of nosocomial transmission of severe acute respiratory syndrome (SARS). Lancet. 2003;361(9368):1519-20.
115. 沈源清, 柯春锦, 杨春光, 李文刚, 胡志全. 武汉同济医院医务人员2019新型冠状病毒感染相关因素的病例对照研究 [A case-control study on 2019-nCov infection-related factors among medical staff in Wuhan Tongji Hospita]. 中华医院感染学杂志 [Chin J Nosocomiol]. 2020;30(8):1-4.
116. Shigayeva A, Green K, Raboud JM, Henry B, Simor AE, Vearncombe M, et al. Factors associated with critical-care healthcare workers' adherence to recommended barrier precautions during the Toronto severe acute respiratory syndrome outbreak. Infection Control and Hospital Epidemiology. 2007;28(11):1275-83.
117. Siu JYM. Qualitative study on the shifting sociocultural meanings of the facemask in Hong Kong since the severe acute respiratory syndrome (SARS) outbreak: Implications for infection control in the post-SARS era. International Journal for Equity in Health. 2016;15(1).
118. 孙倩莱, 李作超, 谭夏林, 蒋永林, 陈伯中, 李谋壹, et al. 一起新型冠状病毒肺炎聚集性疫情调查. 实用预防医学.1-4.
119. Tan NC, Goh LG, Lee SS. Family physicians' experiences, behaviour, and use of personal protection equipment during the SARS outbreak in Singapore: Do they fit the Becker Health Belief Model? Asia-Pacific Journal of Public Health. 2006;18(3):49-56.
120. Tang CSK, Wong CY. Factors influencing the wearing of facemasks to prevent the severe acute respiratory syndrome among adult Chinese in Hong Kong. Preventive Medicine. 2004;39(6):1187-93.
121. Tang JI, Shakespeare TP, Zhang XJ, Lu JJ, Liang S, Wynne CJ, et al. Patient satisfaction with doctor-patient interaction in a radiotherapy centre during the severe acute respiratory syndrome outbreak. Australasian Radiology. 2005;49(4):304-11.
122. Teleman MD, Boudville IC, Heng BH, Zhu D, Leo YS. Factors associated with transmission of severe acute respiratory syndrome among health-care workers in Singapore. Epidemiology and Infection. 2004;132(5):797-803.
123. Tian S, Hu N, Lou J, Chen K, Kang X, Xiang Z, et al. Characteristics of COVID-19 infection in Beijing. J Infect. 2020;80(4):401-6.
124. Timen A, Hulscher MEJL, Rust L, Van Steenbergen JE, Akkermans RP, Grol RPTM, et al. Barriers to implementing infection prevention and control guidelines during crises: Experiences of health care professionals. American Journal of Infection Control. 2010;38(9):726-33.

125. Tuan PA, Horby P, Dinh PN, Mai LT, Zambon M, Shah J, et al. SARS transmission in Vietnam outside of the health-care setting. Epidemiol Infect. 2007;135(3):392-401.
126. Turnberg W, Daniell W, Seixas N, Simpson T, Van Buren J, Lipkin E, et al. Appraisal of recommended respiratory infection control practices in primary care and emergency department settings. American Journal of Infection Control. 2008;36(4):268-75.
127. Twu SJ, Chen TJ, Chen CJ, Olsen SJ, Lee LT, Fisk T, et al. Control measures for severe acute respiratory syndrome (SARS) in Taiwan. Emerging Infectious Diseases. 2003;9(6):718-20.
128. Varia M, Wilson S, Sarwal S, McGeer A, Gournis E, Galanis E, et al. Investigation of a nosocomial outbreak of severe acute respiratory syndrome (SARS) in Toronto, Canada. Canadian Medical Association Journal. 2003;169(4):285-92.
129. Visentin LM, Bondy SJ, Schwartz B, Morrison LJ. Use of personal protective equipment during infectious disease outbreak and nonoutbreak conditions: A survey of emergency medical technicians. Canadian Journal of Emergency Medicine. 2009;11(1):44-56.
130. Wang M, Barasheed O, Rashid H, Booy R, El Bashir H, Haworth E, et al. A cluster-randomised controlled trial to test the efficacy of facemasks in preventing respiratory viral infection among Hajj pilgrims. Journal of Epidemiology and Global Health. 2015;5(2):181-9.
131. 王端, 鞠秀丽, 谢峰, 芦燕, 李飞宇, 黄辉红, et al. 中国北方六省（自治区）儿童2019新型冠状病毒感染31例临床分析 [Clinical analysis of 31 cases of 2019 novel coronavirus infection in children from six provinces (autonomous region) of northern China]. 中华儿科杂志 [Chin J Pediatr]. 2020;58(04):E011-E.
132. 王爱华, 龙泉, 田春. 重庆市渝西地区新冠肺炎确诊病例首发症状及流行病学特征 [Initial symptoms and epidemiological characteristics of confirmed cases of noval coronavirus pneumonia (COVID-19) in the west of Chongqing]. 中国感染控制杂志 [Chinese Journal of Infection Control]. 2020;19(3):1-5.
133. 王小军, 高婧, 王小博, 虎维东, 刘华. 甘肃省新型冠状病毒肺炎病例的临床及流行病学特征 [Clinical and epidemiological characteristics of patients iwth COVID-19 in Gansu Province]. 中国感染控制杂志 [Chinese Journal of Infection Control].19(3):1-4.
134. 王伟, 龚利华. 新冠肺炎疫情期间住院患者陪护人员防护行为调查 [Protection behavior of accompanying visitors of hospitalized patients during COVID-19 epidemic period). 中国感染控制杂志 [Chinese Journal of Infection Control].19(4):1-4.
135. Wiboonchutikul S, Manosuthi W, Likanonsakul S, Sangsajja C, Kongsanan P, Nitiyanontakij R, et al. Lack of transmission among healthcare workers in contact with a case of Middle East respiratory syndrome coronavirus infection in Thailand. Antimicrobial Resistance and Infection Control. 2016;5(1).
136. Wilder-Smith A, Teleman MD, Heng BH, Earnest A, Ling AE, Leo YS. Asymptomatic SARS coronavirus infection among healthcare workers, Singapore. Emerg Infect Dis. 2005;11(7):1142-5.
137. Wizner K, Stradtman L, Novak D, Shaffer R. Prevalence of Respiratory Protective Devices in U.S. Health Care Facilities: Implications for Emergency Preparedness. Workplace Health Saf. 2016;64:359-68.
138. Wong WCW, Lee A, Tsang KK, Wong SYS. How did general practitioners protect themselves, their family, and staff during the SARS epidemic in Hong Kong? Journal of Epidemiology and Community Health. 2004;58(3):180-5.
139. Wong TW, Tam WWS. Handwashing practice and the use of personal protective equipment among medical students after the SARS epidemic in Hong Kong. American Journal of Infection Control. 2005;33(10):580-6.
140. Wong CKM, Yip BHK, Mercer S, Griffiths S, Kung K, Wong MC-s, et al. Effect of facemasks on empathy and relational continuity: a randomised controlled trial in primary care. BMC Fam Pract.
141. Wu J, Xu F, Zhou W, Feikin DR, Lin CY, He X, et al. Risk factors for SARS among persons without known contact with SARS patients, Beijing, China. Emerg Infect Dis. 2004;10(2):210-6.
142. 吴伟慎, 李永刚, 魏兆飞, 周朋辉, 吕莉琨, 张国平, et al. 天津市某百货大楼新型冠状病毒肺炎聚集性疫情调查分析. 中华流行病学杂志. 2020(04):489-93.
143. Wu F, Guo M, Zhang S, Ma Y, Ma P, Duan L, et al. Transmission of 2019-nCoV to health-care workers in the early epidemic. 2020.
144. Wu WS, Li YG, Wei ZF, Zhou PH, Lyu LK, Zhang GP, et al. [Analysis of bronchoscope-guided tracheal intubation in 12 cases with COVID-19 under the personal protective equipment with positive pressure protective hood]. Zhonghua Liu Xing Bing Xue Za Zhi. 2020;41(4):489-93. doi: 10.3760/cma.j.cn112338.
145. 向天新, 刘家明, 许飞, 程娜, 刘洋, 钱克俭, et al. 江西地区49例新型冠状病毒肺炎患者临床特征分析. 中国呼吸与危重监护杂志.1-7.
146. Xiao K, Shiu L, Pang X, Mu H, Wang J, Lang C, et al. The clinical features of the 143 patients with COVID-19 in North-East of Chongqing. Journal of Third Military Medical University. 2020.
147. Xie C, Wang X, Liu H, Bao Z, Yu J, Zhong Y, et al. Infection Control of 2019 Novel Corona Virus Disease (COVID-19) in Cancer Patients undergoing Radiotherapy in Wuhan. 2020.
148. Yang P, Seale H, Raina MacIntyre C, Zhang H, Zhang Z, Zhang Y, et al. Mask-wearing and respiratory infection in healthcare workers in Beijing, China. The Brazilian Journal of Infectious Diseases.
149. 杨海燕, 徐洁, 李岩, 梁璇, 晋乐飞, 陈帅印, et al. 新型冠状病毒肺炎聚集性疫情特征初步分析 [The preliminary analysis on the characteristics of the cluster of the Corona Virus Disease]. 中华流行病学杂志 [Chinese Journal of Epidemiology]. 2020;41(5):623-8.

150. 杨凯, 任敏欢, 肖玲燕, 刘永福, 史东阳, 卢虎, et al. 57例非疫区新型冠状病毒肺炎流行病学及临床特点分析 [Epidemiological and clinical characteristics of 57 cases of new coronavirus pneumonia in non-epidemic areas]. 第三军医大学学报 [Journal of Third Military Medical University]. 2020:1-5.
151. Yin WW, Gao LD, Lin WS, Du L, Zhang XC, Zou Q, et al. Effectiveness of personal protective measures in prevention of nosocomial transmission of severe acute respiratory syndrome. Zhonghua liu xing bing xue za zhi = Zhonghua liuxingbingxue zazhi. 2004;25(1):18-22.
152. Yu ITS, Tze WW, Yuk LC, Lee N, Li Y. Temporal-spatial analysis of severe acute respiratory syndrome among hospital inpatients. Clinical Infectious Diseases. 2005;40(9):1237-43.
153. Yu IT, Zhan HX, Tsoi KK, Yuk LC, Siu WL, Xiao PT, et al. Why did outbreaks of severe acute respiratory syndrome occur in some hospital wards but not in others? Clinical Infectious Diseases. 2007;44(8):1017-25.
154. 余思邈, 王仲霞, 秦恩强, 姜天俊, 景婧, 孙永强, et al. 25例2019冠状病毒病患者临床特征分析. 中国中西医结合杂志.1-2.
155. Yue L, Han L, Li Q, Zhong M, Wang J, Wan Z, et al. Anaesthesia and infection control in cesarean section of pregnant women with coronavirus disease 2019 (COVID-19). 2020.
156. 曾国飞, 杨荟平, 张晓宇, 李雪娇, 杨华. 重庆市中医医疗机构新型冠状病毒肺炎的临床及影像特征分析 [Analysis of Clinical and Imaging Features of Novel Coronavirus Pneumonia in Chongqing Traditional Chinese Medicine System]. 中国中医急症 [Journal of Emergency in Traditional Chinese Medicine]. 2020;29(3):377-80.
157. 张永栋, 郗爱旗, 卓玛, 多杰, 杨正平, 顾玉海, et al. 青海省18例确诊新型冠状病毒肺炎流行病学和感染特征. 中华医院感染学杂志.1-5.
158. 张会会, 冀贞浩, 陈志军, 曾令霞, 米白冰, 陈方尧, et al. 西安市新冠肺炎密切接触者流行特征分析. 西安交通大学学报(医学版).1-7.
159. Zhang G-X, Zhang A-M, Huang L, Cheng L-Y, Liu Z-X, Peng X-L, et al. Twin girls infected with SARS-CoV-2. Chin J Contemp Pediatr. 2020;22(221-5).
160. 赵蕊, 梁运光, 林艳荣, 陆宁, 黎秋连, 李幼玲, et al. 新型冠状病毒肺炎患者28例的临床特征分析 [Clinical characteristics of 28 patients with novel coronavirus pneumonia]. 中华传染病杂志 [Chinese Journal of Infectious Diseases]. 2020(00):E006-E.
161. Zhou Z, Sun X, Li S. Anesthesia management for cesarean section during novel coronavirous epidemic. Chinese Journal of Anesthesiology. 2020;40.
162. 周虹, 朱韩武, 陈柏塘, 郑文, 何德彪, 黄健, et al. 湖南省郴州市Y县一起家庭聚集性新型冠状病毒肺炎疫情调查分析. 上海预防医学.1-4.
163. Zhu Y, Feng H, Luo Y, He T, Lou W. Knowledge, attitude and practice of coronavirus disease 2019

in Ningbo residents. PrevMed，Mar. 2020;32(3):230-4.
164. 庄英杰, 陈竹, 李进, 杨兴龙, 李靖, 袁月, et al. 26例新型冠状病毒肺炎确诊病例临床和流行病学特征 %J 中华医院感染学杂志 [Clinical and epidemiological characteristics of 26 patients diagnosed with COVID-19]. Chin J Nosocomiol. 2020;30(6):1-4.
165. MacIntyre CR, Wang Q, Seale H, Yang P, Shi W, Gao Z, et al. A randomized clinical trial of three options for N95 respirators and medical masks in health workers. Am J Respir Crit Care Med. 2013;187(9):960-6.
166. Zhu Y, Feng H, Luo Y, He T, Lou W. Knowledge, attitude and practice of coronavirus disease 2019 in Ningbo residents. PrevMed，Mar. 2020;32(3):230-4.
167. Pang X, Zhu Z, Xu F, Guo J, Gong X, Liu D, et al. Evaluation of control measures implemented in the severe acute respiratory syndrome outbreak in Beijing, 2003. JAMA. 2003;290(24):3215-21.
168. Wong T-w, Lee C-k, Tam W, Lau JT-f, Yu T-s, Lui S-f, et al. Cluster of SARS among medical students exposed to single patient, Hong Kong. Emerging infectious diseases. 2004;10(2):269-76.
169. Kim T, Jung J, Kim SM, Seo DW, Lee YS, Kim WY, et al. Transmission among healthcare worker contacts with a Middle East respiratory syndrome patient in a single Korean centre. Clin Microbiol Infect. 2016;22(2):e11-e3.
170. Heinzerling A, Stuckey MJ, Scheuer T, Xu K, Perkins KM, Resseger H, et al. Transmission of COVID-19 to Health Care Personnel During Exposures to a Hospitalized Patient - Solano County, California, February 2020. MMWR Morb Mortal Wkly Rep. 2020;69(15):472-6.
171. Burke RM, Balter S, Barnes E, Barry V, Bartlett K, Beer KD, et al. Enhanced Contact Investigations for Nine Early Travel-Related Cases of SARS-CoV-2 in the United States. medRxiv. 2020:2020.04.27.20081901.
172. Wang Q, Huang X, Bai Y, Wang X, Wang H, Hu X, et al. Epidemiological characteristics of COVID-19 in medical staff members of neurosurgery departments in Hubei province: A multicentre descriptive study. medRxiv. 2020:2020.04.20.20064899.
173. Cheng H-Y, Jian S-W, Liu D-P, Ng T-C, Huang W-T, Lin H-H. High transmissibility of COVID-19 near symptom onset. medRxiv. 2020:2020.03.18.20034561.
174. Arwady MA, Alraddadi B, Basler C, Azhar EI, Abuelzein E, Sindy AI, et al. Middle East Respiratory Syndrome Coronavirus Transmission in Extended Family, Saudi Arabia, 2014. Emerging infectious diseases. 2016;22(8):1395-402.

175. Maria DVK, Sadoof A, Abdullah A, Ranawaka APMP, Malik P, Hassan EEB, et al. Transmissibility of MERS-CoV Infection in Closed Setting, Riyadh, Saudi Arabia, 2015. Emerging Infectious Disease journal. 2019;25(10):1802.

Contextual factor studies

Khoo KL, Leng PH, Ibrahim IB, Lim TK. The changing face of healthcare worker perceptions on powered air-purifying respirators during the SARS outbreak. Respirology. 2005;10(1):107-10.
Lim PA, Ng YS, Tay BK. Impact of a viral respiratory epidemic on the practice of medicine and rehabilitation: severe acute respiratory syndrome. Arch Phys Med Rehabil. 2004;85(8):1365-70.
Al-Tawfiq JA, Abdrabalnabi R, Taher A, Mathew S, Rahman KA. Infection control influence of Middle East respiratory syndrome coronavirus: A hospital-based analysis. American Journal of Infection Control. 2019;47(4):431-4.
Maroldi MAC, Felix AMdS, Dias AAL, Kawagoe JY, Padoveze MC, Ferreira SA, et al. Adherence to precautions for preventing the transmission of microorganisms in primary health care: a qualitative study. BMC Nurs. 2017;16(49).
王伟, 龚利华. 新冠肺炎疫情期间住院患者陪护人员防护行为调查 [Protection behavior of accompanying visitors of hospitalized patients during COVID-19 epidemic period). 中国感染控制杂志 [Chinese Journal of Infection Control].19(4):1-4.
Li W, Liu B, Liu M, Zhao X, Chen Q. Investigation and analysis of public cognition and prevention awareness of Corona Virus Disease 2019 inside and outside Hubei Province. Journal of Jinan University(Natural Science & Medicine Edition). 2020.
Zhu Y, Feng H, Luo Y, He T, Lou W. Knowledge, attitude and practice of coronavirus disease 2019 in Ningbo residents. PrevMed， Mar. 2020;32(3):230-4.
齐晔, 陈刘欢, 张栗, 杨瑛莹, 詹思怡, 傅传喜. 新型冠状病毒感染肺炎的公众认知、态度和行为研究 [Public practice， attitude and knowledge of coronavirus disease]. 热带医学杂志 [J Trop Med]. 2020;20(2):145-9.
陈燕, 金岳龙, 朱丽君, 方正美, 吴楠, 笃梦雪, et al. 基于网络的安徽省居民新型冠状病毒肺炎知识、态度、行为调查分析 [The network investigation on knowledge, attitude and practice about Novel coronavirus pneumonia of the residents in Anhui Province]. 中华预防医学杂志 [Chin J Prev Med]. 2020;54(4):E004-E.
Huynh T. “The more I fear about COVID-19, the more I wear medical masks”: A survey on risk perception and medical masks’ uses. 2020.
蔡欢乐, 朱言欣, 雷璐碧, 潘程浩, 朱乐玮, 李菁华, et al. 新型冠状病毒肺炎相关知识、行为和心理应对:基于网络的横断面调查 [Novel coronavirus pneumonia epidemic-related knowledge, behaviors and psychology status among college students and their family members and friends: an internet-based cross-sectional survey]. 中国公共卫生 [Chinese Journal of Public Health]. 2020:1-4.
Lau JTF, Yang X, Tsui H, Kim JH. Monitoring community responses to the SARS epidemic in Hong Kong: From day 10 to day 62. Journal of Epidemiology and Community Health. 2003;57(11):864-70.
Fix GM, Reisinger HS, Etchin A, McDannold S, Eagan A, Findley K, et al. Health care workers’ perceptions and reported use of respiratory protective equipment: A qualitative analysis. American Journal of Infection Control. 2019;47(1162-1166).
Loh LC, Chelliah A, Ang TH, Ali AM. Change in infection control practices and awareness of hospital medical staff in the aftermath of SARS. The Medical journal of Malaysia. 2004;59(5):659-64.
Nichol K, McGeer A, Bigelow P, O'Brien-Pallas L, Scott J, Holness DL. Behind the mask: Determinants of nurse's adherence to facial protective equipment. American Journal of Infection Control. 2013;41:8-13.
Shigayeva A, Green K, Raboud JM, Henry B, Simor AE, Vearncombe M, et al. Factors associated with critical-care healthcare workers' adherence to recommended barrier precautions during the Toronto severe acute respiratory syndrome outbreak. Infection Control and Hospital Epidemiology. 2007;28(11):1275-83.
Baseer M-A, Ansari S-H, AlShamrani S-S, Alakras A-R, Mahrous R, Alenazi A-M. Awareness of droplet and airborne isolation precautions among dental health professionals during the outbreak of corona virus infection in Riyadh city, Saudi Arabia. J Clin Exp Dent. 2016;8:e379-e87.
Chia SE, Koh D, Fones C, Qian F, Ng V, Tan BH, et al. Appropriate use of personal protective equipment among healthcare workers in public sector hospitals and primary healthcare polyclinics during the SARS outbreak in Singapore. Occupational and Environmental Medicine. 2005;62(7):473-7.
Wong TW, Tam WWS. Handwashing practice and the use of personal protective equipment among medical students after the SARS epidemic in Hong Kong. American Journal of Infection Control. 2005;33(10):580-6.
Kang J, Kim EJ, Choi JH, Hong HK, Han S-H, Choi IS, et al. Difficulties in using personal protective equipment: Training experiences with the 2015 outbreak of Middle East respiratory syndrome in Korea. American Journal of Infection Control. 2018;46:235-7.
Tan NC, Goh LG, Lee SS. Family physicians' experiences, behaviour, and use of personal protection equipment during the SARS outbreak in Singapore: Do they fit the Becker Health Belief Model? Asia-Pacific Journal of Public Health. 2006;18(3):49-56.
Moore DM, Gilbert M, Saunders S, Bryce E, Yassi A. Occupational health and infection control practices related to severe acute respiratory syndrome: health care worker perceptions. AAOHN journal : official journal of the American Association of Occupational Health Nurses. 2005;53(6):257-66.
Timen A, Hulscher MEJL, Rust L, Van Steenbergen JE, Akkermans RP, Grol RPTM, et al. Barriers to implementing infection prevention and control guidelines during crises: Experiences of health care professionals. American Journal of Infection Control. 2010;38(9):726-33.

Tang CSK, Wong CY. Factors influencing the wearing of facemasks to prevent the severe acute respiratory syndrome among adult Chinese in Hong Kong. Preventive Medicine. 2004;39(6):1187-93.

Studies addressing distance virus can travel

Booth TF, Kournikakis B, Bastien N, Ho J, Kobasa D, Stadnyk L, et al. Detection of airborne severe acute respiratory syndrome (SARS) coronavirus and environmental contamination in SARS outbreak units. J Infect Dis. 2005;191(9):1472-7.
Cheng VCC, Wong SC, Chen JHK, Yip CCY, Chuang VWM, Tsang OTY, et al. Escalating infection control response to the rapidly evolving epidemiology of the Coronavirus disease 2019 (COVID-19) due to SARS-CoV-2 in Hong Kong. Infect Control Hosp Epidemiol. 2020:1-24.
Christian MD, Loutfy M, McDonald LC, Martinez KF, Ofner M, Wong T, et al. Possible SARS Coronavirus Transmission during Cardiopulmonary Resuscitation. Emerging Infectious Diseases. 2004;10(2):287-93.
Ong SWX, Tan YK, Chia PY, Lee TH, Ng OT, Wong MSY, et al. Air, Surface Environmental, and Personal Protective Equipment Contamination by Severe Acute Respiratory Syndrome Coronavirus 2 (SARS-CoV-2) From a Symptomatic Patient. Jama. 2020;04:04.
Peck AJ, Newbern EC, Feikin DR, Issakbaeva ET, Park BJ, Fehr J, et al. Lack of SARS transmission and U.S. SARS case-patient. Emerg Infect Dis. 2004;10(2):217-24.
Nishiyama A, Wakasugi N, Kirikae T, Quy T, Ha le D, Ban VV, et al. Risk factors for SARS infection within hospitals in Hanoi, Vietnam. Jpn J Infect Dis. 2008;61(5):388-90.
Liu W, Tang F, Fang LQ, De Vlas SJ, Ma HJ, Zhou JP, et al. Risk factors for SARS infection among hospital healthcare workers in Beijing: A case control study. Tropical Medicine and International Health. 2009;14(SUPPL. 1):52-9.
Lau JTF, Lau M, Kim JH, Wong E, Tsui HY, Tsang T, et al. Probable Secondary Infections in Households of SARS Patients in Hong Kong. Emerging Infectious Diseases. 2004;10(2):235-43.
Alraddadi BM, Al-Salmi HS, Jacobs-Slifka K, Slayton RB, Estivariz CF, Geller AI, et al. Risk factors for middle east respiratory syndrome Coronavirus infection among healthcare personnel. Emerging Infectious Diseases. 2016;22(11):1915-20.
Rea E, Laflèche J, Stalker S, Guarda BK, Shapiro H, Johnson I, et al. Duration and distance of exposure are important predictors of transmission among community contacts of Ontario SARS cases. Epidemiology and Infection. 2007;135(6):914-21.
Teleman MD, Boudville IC, Heng BH, Zhu D, Leo YS. Factors associated with transmission of severe acute respiratory syndrome among health-care workers in Singapore. Epidemiology and Infection. 2004;132(5):797-803.
Tuan PA, Horby P, Dinh PN, Mai LT, Zambon M, Shah J, et al. SARS transmission in Vietnam outside of the health-care setting. Epidemiol Infect. 2007;135(3):392-401.
Chen WQ, Ling WH, Lu CY, Hao YT, Lin ZN, Ling L, et al. Which preventive measures might protect health care workers from SARS? BMC Public Health. 2009;9.
Alanazi KH, Killerby ME, Biggs HM, Abedi GR, Jokhdar H, Alsharef AA, et al. Scope and extent of healthcare-associated Middle East respiratory syndrome coronavirus transmission during two contemporaneous outbreaks in Riyadh, Saudi Arabia, 2017. Infection Control and Hospital Epidemiology. 2018;40(1):79-88.
Hall AJ, Tokars JI, Badreddine SA, Saad ZB, Furukawa E, Masri MA, et al. Health care worker contact with MERS patient, Saudi Arabia. Emerging Infectious Diseases. 2014;20(12):2148-51.
Ho AS, Sung JJ, Chan-Yeung M. An outbreak of severe acute respiratory syndrome among hospital workers in a community hospital in Hong Kong. Ann Intern Med. 2003;139(7):564-7.
Ki HK, Han SK, Son JS, Park SO. Risk of transmission via medical employees and importance of routine infection-prevention policy in a nosocomial outbreak of Middle East respiratory syndrome (MERS): a descriptive analysis from a tertiary care hospital in South Korea. J Clin Microbiol. 2019;58(1).(pii):JCM.00963-19. doi: 10.1128/JCM.
Loeb M, McGeer A, Henry B, Ofner M, Rose D, Hlywka T, et al. SARS among Critical Care Nurses, Toronto. Emerging Infectious Diseases. 2004;10(2):251-5.
Ofner-Agostini M, Gravel D, McDonald LC, Lem M, Sarwal S, McGeer A, et al. Cluster of cases of severe acute respiratory syndrome among Toronto healthcare workers after implementation of infection control precautions: A case series. Infection Control and Hospital Epidemiology. 2006;27(5):473-8.
Olsen SJ, Chang HL, Cheung TY, Tang AF, Fisk TL, Ooi SP, et al. Transmission of the severe acute respiratory syndrome on aircraft. N Engl J Med. 2003;349(25):2416-22.
Park JY, Kim BJ, Chung KH, Hwang YI. Factors associated with transmission of middle east respiratory syndrome among korean healthcare workers: Infection control via extended healthcare contact management in a secondary outbreak hospital. Respirology. 2016;21:89.
Pei LY, Gao ZC, Yang Z, Wei DG, Wang SX, Ji JM, et al. Investigation of the influencing factors on severe acute respiratory syndrome among health care workers. Beijing da xue xue bao Yi xue ban = Journal of Peking University Health sciences. 2006;38(3):271-5.
Reuss A, Litterst A, Drosten C, Seilmaier M, Bohmer M, Graf P, et al. Contact investigation for imported case of Middle East respiratory syndrome, Germany. Emerg Infect Dis. 2014;20(4):620-5.
Ryu B, Cho SI, Oh MD, Lee JK, Lee J, Hwang YO, et al. Seroprevalence of Middle East respiratory syndrome coronavirus (MERS-CoV) in public health workers responding to a MERS outbreak in Seoul, Republic of Korea, in 2015. Western pac. 2019;10(2):46-8.
Scales DC, Green K, Chan AK, Poutanen SM, Foster D, Nowak K, et al. Illness in intensive care staff after brief exposure to severe acute respiratory syndrome. Emerging Infectious Diseases. 2003;9(10):1205-10.
Wilder-Smith A, Teleman MD, Heng BH, Earnest A, Ling AE, Leo YS. Asymptomatic SARS coronavirus infection among healthcare workers, Singapore. Emerg Infect Dis. 2005;11(7):1142-5.
Yu ITS, Tze WW, Yuk LC, Lee N, Li Y. Temporal-spatial analysis of severe acute respiratory syndrome among hospital inpatients. Clinical Infectious Diseases. 2005;40(9):1237-43.

Alfaraj SH, Al-Tawfiq JA, Altuwaijri TA, Alanazi M, Alzahrani N, Memish ZA. Middle East respiratory syndrome coronavirus transmission among health care workers: Implication for infection control. American Journal of Infection Control. 2018;46(2):165-8.

Ma HJ, Wang HW, Fang LQ, Jiang JF, Wei MT, Liu W, et al. A case-control study on the risk factors of severe acute respiratory syndromes among health care workers. Zhonghua liu xing bing xue za zhi = Zhonghua liuxingbingxue zazhi. 2004;25(9):741-4.

Bai Y, Wang X, Huang Q, Wang H, Gurarie D, Ndeffo-Mbah M, et al. SARS-CoV-2 infection in health care workers: a retrospective analysis and a model study. 2020.

李新营, 王琦, 何跃明, 李春辉, 贺吉群, 黄长盛. 新型冠状病毒肺炎患者围手术期处理及防护的认识与思考 %J 中国普通外科杂志 [Understanding and reflection on perioperative management and strategy for prevention and protection in patients with novel coronavirus pneumonia]. [Chinese Journal of General Surgery]. 2020;29(2):142-6.

陈奕, 王爱红, 易波, 丁克琴, 王海波, 王建美, et al. 宁波市新型冠状病毒肺炎密切接触者感染流行病学特征分析 [The epidemiological characteristics of infection in close contacts of COVID-19 in Ningbo city]. 中华流行病学杂志 [Chinese Journal of Epidemiology]. 2020(41):E026-E.

崔亮亮, 耿兴义, 赵小冬, 杨国樑, 常彩云, 赵梦娇, et al. 济南市现阶段新型冠状病毒肺炎的流行特征与思考 [Reflection and epidemiological characteristics of coronavirus disease 2019 in Jinan City]. 山东大学学报(医学版) [Journal of Shandong University(Health Sciences)]. 2020:1-6.

庄英杰, 陈竹, 李进, 杨兴龙, 李靖, 袁月, et al. 26例新型冠状病毒肺炎确诊病例临床和流行病学特征 %J 中华医院感染学杂志 [Clinical and epidemiological characteristics of 26 patients diagnosed with COVID-19]. Chin J Nosocomiol. 2020;30(6):1-4.

曹培明, 李晓旭, 严晓峰, 刘春玲, 朱建锋, 李雨婷, et al. 重庆市主城区223例新型冠状病毒肺炎病例的回顾性流行病学分析 [23 cases of novel coronavirus in the main city of Chongqing A retrospective epidemiological analysis of pneumonia cases]. 西南大学学报(自然科学版) [Journal of Southwest University(Natural Science Edition)]. 2020;42(3):1-6.

曾国飞, 杨荟平, 张晓宇, 李雪娇, 杨华. 重庆市中医医疗机构新型冠状病毒肺炎的临床及影像特征分析 [Analysis of Clinical and Imaging Features of Novel Coronavirus Pneumonia in Chongqing Traditional Chinese Medicine System]. 中国中医急症 [Journal of Emergency in Traditional Chinese Medicine]. 2020;29(3):377-80.

李丹, 龙云铸, 黄彭, 郭文龙, 吴双华, 周青, et al. 株洲地区80例新型冠状病毒肺炎患者临床特征分析 [Clnical characteristics of 80 patients with COVID-19 in Zhuzhou City]. 中国感染控制杂志 [Chinese Journal of Infection Control]. 2020;19(3):1-7.

李淑花, 商临萍, 袁丽荣. 山西省新型冠状病毒感染肺炎流行病学分析 [Epidemiological analysis of COVID-19 in Shanxi Province]. 中华医院感染学杂志 [Chin J Nosocomiol]. 2020;30(8):1-5.

蒋琪霞, 刘玉秀, 魏巍, 陈爱华, 白育瑄, 蔡英华, et al. 新型冠状病毒感染疫情防控期间防护装备所致医护人员皮肤损伤的发生率及流行特征研究 [Incidence and prevalence of skin injury among health care workers due to protective equipment during prevention and control of a novel coronavirus infection epidemic]. 中国全科医学 [Chinese Family Medicine]. 2020;23(09):1083-90.

贾平, 谢彩霞, 邓瑜萍, 罗聪佩, 陈晓梅. 紧急情况下医用防护面屏的制作与应用 [Self-Designed Medical Protective Shield in Emergency Situation]. 解放军护理杂志 [Nurs J Chin PLA]. 2020;37(2):8-9.

李陈晨, 马倩倩, 殷环, 赵杰, 翟运开. 河南省新型冠状病毒肺炎高发区流行病学特征及防控分析 [Epidemiological features and prevention measures of CO D 19 in high incidence areas of enan Province]. 武汉大学学报(医学版) [Medical Journal of Wuhan University].1-8.

杨凯, 任敏欢, 肖玲燕, 刘永福, 史东阳, 卢虎, et al. 57例非疫区新型冠状病毒肺炎流行病学及临床特点分析 [Epidemiological and clinical characteristics of 57 cases of new coronavirus pneumonia in non-epidemic areas]. 第三军医大学学报 [Journal of Third Military Medical University]. 2020:1-5.

李锦成, 徐勤, 王艳, 许婕, 黄瑶, 刘文俊, et al. 江苏省扬州市新型冠状病毒肺炎无症状感染者的特征分析 [Analysis in characteristics of asymptomatic infection patients with coronavirus disease 2019 in Yangzhou City of Jiangsu Province]. 实用临床医药杂志 [Journal of Clinical Medicine in Practice]. 2020;24(5):1-4.

杨海燕, 徐洁, 李岩, 梁璇, 晋乐飞, 陈帅印, et al. 新型冠状病毒肺炎聚集性疫情特征初步分析 [The preliminary analysis on the characteristics of the cluster of the Corona Virus Disease]. 中华流行病学杂志 [Chinese Journal of Epidemiology]. 2020;41(5):623-8.

马耀玲, 夏胜英, 王敏, 张思敏, 杜文辉, 陈琼. 115例新型冠状病毒感染儿童的临床特点分析 [Clinical features of children with SARS-CoV-2 infection: an analysis of 115 cases]. 中国当代儿科杂志 [Chin J Contemp Pediatr]. 2020;22(4):1-4.

陈夕, 童瑾, 向建华, 胡晶晶. 139例新型冠状病毒肺炎患者流行病学特点对重症化影响的回顾性研究 [Retrospective study on the epidemiological characteristics of 139 patients with novel coronavirus pneumonia on the effects of Severity]. 重庆医学 [Chongqing Medicine]. 2020:1-9.
钱志成, 宋旭妍, 李姗姗, 金晓博, 刘丹, 刘家盛. 重型及危重型新型冠状病毒肺炎的流行病学和临床特征 [Epidemiological and clinical characteristics analysis of severe and critical corona virus disease 2019]. 武汉大学学报(医学版) [Medical Journal of Wuhan University]. 2020:1-4.
赵蕊, 梁运光, 林艳荣, 陆宁, 黎秋连, 李幼玲, et al. 新型冠状病毒肺炎患者28例的临床特征分析 [Clinical characteristics of 28 patients with novel coronavirus pneumonia]. 中华传染病杂志 [Chinese Journal of Infectious Diseases]. 2020(00):E006-E.
欧剑鸣, 叶雯婧, 郑奎城, 李晓庆, 林修全, 卢晓丽, et al. 福建省新型冠状病毒肺炎流行病学特征分析 [Epidemiological characteristics of an outbreak of 2019 novel coronavirus diseases (COVID-19) in FuJian，China]. 中国人兽共患病学报 [Chinese Journal of Zoonoses].1-8.
沈源清, 柯春锦, 杨春光, 李文刚, 胡志全. 武汉同济医院医务人员2019新型冠状病毒感染相关因素的病例对照研究 [A case-control study on 2019-nCov infection-related factors among medical staff in Wuhan Tongji Hospita]. 中华医院感染学杂志 [Chin J Nosocomiol]. 2020;30(8):1-4.
王小军, 高婧, 王小博, 虎维东, 刘华. 甘肃省新型冠状病毒肺炎病例的临床及流行病学特征 [Clinical and epidemiological characteristics of patients iwth COVID-19 in Gansu Province]. 中国感染控制杂志 [Chinese Journal of Infection Control].19(3):1-4.
王端, 鞠秀丽, 谢峰, 芦燕, 李飞宇, 黄辉红, et al. 中国北方六省（自治区）儿童2019新型冠状病毒感染31例临床分析 [Clinical analysis of 31 cases of 2019 novel coronavirus infection in children from six provinces (autonomous region) of northern China]. 中华儿科杂志 [Chin J Pediatr]. 2020;58(04):E011-E.
白少丽, 王建云, 周莹荃, 于德生, 高晓敏, 李玲玲, et al. 甘肃省首起新型冠状病毒肺炎家庭聚集性疫情分析 [Analysis of the first cluster of cases in a family of novel coronavirus pneumonia in Gansu Province]. 中华预防医学杂志 [Chin J Prev Med]. 2020;54(04):E005-E.
王爱华, 龙泉, 田春. 重庆市渝西地区新冠肺炎确诊病例首发症状及流行病学特征 [Initial symptoms and epidemiological characteristics of confirmed cases of noval coronavirus pneumonia (COVID-19) in the west of Chongqing]. 中国感染控制杂志 [Chinese Journal of Infection Control]. 2020;19(3):1-5.
杜建新. 新疆巴州一起新型冠状病毒肺炎聚集性疫情分析 [A new type of coronavirus pneumonia in Baju, Xinjiang Cluster outbreak analysis]. 疾病预防控制通报 [Bull Dis Control Prev].1-3.
林君芬, 吴梦娜, 吴昊澄, 章涛, 吴晨, 李傅冬. 浙江省新型冠状病毒肺炎病例流行特征分析 [Epidemiological characteristics of coronavirus disease 2019 in Zhejiang Province]. 预防医学 [PrevMed，Mar]. 2020;32(3):1-9.
Wu F, Guo M, Zhang S, Ma Y, Ma P, Duan L, et al. Transmission of 2019-nCoV to health-care workers in the early epidemic. 2020.
Zhou Z, Sun X, Li S. Anesthesia management for cesarean section during novel coronavirous epidemic. Chinese Journal of Anesthesiology. 2020;40.
Xiao K, Shiu L, Pang X, Mu H, Wang J, Lang C, et al. The clinical features of the 143 patients with COVID-19 in North-East of Chongqing. Journal of Third Military Medical University. 2020.
Zhang G-X, Zhang A-M, Huang L, Cheng L-Y, Liu Z-X, Peng X-L, et al. Twin girls infected with SARS-CoV-2. Chin J Contemp Pediatr. 2020;22(221-5).
Huynh T. “The more I fear about COVID-19, the more I wear medical masks”: A survey on risk perception and medical masks’ uses. 2020.
Yue L, Han L, Li Q, Zhong M, Wang J, Wan Z, et al. Anaesthesia and infection control in cesarean section of pregnant women with coronavirus disease 2019 (COVID-19). 2020.
Wong T-w, Lee C-k, Tam W, Lau JT-f, Yu T-s, Lui S-f, et al. Cluster of SARS among medical students exposed to single patient, Hong Kong. Emerging infectious diseases. 2004;10(2):269-76.
Cheng H-Y, Jian S-W, Liu D-P, Ng T-C, Huang W-T, Lin H-H. High transmissibility of COVID-19 near symptom onset. medRxiv. 2020:2020.03.18.20034561.
Arwady MA, Alraddadi B, Basler C, Azhar EI, Abuelzein E, Sindy AI, et al. Middle East Respiratory Syndrome Coronavirus Transmission in Extended Family, Saudi Arabia, 2014. Emerging infectious diseases. 2016;22(8):1395-402.
Maria DVK, Sadoof A, Abdullah A, Ranawaka APMP, Malik P, Hassan EEB, et al. Transmissibility of MERS-CoV Infection in Closed Setting, Riyadh, Saudi Arabia, 2015. Emerging Infectious Disease journal. 2019;25(10):1802.
Heinzerling A, Stuckey MJ, Scheuer T, Xu K, Perkins KM, Resseger H, et al. Transmission of COVID-19 to Health Care Personnel During Exposures to a Hospitalized Patient - Solano County, California, February 2020. MMWR Morb Mortal Wkly Rep. 2020;69(15):472-6.
Burke RM, Balter S, Barnes E, Barry V, Bartlett K, Beer KD, et al. Enhanced Contact Investigations for Nine Early Travel-Related Cases of SARS-CoV-2 in the United States. medRxiv. 2020:2020.04.27.20081901.
Wang Q, Huang X, Bai Y, Wang X, Wang H, Hu X, et al. Epidemiological characteristics of COVID-19 in medical staff members of neurosurgery departments in Hubei province: A multicentre descriptive study. medRxiv. 2020:2020.04.20.20064899.

Mechanistic studies

Booth TF, Kournikakis B, Bastien N, Ho J, Kobasa D, Stadnyk L, et al. Detection of airborne severe acute respiratory syndrome (SARS) coronavirus and environmental contamination in SARS outbreak units. J Infect Dis. 2005;191(9):1472-7.
Cheng VCC, Wong SC, Chen JHK, Yip CCY, Chuang VWM, Tsang OTY, et al. Escalating infection control response to the rapidly evolving epidemiology of the Coronavirus disease 2019 (COVID-19) due to SARS-CoV-2 in Hong Kong. Infect Control Hosp Epidemiol. 2020:1-24.
Christian MD, Loutfy M, McDonald LC, Martinez KF, Ofner M, Wong T, et al. Possible SARS Coronavirus Transmission during Cardiopulmonary Resuscitation. Emerging Infectious Diseases. 2004;10(2):287-93.
Ong SWX, Tan YK, Chia PY, Lee TH, Ng OT, Wong MSY, et al. Air, Surface Environmental, and Personal Protective Equipment Contamination by Severe Acute Respiratory Syndrome Coronavirus 2 (SARS-CoV-2) From a Symptomatic Patient. Jama. 2020;04:04.
Peck AJ, Newbern EC, Feikin DR, Issakbaeva ET, Park BJ, Fehr J, et al. Lack of SARS transmission and U.S. SARS case-patient. Emerg Infect Dis. 2004;10(2):217-24.

Studies addressing the association of mask use and virus transmission

SARS case-patient. Emerg Infect Dis. 2004;10(2):217-24.
Nishiyama A, Wakasugi N, Kirikae T, Quy T, Ha le D, Ban VV, et al. Risk factors for SARS infection within hospitals in Hanoi, Vietnam. Jpn J Infect Dis. 2008;61(5):388-90.
Liu W, Tang F, Fang LQ, De Vlas SJ, Ma HJ, Zhou JP, et al. Risk factors for SARS infection among hospital healthcare workers in Beijing: A case control study. Tropical Medicine and International Health. 2009;14(SUPPL. 1):52-9.
Lau JTF, Lau M, Kim JH, Wong E, Tsui HY, Tsang T, et al. Probable Secondary Infections in Households of SARS Patients in Hong Kong. Emerging Infectious Diseases. 2004;10(2):235-43.
Alraddadi BM, Al-Salmi HS, Jacobs-Slifka K, Slayton RB, Estivariz CF, Geller AI, et al. Risk factors for middle east respiratory syndrome Coronavirus infection among healthcare personnel. Emerging Infectious Diseases. 2016;22(11):1915-20.
Teleman MD, Boudville IC, Heng BH, Zhu D, Leo YS. Factors associated with transmission of severe acute respiratory syndrome among health-care workers in Singapore. Epidemiology and Infection. 2004;132(5):797-803.
Tuan PA, Horby P, Dinh PN, Mai LT, Zambon M, Shah J, et al. SARS transmission in Vietnam outside of the health-care setting. Epidemiol Infect. 2007;135(3):392-401.
Chen WQ, Ling WH, Lu CY, Hao YT, Lin ZN, Ling L, et al. Which preventive measures might protect health care workers from SARS? BMC Public Health. 2009;9.
Alanazi KH, Killerby ME, Biggs HM, Abedi GR, Jokhdar H, Alsharef AA, et al. Scope and extent of healthcare-associated Middle East respiratory syndrome coronavirus transmission during two contemporaneous outbreaks in Riyadh, Saudi Arabia, 2017. Infection Control and Hospital Epidemiology. 2018;40(1):79-88.
Hall AJ, Tokars JI, Badreddine SA, Saad ZB, Furukawa E, Masri MA, et al. Health care worker contact with MERS patient, Saudi Arabia. Emerging Infectious Diseases. 2014;20(12):2148-51.
Ho AS, Sung JJ, Chan-Yeung M. An outbreak of severe acute respiratory syndrome among hospital workers in a community hospital in Hong Kong. Ann Intern Med. 2003;139(7):564-7.
Ki HK, Han SK, Son JS, Park SO. Risk of transmission via medical employees and importance of routine infection-prevention policy in a nosocomial outbreak of Middle East respiratory syndrome (MERS): a descriptive analysis from a tertiary care hospital in South Korea. J Clin Microbiol. 2019;58(1).(pii):JCM.00963-19. doi: 10.1128/JCM.
Loeb M, McGeer A, Henry B, Ofner M, Rose D, Hlywka T, et al. SARS among Critical Care Nurses, Toronto. Emerging Infectious Diseases. 2004;10(2):251-5.
Ofner-Agostini M, Gravel D, McDonald LC, Lem M, Sarwal S, McGeer A, et al. Cluster of cases of severe acute respiratory syndrome among Toronto healthcare workers after implementation of infection control precautions: A case series. Infection Control and Hospital Epidemiology. 2006;27(5):473-8.
Park JY, Kim BJ, Chung KH, Hwang YI. Factors associated with transmission of middle east respiratory syndrome among korean healthcare workers: Infection control via extended healthcare contact management in a secondary outbreak hospital. Respirology. 2016;21:89.
Pei LY, Gao ZC, Yang Z, Wei DG, Wang SX, Ji JM, et al. Investigation of the influencing factors on severe acute respiratory syndrome among health care workers. Beijing da xue xue bao Yi xue ban = Journal of Peking University Health sciences. 2006;38(3):271-5.
Scales DC, Green K, Chan AK, Poutanen SM, Foster D, Nowak K, et al. Illness in intensive care staff after brief exposure to severe acute respiratory syndrome. Emerging Infectious Diseases. 2003;9(10):1205-10.
Wilder-Smith A, Teleman MD, Heng BH, Earnest A, Ling AE, Leo YS. Asymptomatic SARS coronavirus infection among healthcare workers, Singapore. Emerg Infect Dis. 2005;11(7):1142-5.
Fan C, Liu L, Guo W, Yang A, Ye C, Jilili M, et al. Association between 2019-nCoV transmission and N95 respirator use. Int J Environ Res Public Health. 2020;17(5).(pii):ijerph17051679. doi: 10.3390/ijerph.
Seto WH, Tsang D, Yung RWH, Ching TY, Ng TK, Ho M, et al. Effectiveness of precautions against droplets and contact in prevention of nosocomial transmission of severe acute respiratory syndrome (SARS). Lancet. 2003;361(9368):1519-20.
Nishiura H, Kuratsuji T, Quy T, Phi NC, Van Ban V, Ha LD, et al. Rapid awareness and transmission of severe acute respiratory syndrome in Hanoi French Hospital, Vietnam. American Journal of Tropical Medicine and Hygiene. 2005;73(1):17-25.
Yin WW, Gao LD, Lin WS, Du L, Zhang XC, Zou Q, et al. Effectiveness of personal protective measures in prevention of nosocomial transmission of severe acute respiratory syndrome. Zhonghua liu xing bing xue za zhi = Zhonghua liuxingbingxue zazhi. 2004;25(1):18-22.

Caputo KM, Byrick R, Chapman MG, Orser BA, Orser BJ. Intubation of SARS patients: Infection and perspectives of healthcare workers. Canadian Journal of Anesthesia. 2006;53(2):122-9.
Gomersall CD, Joynt GM, Ho OM, Ip M, Yap F, Derrick JL, et al. Transmission of SARS to healthcare workers. The experience of a Hong Kong ICU. Intensive Care Medicine. 2006;32(4):564-9.
Ha LD, Bloom SA, Hien NQ, Maloney SA, Mai LQ, Leitmeyer KC, et al. Lack of SARS Transmission among Public Hospital Workers, Vietnam. Emerging Infectious Diseases. 2004;10(2):265-8.
Kim CJ, Choi WS, Jung Y, Kiem S, Seol HY, Woo HJ, et al. Surveillance of the Middle East respiratory syndrome (MERS) coronavirus (CoV) infection in healthcare workers after contact with confirmed MERS patients: incidence and risk factors of MERS-CoV seropositivity. Clinical Microbiology and Infection. 2016;22(10):880-6.
Mukerji S, MacIntyre CR, Seale H, Wang Q, Yang P, Wang X, et al. Cost-effectiveness analysis of N95 respirators and medical masks to protect healthcare workers in China from respiratory infections. BMC Infect Dis. 2017;17(464).
Pang X, Zhu Z, Xu F, Guo J, Gong X, Liu D, et al. Evaluation of control measures implemented in the severe acute respiratory syndrome outbreak in Beijing, 2003. JAMA. 2003;290(24):3215-21.
Park BJ, Peck AJ, Kuehnert MJ, Newbern C, Smelser C, Comer JA, et al. Lack of SARS transmission among healthcare workers, United States. Emerg Infect Dis. 2004;10(2):244-8.
Wang M, Barasheed O, Rashid H, Booy R, El Bashir H, Haworth E, et al. A cluster-randomised controlled trial to test the efficacy of facemasks in preventing respiratory viral infection among Hajj pilgrims. Journal of Epidemiology and Global Health. 2015;5(2):181-9.
Wu F, Guo M, Zhang S, Ma Y, Ma P, Duan L, et al. Transmission of 2019-nCoV to health-care workers in the early epidemic. 2020.
Wong T-w, Lee C-k, Tam W, Lau JT-f, Yu T-s, Lui S-f, et al. Cluster of SARS among medical students exposed to single patient, Hong Kong. Emerging infectious diseases. 2004;10(2):269-76.
Kim T, Jung J, Kim SM, Seo DW, Lee YS, Kim WY, et al. Transmission among healthcare worker contacts with a Middle East respiratory syndrome patient in a single Korean centre. Clin Microbiol Infect. 2016;22(2):e11-e3.
Heinzerling A, Stuckey MJ, Scheuer T, Xu K, Perkins KM, Resseger H, et al. Transmission of COVID-19 to Health Care Personnel During Exposures to a Hospitalized Patient - Solano County, California, February 2020. MMWR Morb Mortal Wkly Rep. 2020;69(15):472-6.
Burke RM, Balter S, Barnes E, Barry V, Bartlett K, Beer KD, et al. Enhanced Contact Investigations for Nine Early Travel-Related Cases of SARS-CoV-2 in the United States. medRxiv. 2020:2020.04.27.20081901.
Wang Q, Huang X, Bai Y, Wang X, Wang H, Hu X, et al. Epidemiological characteristics of COVID-19 in medical staff members of neurosurgery departments in Hubei province: A multicentre descriptive study. medRxiv. 2020:2020.04.20.20064899.

Studies addressing the association of eye protection with virus transmission

Peck AJ, Newbern EC, Feikin DR, Issakbaeva ET, Park BJ, Fehr J, et al. Lack of SARS transmission and U.S. SARS case-patient. Emerg Infect Dis. 2004;10(2):217-24.
Alraddadi BM, Al-Salmi HS, Jacobs-Slifka K, Slayton RB, Estivariz CF, Geller AI, et al. Risk factors for middle east respiratory syndrome Coronavirus infection among healthcare personnel. Emerging Infectious Diseases. 2016;22(11):1915-20.
Chen WQ, Ling WH, Lu CY, Hao YT, Lin ZN, Ling L, et al. Which preventive measures might protect health care workers from SARS? BMC Public Health. 2009;9.
Alanazi KH, Killerby ME, Biggs HM, Abedi GR, Jokhdar H, Alsharef AA, et al. Scope and extent of healthcare-associated Middle East respiratory syndrome coronavirus transmission during two contemporaneous outbreaks in Riyadh, Saudi Arabia, 2017. Infection Control and Hospital Epidemiology. 2018;40(1):79-88.
Hall AJ, Tokars JI, Badreddine SA, Saad ZB, Furukawa E, Masri MA, et al. Health care worker contact with MERS patient, Saudi Arabia. Emerging Infectious Diseases. 2014;20(12):2148-51.
Ki HK, Han SK, Son JS, Park SO. Risk of transmission via medical employees and importance of routine infection-prevention policy in a nosocomial outbreak of Middle East respiratory syndrome (MERS): a descriptive analysis from a tertiary care hospital in South Korea. J Clin Microbiol. 2019;58(1).(pii):JCM.00963-19. doi: 10.1128/JCM.
Ofner-Agostini M, Gravel D, McDonald LC, Lem M, Sarwal S, McGeer A, et al. Cluster of cases of severe acute respiratory syndrome among Toronto healthcare workers after implementation of infection control precautions: A case series. Infection Control and Hospital Epidemiology. 2006;27(5):473-8.
Park JY, Kim BJ, Chung KH, Hwang YI. Factors associated with transmission of middle east respiratory syndrome among korean healthcare workers: Infection control via extended healthcare contact management in a secondary outbreak hospital. Respirology. 2016;21:89.
Pei LY, Gao ZC, Yang Z, Wei DG, Wang SX, Ji JM, et al. Investigation of the influencing factors on severe acute respiratory syndrome among health care workers. Beijing da xue xue bao Yi xue ban = Journal of Peking University Health sciences. 2006;38(3):271-5.
Ryu B, Cho SI, Oh MD, Lee JK, Lee J, Hwang YO, et al. Seroprevalence of Middle East respiratory syndrome coronavirus (MERS-CoV) in public health workers responding to a MERS outbreak in Seoul, Republic of Korea, in 2015. Western pac. 2019;10(2):46-8.
Ma HJ, Wang HW, Fang LQ, Jiang JF, Wei MT, Liu W, et al. A case-control study on the risk factors of severe acute respiratory syndromes among health care workers. Zhonghua liu xing bing xue za zhi = Zhonghua liuxingbingxue zazhi. 2004;25(9):741-4.
Yin WW, Gao LD, Lin WS, Du L, Zhang XC, Zou Q, et al. Effectiveness of personal protective measures in prevention of nosocomial transmission of severe acute respiratory syndrome. Zhonghua liu xing bing xue za zhi = Zhonghua liuxingbingxue zazhi. 2004;25(1):18-22.
Caputo KM, Byrick R, Chapman MG, Orser BA, Orser BJ. Intubation of SARS patients: Infection and perspectives of healthcare workers. Canadian Journal of Anesthesia. 2006;53(2):122-9.
Chen R, Zhang Y, Huang L, Cheng BH, Xia ZY, Meng QT. Safety and efficacy of different anesthetic regimens for parturients with COVID-19 undergoing Cesarean delivery: a case series of 17 patients. Can J Anaesth. 2020;16:16.

Kim CJ, Choi WS, Jung Y, Kiem S, Seol HY, Woo HJ, et al. Surveillance of the Middle East respiratory syndrome (MERS) coronavirus (CoV) infection in healthcare workers after contact with confirmed MERS patients: incidence and risk factors of MERS-CoV seropositivity. Clinical Microbiology and Infection. 2016;22(10):880-6.
Pang X, Zhu Z, Xu F, Guo J, Gong X, Liu D, et al. Evaluation of control measures implemented in the severe acute respiratory syndrome outbreak in Beijing, 2003. JAMA. 2003;290(24):3215-21.
Park BJ, Peck AJ, Kuehnert MJ, Newbern C, Smelser C, Comer JA, et al. Lack of SARS transmission among healthcare workers, United States. Emerg Infect Dis. 2004;10(2):244-8.
Wu WS, Li YG, Wei ZF, Zhou PH, Lyu LK, Zhang GP, et al. [Analysis of bronchoscope-guided tracheal intubation in 12 cases with COVID-19 under the personal protective equipment with positive pressure protective hood]. Zhonghua Liu Xing Bing Xue Za Zhi. 2020;41(4):489-93. doi: 10.3760/cma.j.cn112338.
Burke RM, Balter S, Barnes E, Barry V, Bartlett K, Beer KD, et al. Enhanced Contact Investigations for Nine Early Travel-Related Cases of SARS-CoV-2 in the United States. medRxiv. 2020:2020.04.27.20081901.
